# Supplementary material for: Prevalence and predictors of spontaneous preterm births in Nepal: findings from a prospective, population-based pregnancy cohort in rural Nepal–a secondary data analysis
Source: BMJ Open. 2022 Dec 1;12(12):e066934. doi: 10.1136/bmjopen-2022-066934 (PMC9716942; doi:10.1136/bmjopen-2022-066934)
Supplement: Supplementary data [file bmjopen-2022-066934supp001.pdf]

SUPPLEMENTARY TABLES

Table S1. Comparing Pregnancy non-varying Variables by pregnancies Included and Excluded in the Regression Analysis

Variables	Categories	Total<br><br>N=30,758	Included in regression<br><br>N=21,297	Excluded in regression<br><br>N=9,461	p-value
		N (%)	N (%)	N (%)	
Maternal Age at LMP	18 to 35	25,300 (82.3)	17,683 (83.0)	7,617 (80.5)	<0.001
	Less than 18	4,792 (15.6)	3,169 (14.9)	1,623 (17.2)	
	More than 35	666 (2.2)	445 (2.1)	221 (2.3)	
Caste/Ethnicity Categories	Brahmin and Chhetri	879 (2.9)	661 (3.1)	218 (2.3)	<0.001
	Vaishya	22,104 (71.9)	15,412 (72.4)	6,692 (70.7)	
	Shudra	4,826 (15.7)	3,392 (15.9)	1,434 (15.2)	
	Muslim and others	2,919 (9.5)	1,832 (8.6)	1,087 (11.5)	
	Missing	30 (0.1)	0 (0.0)	30 (0.3)	
Mother's Education	No schooling	20,891 (67.9)	14,561 (68.4)	6,330 (66.9)	0.032
	1 to 5 years	2,613 (8.5)	1,819 (8.5)	794 (8.4)	
	More than 5 years	7,224 (23.5)	4,917 (23.1)	2,307 (24.4)	
	Missing	30 (0.1)	0 (0.0)	30 (0.3)	
Quintiles of Wealth	Poorest	6,354 (20.7)	4,414 (20.7)	1,940 (20.5)	0.004
	Poorer	6,210 (20.2)	4,386 (20.6)	1,824 (19.3)	
	Middle	6,152 (20.0)	4,289 (20.1)	1,863 (19.7)	
	Richer	6,036 (19.6)	4,172 (19.6)	1,864 (19.7)	
	Richest	5,985 (19.5)	4,036 (19.0)	1,949 (20.6)	
	Missing	21 (0.1)	0 (0.0)	21 (0.2)	
Maternal Height (centimeter)	<145	4,510 (14.7)	3,193 (15.0)	1,317 (13.9)	0.042
	145-<150	9,227 (30.0)	6,413 (30.1)	2,814 (29.7)	
	>=150	16,974 (55.2)	11,691 (54.9)	5,283 (55.8)	
	Missing	47 (0.2)	0 (0.0)	47 (0.5)	
Parity including both LB and SB, at Enrollment	Parity 1 to 4	19,805 (64.4)	14,137 (66.4)	5,668 (59.9)	<0.001
	More than 4	1,351 (4.4)	875 (4.1)	476 (5.0)	
	Prior Pregnant but parity 0	723 (2.4)	515 (2.4)	208 (2.2)	
	No Prior Pregnant	8,717 (28.3)	5,770 (27.1)	2,947 (31.1)	
	Missing	162 (0.5)	0 (0.0)	162 (1.7)	
Interpregnancy Interval based on maternal recall	18 to 36 months	7,723 (25.1)	5,540 (26.0)	2,183 (23.1)	<0.001
	Less than 18 months	11,201 (36.4)	7,693 (36.1)	3,508 (37.1)	
	More than 36 months	3,106 (10.1)	2,294 (10.8)	812 (8.6)	
	No Prior Pregnancy	8,717 (28.3)	5,770 (27.1)	2,947 (31.1)	
	Missing	11 (0.0)	0 (0.0)	11 (0.1)	
Any deaths among Prior LB	Prior LB but not died	17,089 (55.6)	12,273 (57.6)	4,816 (50.9)	<0.001
	Prior LB died	3,518 (11.4)	2,555 (12.0)	963 (10.2)	
	Prior Pregnancy but no LB	980 (3.2)	699 (3.3)	281 (3.0)	
	No prior pregnancy	8,717 (28.3)	5,770 (27.1)	2,947 (31.1)	
	Missing	454 (1.5)	0 (0.0)	454 (4.8)	
Any prior pregnancy ended in SB	Prior pregnancy but no SB	20,736 (67.4)	14,704 (69.0)	6,032 (63.8)	<0.001

Variables	Categories	Total<br>N=30,758	Included in regression<br>N=21,297	Excluded in regression<br>N=9,461	p-value
	Prior SB	1,291 (4.2)	815 (3.8)	476 (5.0)	
	No prior pregnancy	8,717 (28.3)	5,770 (27.1)	2,947 (31.1)	
	Missing	14 (0.0)	8 (0.0)	6 (0.1)	
Any prior pregnancy ended in miscarriage?	Prior pregnancy but no miscarriage	18,554 (60.3)	12,959 (60.8)	5,595 (59.1)	<0.001
	Prior miscarriage	3,478 (11.3)	2,565 (12.0)	913 (9.7)	
	No prior pregnancy	8,717 (28.3)	5,770 (27.1)	2,947 (31.1)	
	Missing	9 (0.0)	3 (0.0)	6 (0.1)	
Any prior pregnancy ended in multiples?	Prior pregnancy but no multiples	21,735 (70.7)	15,383 (72.2)	6,352 (67.1)	<0.001
	Prior multiples	286 (0.9)	135 (0.6)	151 (1.6)	
	No prior pregnancy	8,717 (28.3)	5,770 (27.1)	2,947 (31.1)	
	Missing	20 (0.1)	9 (0.0)	11 (0.1)	
Number of ANC visits	No visit	5,431 (17.7)	3,788 (17.8)	1,643 (17.4)	<0.001
	1 visit	4,047 (13.2)	2,836 (13.3)	1,211 (12.8)	
	2-3 visit	9,443 (30.7)	6,809 (32.0)	2,634 (27.8)	
	4 or more	8,342 (27.1)	6,532 (30.7)	1,810 (19.1)	
	Missing	3,495 (11.4)	1,332 (6.3)	2,163 (22.9)	
Place of Delivery	Home/Maiti	15,669 (50.9)	11,348 (53.3)	4,321 (45.7)	0.002
	HP/Clinic/Hospital	11,038 (35.9)	8,210 (38.6)	2,828 (29.9)	
	Way to Facility/Outdoors	602 (2.0)	439 (2.1)	163 (1.7)	
	Missing	3,449 (11.2)	1,300 (6.1)	2,149 (22.7)	
Bidi or tobacco use in pregnancy	No	30,410 (98.9)	21,060 (98.9)	9,350 (98.8)	0.64
	Yes	348 (1.1)	237 (1.1)	111 (1.2)	
Alcohol use (jaard or rakshi) in pregnancy?	No	30,665 (99.7)	21,230 (99.7)	9,435 (99.7)	0.56
	Yes	93 (0.3)	67 (0.3)	26 (0.3)	
Multiple Birth	Singleton	30,508 (99.2)	21,147 (99.3)	9,361 (98.9)	0.001
	Twin/Triplet	250 (0.8)	150 (0.7)	100 (1.1)	
Sex of the child	Female	14,673 (47.7)	10,178 (47.8)	4,495 (47.5)	0.004
	Male	15,736 (51.2)	10,969 (51.5)	4,767 (50.4)	
	Twin/Triplet	250 (0.8)	150 (0.7)	100 (1.1)	
	Missing	99 (0.3)	0 (0.0)	99 (1.0)	
Preterm Birth	Term	26,130 (85.0)	18,363 (86.2)	7,767 (82.1)	<0.001
	Preterm	4,628 (15.0)	2,934 (13.8)	1,694 (17.9)	
Gestational Age at outcome in weeks (Mean (SD))		39.4 (3.4)	39.5 (2.7)	39.2 (4.6)	<0.001

Table S2-Comparing the Adjusted Risk Ratios for associations between risk factors and spontaneous preterm birth in different models

**Name of Variables**	**Categories**	**Model 1**<br>**Unadjusted Model**<br><br>Risk Ratio (95%CI)	**Model 2**<br>**Adjusted Model without ANC/Place of Delivery**<br>**(N=21,297)**<br><br>Risk Ratio (95%CI)	**Model 3**<br>**Adjusted- Added ANC**<br><br>**(N=19,965)**<br><br>Risk Ratio (95%CI)	**Model 4**<br>**Adjusted- Added ANC and Place of Delivery**<br><br>**(N=19,964)**<br><br>Risk Ratio (95%CI)
Maternal Age at LMP	18 to 35	1.00 [1.00,1.00]	1.00 [1.00,1.00]	1.00 [1.00,1.00]	1.00 [1.00,1.00]
	Less than 18	1.19*** [1.11,1.28]	1.13* [1.02,1.26]	1.11 [1.00,1.24]	1.11* [1.00,1.24]
	More than 35	1.57*** [1.36,1.81]	1.22 [0.98,1.51]	1.20 [0.97,1.49]	1.20 [0.97,1.49]
Caste/Ethnicity Categories	Brahmin and Chhetri	1.00 [1.00,1.00]	1.00 [1.00,1.00]	1.00 [1.00,1.00]	1.00 [1.00,1.00]
	Vaishya	1.33** [1.09,1.62]	1.23 [0.95,1.59]	1.19 [0.92,1.54]	1.20 [0.92,1.54]
	Shudra	1.55*** [1.26,1.90]	1.23 [0.94,1.62]	1.18 [0.90,1.55]	1.18 [0.90,1.55]
	Muslim and others	1.96*** [1.60,2.42]	1.53** [1.16,2.01]	1.53** [1.16,2.01]	1.53** [1.16,2.02]
Mother's Years of Education	No schooling	1.00 [1.00,1.00]	1.00 [1.00,1.00]	1.00 [1.00,1.00]	1.00 [1.00,1.00]
	1 to 5 years	0.86** [0.78,0.95]	0.91 [0.80,1.03]	0.95 [0.83,1.08]	0.95 [0.83,1.08]
	More than 5 years	0.71*** [0.66,0.76]	0.81*** [0.73,0.90]	0.85** [0.77,0.95]	0.85** [0.76,0.94]
Quintiles of Wealth	Poorest	1.00 [1.00,1.00]	1.00 [1.00,1.00]	1.00 [1.00,1.00]	1.00 [1.00,1.00]
	Poorer	0.86*** [0.79,0.93]	0.90* [0.82,1.00]	0.91 [0.83,1.01]	0.91 [0.83,1.01]
	Middle	0.89** [0.82,0.96]	0.95 [0.86,1.05]	0.98 [0.88,1.08]	0.97 [0.88,1.08]
	Richer	0.73*** [0.67,0.79]	0.83** [0.74,0.93]	0.88* [0.78,0.98]	0.87* [0.78,0.98]
	Richest	0.71*** [0.65,0.77]	0.88* [0.78,1.00]	0.91 [0.80,1.03]	0.90 [0.80,1.02]
Mother's height(centimeter)	<145	1.00 [1.00,1.00]	1.00 [1.00,1.00]	1.00 [1.00,1.00]	1.00 [1.00,1.00]
	145-<150	0.93 [0.86,1.01]	0.98 [0.88,1.08]	0.98 [0.89,1.09]	0.99 [0.89,1.09]
	>=150	0.81*** [0.75,0.87]	0.89* [0.81,0.98]	0.90* [0.82,1.00]	0.91 [0.82,1.00]
Parity including both LB and SB, at Enrollment	Parity 1 to 4	1.00 [1.00,1.00]	1.00 [1.00,1.00]	1.00 [1.00,1.00]	1.00 [1.00,1.00]
	More than 4	1.32*** [1.17,1.48]	1.17 [0.99,1.37]	1.11 [0.95,1.31]	1.12 [0.95,1.31]
	Prior Pregnant but parity 0	1.02 [0.85,1.22]	0.92 [0.62,1.37]	1.12 [0.73,1.73]	1.11 [0.72,1.72]
	No Prior Pregnant	1.10** [1.04,1.17]	1.15** [1.04,1.28]	1.20** [1.07,1.34]	1.19** [1.07,1.33]
Interpregnancy Intervals	18 to 36 months	1.00 [1.00,1.00]	1.00 [1.00,1.00]	1.00 [1.00,1.00]	1.00 [1.00,1.00]
	Less than 18 months	1.07 [0.99,1.14]	1.08 [0.99,1.18]	1.09 [0.99,1.19]	1.09 [0.99,1.19]
	More than 36 months	0.98 [0.89,1.09]	0.90 [0.79,1.02]	0.93 [0.82,1.06]	0.93 [0.82,1.06]
	No Prior Pregnancy	1.11** [1.03,1.20]	1.00 [1.00,1.00]	1.00 [1.00,1.00]	1.00 [1.00,1.00]
Any death among prior LB	Prior LB but not died	1.00 [1.00,1.00]	1.00 [1.00,1.00]	1.00 [1.00,1.00]	1.00 [1.00,1.00]
	Prior LB died	1.19*** [1.09,1.29]	1.07 [0.97,1.19]	1.07 [0.96,1.20]	1.07 [0.96,1.20]
	Prior Pregnancy but no LB	1.07 [0.92,1.25]	1.06 [0.75,1.49]	0.97 [0.66,1.41]	0.96 [0.66,1.41]
	No prior pregnancy	1.12*** [1.06,1.19]			

**Name of Variables**	**Categories**	**Model 1 Unadjusted Model**<br><br>Risk Ratio (95%CI)	**Model 2 Adjusted Model without ANC/Place of Delivery (N=21,297)**<br><br>Risk Ratio (95%CI)	**Model 3 Adjusted- Added ANC**<br><br>**(N=19,965)**<br><br>Risk Ratio (95%CI)	**Model 4 Adjusted- Added ANC and Place of Delivery**<br><br>**(N=19,964)**<br><br>Risk Ratio (95%CI)
Any prior pregnancy ended in SB	Prior pregnancy but no SB	1.00 [1.00,1.00]			
	Prior SB	1.08 [0.94,1.23]			
	No prior pregnancy	1.08** [1.02,1.15]			
Any prior pregnancy ended in miscarriage	Prior pregnancy but no miscarriage	1.00 [1.00,1.00]			
	Prior miscarriage	0.94 [0.86,1.03]			
	No prior pregnancy	1.07* [1.01,1.13]			
Any prior pregnancy ended in multiples	Prior pregnancy but no multiples	1.00 [1.00,1.00]			
	Prior multiples	1.14 [0.87,1.49]			
	No prior pregnancy	1.08** [1.02,1.14]			
Number of ANC Visits	No visit	1.00 [1.00,1.00]		1.00 [1.00,1.00]	1.00 [1.00,1.00]
	1 visit	0.98 [0.89,1.07]		1.00 [0.90,1.12]	1.00 [0.90,1.12]
	2-3 visit	0.92* [0.85,0.99]		0.94 [0.86,1.03]	0.93 [0.85,1.02]
	4 or more	0.54*** [0.50,0.59]		0.64*** [0.57,0.71]	0.62*** [0.56,0.70]
Place of Delivery	Home/Maiti	1.00 [1.00,1.00]			1.00 [1.00,1.00]
	HP/Clinic/Hospital	0.84*** [0.79,0.89]			1.04 [0.96,1.12]
	Way to Facility/Outdoors	1.28** [1.09,1.51]			1.23 [1.00,1.52]
Multiple Birth	Singleton	1.00 [1.00,1.00]	1.00 [1.00,1.00]	1.00 [1.00,1.00]	1.00 [1.00,1.00]
	Twin/Triplet	3.92*** [3.52,4.38]	4.91*** [4.20,5.75]	4.97*** [4.25,5.82]	4.96*** [4.24,5.81]
Sex of the Child	Female	1.00 [1.00,1.00]	1.00 [1.00,1.00]	1.00 [1.00,1.00]	1.00 [1.00,1.00]
	Male	1.10*** [1.04,1.17]	1.10** [1.02,1.17]	1.08* [1.01,1.16]	1.08* [1.01,1.16]
	Twin/Triplet	4.13*** [3.69,4.63]	1.00 [1.00,1.00]	1.00 [1.00,1.00]	1.00 [1.00,1.00]
STI in at least one visit of 2nd trimester?	No	1.00 [1.00,1.00]			
	Yes	0.99 [0.92,1.07]			
STI in at least one visit of 3rd trimester?	No	1.00 [1.00,1.00]			
	Yes	1.01 [0.92,1.12]			
Respiratory Problem in at least one visit of 2nd trimester?	No	1.00 [1.00,1.00]	1.00 [1.00,1.00]	1.00 [1.00,1.00]	1.00 [1.00,1.00]
	Yes	1.00 [0.94,1.06]	1.08 [1.00,1.16]	1.09* [1.01,1.18]	1.09* [1.01,1.18]
Respiratory Problem in at least one visit of 3rd trimester?	No	1.00 [1.00,1.00]	1.00 [1.00,1.00]	1.00 [1.00,1.00]	1.00 [1.00,1.00]
	Yes	0.85*** [0.79,0.92]	0.86** [0.79,0.94]	0.86** [0.78,0.94]	0.86** [0.78,0.94]
GI Problem in at least one visit of 2nd trimester?	No	1.00 [1.00,1.00]			
	Yes	1.08 [0.98,1.18]			
GI Problem in at least one visit of 3rd trimester?	No	1.00 [1.00,1.00]			
	Yes	1.04 [0.94,1.16]			

**Name of Variables**	**Categories**	**Model 1 Unadjusted Model**<br><br>Risk Ratio (95%CI)	**Model 2 Adjusted Model without ANC/Place of Delivery (N=21,297)**<br><br>Risk Ratio (95%CI)	**Model 3 Adjusted- Added ANC**<br><br>**(N=19,965)**<br><br>Risk Ratio (95%CI)	**Model 4 Adjusted- Added ANC and Place of Delivery**<br><br>**(N=19,964)**<br><br>Risk Ratio (95%CI)
Poor appetite, nausea & vomiting in at least one visit of 2nd trimester?	No	1.00 [1.00,1.00]	1.00 [1.00,1.00]	1.00 [1.00,1.00]	1.00 [1.00,1.00]
	Yes	0.81^{***} [0.77,0.86]	0.86^{***} [0.80,0.92]	0.88^{***} [0.82,0.94]	0.88^{***} [0.81,0.94]
Poor appetite, nausea & vomiting in at least one visit of 3rd trimester?	No	1.00 [1.00,1.00]	1.00 [1.00,1.00]	1.00 [1.00,1.00]	1.00 [1.00,1.00]
	Yes	0.88^{**} [0.82,0.95]	0.86^{***} [0.79,0.94]	0.87^{**} [0.79,0.95]	0.87^{**} [0.79,0.95]
Vaginal Bleeding in at least one visit of 2nd trimester?	No	1.00 [1.00,1.00]	1.00 [1.00,1.00]	1.00 [1.00,1.00]	1.00 [1.00,1.00]
	Yes	0.91 [0.71,1.17]	0.84 [0.62,1.16]	0.83 [0.60,1.14]	0.83 [0.60,1.15]
Vaginal Bleeding in at least one visit of 3rd trimester?	No	1.00 [1.00,1.00]	1.00 [1.00,1.00]	1.00 [1.00,1.00]	1.00 [1.00,1.00]
	Yes	1.44^{*} [1.05,1.98]	1.53^{*} [1.08,2.18]	1.49^{*} [1.04,2.13]	1.50^{*} [1.04,2.15]
Swelling in at least one visit of 2nd trimester?	No	1.00 [1.00,1.00]	1.00 [1.00,1.00]	1.00 [1.00,1.00]	1.00 [1.00,1.00]
	Yes	1.32^{***} [1.12,1.55]	1.19 [0.98,1.46]	1.21 [0.98,1.48]	1.21 [0.99,1.48]
Swelling in at least one visit of 3rd trimester?	No	1.00 [1.00,1.00]	1.00 [1.00,1.00]	1.00 [1.00,1.00]	1.00 [1.00,1.00]
	Yes	1.25^{**} [1.09,1.44]	1.37^{***} [1.17,1.60]	1.36^{***} [1.15,1.60]	1.36^{***} [1.15,1.60]
High Systolic BP in one visit of 2nd trimester?	Normal Systolic BP	1.00 [1.00,1.00]	1.00 [1.00,1.00]	1.00 [1.00,1.00]	1.00 [1.00,1.00]
	High Systolic BP	0.89 [0.59,1.34]	0.67 [0.40,1.12]	0.65 [0.39,1.09]	0.65 [0.39,1.09]
High Systolic BP in one visit of 3rd trimester?	Normal Systolic BP	1.00 [1.00,1.00]	1.00 [1.00,1.00]	1.00 [1.00,1.00]	1.00 [1.00,1.00]
	High Systolic BP	1.92^{***} [1.52,2.41]	1.47^{*} [1.08,2.01]	1.49^{*} [1.08,2.07]	1.49^{*} [1.07,2.07]
High Diastolic BP in one visit of 2nd trimester?	Normal diastolic BP	1.00 [1.00,1.00]	1.00 [1.00,1.00]	1.00 [1.00,1.00]	1.00 [1.00,1.00]
	High diastolic BP	1.34^{**} [1.12,1.60]	1.09 [0.85,1.40]	1.06 [0.82,1.37]	1.06 [0.82,1.38]
High Diastolic BP in one visit of 3rd trimester?	Normal diastolic BP	1.00 [1.00,1.00]	1.00 [1.00,1.00]	1.00 [1.00,1.00]	1.00 [1.00,1.00]
	High diastolic BP	1.57^{***} [1.37,1.80]	1.41^{***} [1.17,1.70]	1.35^{**} [1.12,1.64]	1.35^{**} [1.12,1.64]
Average weight in 3rd trimester minus Average weight in 2nd trimester (kg)		0.88^{***} [0.87,0.90]	0.89^{***} [0.87,0.90]	0.89^{***} [0.87,0.90]	0.89^{***} [0.87,0.90]

* p < 0.05, ** p < 0.01, *** p < 0.001
