# Supplementary material for: Prevalence and predictors of spontaneous preterm births in Nepal: findings from a prospective, population-based pregnancy cohort in rural Nepal–a secondary data analysis
Source: BMJ Open. 2022 Dec 1;12(12):e066934. doi: 10.1136/bmjopen-2022-066934 (PMC9716942; doi:10.1136/bmjopen-2022-066934)
Supplement: Author's manuscript [file bmjopen-2022-066934.draft_revisions.pdf]

BMJ Open is committed to open peer review. As part of this commitment we make the peer review history of every article we publish publicly available.

When an article is published we post the peer reviewers' comments and the authors' responses online. We also post the versions of the paper that were used during peer review. These are the versions that the peer review comments apply to.

The versions of the paper that follow are the versions that were submitted during the peer review process. They are not the versions of record or the final published versions. They should not be cited or distributed as the published version of this manuscript.

BMJ Open is an open access journal and the full, final, typeset and author-corrected version of record of the manuscript is available on our site with no access controls, subscription charges or pay-per-view fees (http://bmjopen.bmj.com).

If you have any questions on BMJ Open's open peer review process please email info.bmjopen@bmj.com

BMJ Open

Prevalence and Predictors of Preterm Births in Nepal- Findings from a prospective, population-based pregnancy cohort in rural Nepal: A secondary data analysis

Journal:	*BMJ Open*
Manuscript ID	bmjopen-2022-066934
Article Type:	Original research
Date Submitted by the Author:	26-Jul-2022
Complete List of Authors:	Subedi, Seema; Johns Hopkins University Bloomberg School of Public Health, Department of International Health<br>Hazel , Elizabeth; Johns Hopkins University Bloomberg School of Public Health, Department of International Health<br>Mohan, Diwakar; Johns Hopkins University Bloomberg School of Public Health, Department of International Health<br>Zeger, Scott; Johns Hopkins University Bloomberg School of Public Health, Department of Biostatistics<br>Mullany, Luke C.; Johns Hopkins University Bloomberg School of Public Health, Department of International Health<br>Tielsch, James; George Washington University School of Public Health and Health Services, Department of Global Health<br>Khatry, Subarna; Nepal Nutrition Intervention Project<br>LeClerq, Steven C.; Johns Hopkins University Bloomberg School of Public Health, Department of International Health; Nepal Nutrition Intervention Project<br>Black, Robert E.; Johns Hopkins University Bloomberg School of Public Health, Department of International Health<br>Katz, Joanne; Johns Hopkins University Bloomberg School of Public Health, Department of International Health
Keywords:	OBSTETRICS, Public health < INFECTIOUS DISEASES, Epidemiology < INFECTIOUS DISEASES

SCHOLARONE™ Manuscripts

I, the Submitting Author has the right to grant and does grant on behalf of all authors of the Work (as defined in the below author licence), an exclusive licence and/or a non-exclusive licence for contributions from authors who are: i) UK Crown employees; ii) where BMJ has agreed a CC-BY licence shall apply, and/or iii) in accordance with the terms applicable for US Federal Government officers or employees acting as part of their official duties; on a worldwide, perpetual, irrevocable, royalty-free basis to BMJ Publishing Group Ltd ("BMJ") its licensees and where the relevant Journal is co-owned by BMJ to the co-owners of the Journal, to publish the Work in this journal and any other BMJ products and to exploit all rights, as set out in our licence.

The Submitting Author accepts and understands that any supply made under these terms is made by BMJ to the Submitting Author unless you are acting as an employee on behalf of your employer or a postgraduate student of an affiliated institution which is paying any applicable article publishing charge ("APC") for Open Access articles. Where the Submitting Author wishes to make the Work available on an Open Access basis (and intends to pay the relevant APC), the terms of reuse of such Open Access shall be governed by a Creative Commons licence – details of these licences and which Creative Commons licence will apply to this Work are set out in our licence referred to above.

Other than as permitted in any relevant BMJ Author's Self Archiving Policies, I confirm this Work has not been accepted for publication elsewhere, is not being considered for publication elsewhere and does not duplicate material already published. I confirm all authors consent to publication of this Work and authorise the granting of this licence.

For peer review only

Prevalence and Predictors of Preterm Births in Nepal- Findings from a prospective, population-based pregnancy cohort in rural Nepal: A secondary data analysis

Authors: Seema Subedi[1], Elizabeth Hazel[1] Diwakar Mohan[1] Scott Zeger[2], Luke C. Mullany[1], James M. Tielsch[3], Subarna K. Khatry[4], Steven C. LeClerq[1,4], Robert E. Black[1], Joanne Katz[1]

Affiliations:

[1] Department of International Health, Johns Hopkins Bloomberg School of Public Health, 615 N. Wolfe Street, Baltimore, MD, 21205, USA.

[2] Department of Biostatistics, Johns Hopkins Bloomberg School of Public Health, 615 N. Wolfe Street, Baltimore, MD, 21205, USA.

[3] Department of Global Health, Milken Institute School of Public Health, George Washington University, 950 New Hampshire Avenue, Washington, DC, USA

[4] Nepal Nutrition Intervention Project – Sarlahi, Krishna Galli, Lalitpur, Kathmandu, Nepal.

Emails: ssubedi2@jhu.edu, ehazel1@jhu.edu, dmohan3@jhu.edu, sz@jhu.edu, lmullany@gmail.com, jtielsch@email.gwu.edu, skhatry@wlink.com.np, steveleclerq@gmail.com, rblack1@jhu.edu, mailto:jkatz1@jhu.edu

Correspondence: Seema Subedi, MPH. Department of International Health, Johns Hopkins Bloomberg School of Public Health, 615 N. Wolfe Street, Baltimore, MD, 21205, United States.

ssubedi2@jhu.edu

Word Count:

Abstract: 297 words

Main text (including tables):6093 words

Main text (excluding tables):(6093-1877=4216) words

For peer review only

For peer review only

For peer review only - http://bmjopen.bmj.com/site/about/guidelines.xhtml

ABSTRACT

Objective
Preterm birth can have short and long-term complications for a child. Baseline socioeconomic factors and pregnancy-related signs and symptoms may be important to predict and prevent preterm births in low-resource settings. The objective of our study was to find prevalence and predictors of preterm birth in rural Nepal.

Design
This is a secondary observational analysis of data.

Setting
Rural Sarlahi district, Nepal

Participants
40,119 pregnant women enrolled from September 9, 2010, to Jan 16, 2017

Outcome Measures
The outcome variable is preterm birth. Generalized Estimating Equations (GEE) Poisson regression with robust variance was fitted to present effect estimates as risk ratios.

Result
The prevalence of preterm birth was 15% (95% CI: 14.6%, 15.4%). Baseline characteristics associated with increased risk of preterm birth were maternal age less than 18 (ARR=1.13, 95% CI: 1.02-1.26); being Muslim (1.53, 1.16-2.01); first pregnancy (1.15, 1.04-1.28); multiple birth (4.91, 4.20-5.75) and male child (1.10, 1.02-1.17). Those associated with decreased risk were maternal education of more than 5 years (0.81, 0.73-0.90); and being from wealthier families (0.83, 0.74-0.93). Pregnancy related signs and symptoms associated with increased risk of preterm birth were vaginal bleeding (1.53, 1.08-2.18); swelling (1.37, 1.17-1.60); high systolic BP (1.47, 1.08-2.01) and high diastolic BP (1.41, 1.17-1.70) in the 3rd trimester. Those associated with decreased risk of preterm birth were respiratory problem in the 3rd trimester (0.86, 0.79-0.94); and having poor appetite, nausea and vomiting in the 2nd trimester (0.86, 0.80-0.92) and in the 3rd trimester (0.86, 0.79-0.94); and higher weight gain from the 2nd to 3rd trimester (0.89, 0.87-0.90).

Conclusion
The prevalence of preterm is high in rural Nepal. Interventions that increase maternal education may play role. Monitoring morbidity during antenatal care to intervene to reduce them through an effective health system may help reduce preterm.

Trial Registration Number
NCT01177111

For peer review only

STRENGTHS AND LIMITATIONS OF THIS STUDY

- Previous studies on preterm birth in Nepal were hospital-based, enrolled women during delivery and have explored only the women's baseline socio-demographic factors associated with preterm birth. Our study is population-based, enrolls women from earlier days of pregnancy, and have explored symptoms and morbidity variables that change throughout pregnancy. Women were followed at monthly intervals to reduce recall bias about pregnancy symptoms and morbidities.

- Gestational age (GA) at outcome has been measured using date of last menstrual period (LMP), and not the standard ultrasound (USG) method, however, as LMP was asked at enrollment which was generally early in pregnancy, there is less recall bias than LMP recalled at delivery or late in pregnancy. In addition, the pregnancy surveillance, that asked women if they had their period in the past 5 weeks and administered a pregnancy test if not, have improved women's recall of date of LMP at the time of enrollment.

For peer review only - http://bmjopen.bmj.com/site/about/guidelines.xhtml

INTRODUCTION:

Preterm birth (PTB), is defined as a birth occurring before 37 completed gestational weeks or fewer than 259 days from a woman's last menstrual period (LMP).[1] In 2010, the global prevalence of preterm birth estimated in 92 countries was 11.1% (95% CI: 9.1%-13.4%), ranging from about 5% in some European countries to 18% in some African countries.[2] Sixty percent of these PTBs occurred in Sub-Saharan Africa and South Asia.[2] Complications of preterm birth was the leading cause of under-5 mortality and accounted for approximately 17.7 % of all under-5 mortality and 36.1% of neonatal mortality, according to the 2019 global estimates. [3] Eighty-one percent of the under-5 deaths from complications of preterm birth occurred in Asia and sub-Saharan Africa countries.[4]

Preterm births can have short and long-term consequences. Short-term consequences comprise increased risks of neonatal respiratory conditions, sepsis, neurological conditions, feeding difficulties, and visual and hearing problems.[5-7] As the child grows, long term consequences include more hospital admissions, poorer neurodevelopment outcomes, difficulties in learning, as well as behavioral and social-emotional problems.[8-10] At the family level, preterm birth can lead to significant economic and psychological difficulties, and at the national level, it leads to significant cost for the health system.[11, 12]

In Nepal, under-five mortality has dropped from 64 deaths to 39 deaths per 1000 live births (LB) from 2001 to 2016.[13-15] In the same period, neonatal mortality has also steadily declined, (from 39 to 21 per 1,000 LB). [13-15]. Being an important determinant of neonatal mortality, preterm birth has become a greater contributor to under-5 mortality over time.[16] If we do not consider interventions to address preterm births, it would be difficult to achieve Nepal's Sustainable Development Goal (SDG) that aims to reduce the neonatal mortality to 12 per 1000 LB and under-5 mortality to 28 per 1000 LB by 2030.[17]

There are very few studies on the prevalence or risk factors for preterm birth in Nepal,[18, 19] and those that exist have limitations. First, most studies are hospital based. Women enrolled in hospitals during delivery may suffer from systematic recall bias, where women having a preterm birth might report differently from women with term births. Also, at the time of delivery, women might have recall issues in reporting their date of last menstrual period (LMP). Most important, enrolling at facilities has a selection bias, where the preterm births delivered

For peer review only - http://bmjopen.bmj.com/site/about/guidelines.xhtml

at home or on-the-way to facilities are missed, possibly leading to underestimation of the prevalence and a different distribution of risk factors. Second, previous studies have included deliveries taken from urban tertiary hospitals in Nepal. Around 80% of the Nepalese population resides in rural areas[20] and do not have access to delivery services at tertiary centers. So, the findings from those studies may not be representative of rural Nepal. Third, since the women's enrollment was during delivery, they looked at only baseline risk factors and did not analyze changing symptoms and maternal weight gain throughout pregnancy. Some of these symptoms may be indicative of conditions that can be addressed by antenatal care . The objective of our study was to estimate the prevalence and identify predictors/risk factors of preterm births in rural Nepal. Understanding and addressing such risk factors is critical to addressing neonatal and child mortality and morbidity, particularly in resource-poor settings like Nepal.

METHODS:

Study Design

This is a secondary data analysis with data taken from the Nepal Oil Massage Study (NOMS), which is a cluster-randomized community-based trial (ClinicalTrials.gov, NCT01177111) on the impact of sunflower seed oil versus standard of care mustard seed oil massage on neonatal mortality and morbidity in rural Sarlahi district of Nepal. This study began by identifying married women of childbearing age (15 to 40 years) who consented to pregnancy surveillance. This involved following them every 5 weeks to see whether they became pregnant, based on a positive pregnancy test offered by the study team if a woman reported missing a period. If pregnant, they were consented and enrolled in the trial. During enrollment, demographic data, socioeconomic status, reproductive history, and date of last menstruation were collected. Then they were visited monthly by a field worker until the pregnancy outcome occurred. During these monthly visits, field workers asked some basic questions about signs and symptoms of morbidity during the previous 30-day period. At these visits, women also had their weight and blood pressure (BP)/pulse measured, and body temperature recorded. Women reporting signs of morbidity and indicating that these signs were currently present were referred to the local health post or Primary Health Center. Women with fever or elevated blood pressure as measured by study staff were similarly referred.

As soon as possible after labor began or the baby was delivered, family members or neighbors notified the local female study worker of the birth. She notified a specially trained team who

For peer review only - http://bmjopen.bmj.com/site/about/guidelines.xhtml

For peer review only

visited the mother and infant as soon after birth as possible. They measured infant weight and time of weight measurement after birth, determined sex of the newborn and whether the baby was a singleton or multiple birth.

Setting and Participants

The study cohort consists of 40,119 pregnancies among married women of child-bearing age, living in 34 VDCs of Sarlahi district, enrolled from September 9, 2010, to Jan 16, 2017, in the NOMS study. Pregnancies were followed monthly until delivery. Live births were categorized as term or preterm. Pregnancies ending in miscarriage, abortion and stillbirths (SB) were excluded from the analysis. Stillbirths were not included because the etiology of these may be quite different from those of preterm births.

Variables

<u>Outcome Variable</u>

The main outcome variable is preterm birth among pregnancies that produced at least one live born infant, defined as pregnancies ending less than 259 gestational days from the first day of LMP date. Live births were based on women's self- report. They were asked if the baby moved, cried or breathed after birth. If they said "yes" to one or more of these, the birth was recorded as a live birth. For gestational age (GA), women were asked about their LMP during enrollment, and the GA at outcome was calculated as the difference between reported LMP and the date of the child's birth.

<u>Independent Variables</u>

Through literature review and expert opinion, certain factors were included in the analysis of predictors. [21] These can be categorized into baseline and pregnancy-varying variables. Baseline variables included sociodemographic, prior pregnancy related, current pregnancy related and child related variables. Pregnancy-varying variables included signs and symptoms of morbidity in pregnancy, and maternal weight.

Sociodemographic variables like maternal age, caste/religion, maternal education, wealth quintile and maternal height were explored. Maternal age was categorized as less than 18, 18-35 and more than 35 years to assess the association of very young women and older women with preterm births. Caste/religion of the mothers (Brahmin /Chhetri, Vaishya, Shudra, Muslim

For peer review only - http://bmjopen.bmj.com/site/about/guidelines.xhtml

For peer review only

and others) were used as per the caste category system in Nepal. [22] Maternal education (No schooling, 1-5 years and more than 5 years); and maternal height (<145 cm, 145-<150 and ≥150) were used. Household wealth status was measured in quintiles based on a standardized score using principal components analysis of household assets. [23]

Prior pregnancy related variables like parity (no prior pregnancy, prior pregnancy that did not result in a live or stillbirth, 1-4, and more than 4 prior pregnancies resulting in a live or stillbirth; interpregnancy interval (IPI) defined as the time since the end of last pregnancy to the date of LMP of the current pregnancy, regardless of the outcome (<18, 18-36, and >36 months); any prior live born child who died (Yes and No); any prior pregnancy that ended in a SB (Yes and No); any prior pregnancy that ended in multiples (Yes and No); and any prior pregnancy ending in miscarriage (Yes and No) were assessed.

Current pregnancy related variables like tobacco intake (ever used any tobacco products during this pregnancy- Yes and No), and alcohol intake (ever used alcohol during this pregnancy- Yes and No) were assessed. Child-level variables like multiple birth (singleton and twin/triplet), and sex of the child (male and female) were included. Current pregnancy related variables like tobacco and alcohol intake were not included in the regressions because rates of use were very low. Only 0.3% consumed alcohol and only 1.1% used tobacco. Other current pregnancy related variables like number of antenatal care (ANC) visits and place of delivery were shown in descriptive, but omitted from inferential analysis because in this setting, women with preterm births could have missed the 4th ANC visit in the 9th month and preterm birth could be the cause of a lower number of visits. For place of delivery, preterm births were more likely to be delivered at home or on the way to the facility, because many births in this environment are not planned to occur in a facility.

Symptoms of morbidity during pregnancy such as sexually transmitted diseases (STI), respiratory illness, gastrointestinal (GI) illness, poor appetite, nausea and vomiting, vaginal bleeding, swelling of hands or face, high systolic and diastolic blood pressure were assessed. All these variables were assessed in the 2nd and 3rd trimester, and so labelled as – Problem in at least one visit of the 2nd trimester- Yes or No, and Problem in at least one visit in the 3rd trimester- Yes or No. We did not include symptoms in the 1st trimester because only 41% women were enrolled in the 1st trimester, and so 59% missed symptom information in the 1st

For peer review only

trimester. Maternal weight gain was defined as the average weight in the 3rd trimester minus the average weight in the 2nd trimester. For measurement of these symptom variables, field workers asked if women had symptoms of morbidity at any time in the past 30 days, at each monthly visit during pregnancy. STI was defined as painful or burning urination, or foul smelling vaginal discharge. Respiratory illness was defined as persistent cough or difficult or rapid breathing, or wheezing/grunting, or shortness of breath. GI illness was defined as watery stools (4 or more times in a day or blood or white mucus in the stool). Appetite related illness was defined as poor appetite, nausea or vomiting. Vaginal bleeding was defined as spots of blood from the vagina. Swelling was defined as swelling of hands and/or face. Foot/leg swelling was excluded since it is common during pregnancy and not indicative of underlying disease. BP measurements were categorized as high systolic BP if the systolic measurement was >=140 mmHg, and high diastolic BP if diastolic measurement was >=90 mmHg at any monthly visit within the 2nd or 3rd trimester.

Preterm births were classified as spontaneous or not (caesarian section or induction), and only spontaneous preterm births were included in analysis.

Statistical Methods

First, a descriptive analysis was done to show the frequencies of baseline variables (socio demographic, prior pregnancy related, current pregnancy and child related) and pregnancy varying variables (symptoms and maternal weight) by preterm and term births. Second, bivariable GEE Poisson regression with robust variance was used to examine associations between each risk factor and the outcome to get an unadjusted risk ratio. Since the prevalence of our outcome was more than 10%, we used Poisson regression with robust variance because we wanted to report associations as risk ratios. Third, multivariable GEE Poisson regression with robust variance was used including variables that were significant in the bivariable models, to get the adjusted risk ratios (ARR). Correlation between the different infants from the same mother was considered as exchangeable in the GEE model.

Among the 31,880 pregnancies that ended in a live birth, 29 pregnancies had missing gestational age at outcome. We excluded them from analysis, and so the descriptive analysis had 31,851 pregnancies. In the regression analysis, we excluded the pregnancies (3.4%) that ended in caesarian section, induction or both. Pregnancies with missing symptom variables

For peer review only - http://bmjopen.bmj.com/site/about/guidelines.xhtml

were further excluded. 59%, 20% and 9% pregnancies had symptom variables missing in the 1st, 2nd and 3rd trimesters, respectively. We excluded first trimester data because data were missing for more than 50% pregnancies (women were not enrolled until after the 1st trimester). Since there was missing information for 29% of symptoms in 2nd and/or 3rd trimester, the final multivariable regression omitted these pregnancies, and so regression analysis consisted of 21,297 pregnancies.

Patient and Public Involvement

Patients or the public were not involved in the design, or conduct, or reporting, or dissemination plans of this study.

RESULTS:

Participants

The analytic population is 31,851 pregnancies that ended in at least one live birth and had information on gestational age at outcome. The detailed flow chart is given in Figure 1. Most women were enrolled in the 1st and 2nd trimester (41% each), followed by the 3rd trimester (18%). Overall, the mean gestational age at enrollment was 18 weeks. For 1st, 2nd and 3rd trimesters, the mean GA at enrollment were 9, 19 and 34 weeks respectively.

Descriptive Analysis

For baseline variables, as seen in Table 1, 15% of women were younger (less than 18) and 2% women were older (more than 35 years of age). 9% of women were Muslim caste/religion. Two thirds of women did not go to school, whereas only nearly one fourth had an education of more than five years. 15% of women had height <145cm. About a third (29%) of women had their first pregnancy in this study and 64% had one to four prior live or still births. Among those who had a previous pregnancy, 6% had prior still birth, 16% experienced miscarriage and 16% had a live birth that died, and only 1% had prior multiples. Half the women had an interpregnancy interval of less than 18 months, and 28% of women had four or more ANC visits. Half of the babies were born at home and 2% were born on the way to a facility or outdoors. Only 1.1% consumed tobacco and only 0.3% consumed alcohol during pregnancy. Half of the current pregnancies (51%) resulted in male children, and less than 1% resulted in

For peer review only - http://bmjopen.bmj.com/site/about/guidelines.xhtml

multiple births. Only 3.4% of pregnancies underwent either caesarian section or induction or both.

For pregnancy-varying variables, as seen in Table 2, poor appetite, nausea and vomiting was the most commonly reported symptom in both the second (39%) and third trimesters (20%); and vaginal bleeding was the least reported symptom (1.2% in the second and 0.6% in the third trimester). Very few women had high systolic blood pressure (0.5% and 0.8%) and high diastolic blood pressure (1.5% and 2.9%) in second and third trimesters respectively. The average weight gained by women from second to third trimester was 3.5 kg.

For peer review only

Table 1: Distribution of Baseline Variables by preterm and term births

Variables		Total N=31,851	Term N=27,059	Preterm N=4,792
		N (%)	N (%)	N (%)
Maternal Age	18 to 35	26,206 (82.3)	22,423 (82.9)	3,783 (78.9)
	Less than 18	4,946 (15.5)	4,100 (15.2)	846 (17.7)
	More than 35	699 2.2)	536 2.0)	163 (3.4)
Caste/Religion	Brahmin and Chhetri	963 (3.0)	857 (3.2)	106 (2.2)
	Vaishya	22,946 (72.0)	19,701 (72.8)	3,245 (67.7)
	Shudra	4,922 (15.5)	4,111 (15.2)	811 (16.9)
	Muslim and others	2,989 (9.4)	2,365 (8.7)	624 (13.0)
	Missing	31 (0.1)	25 (0.1)	6 0.1)
	Missing	21 (0.1)	16 (0.1)	5 (0.1)
Maternal Education	No schooling	21,427 (67.3)	17,915 (66.2)	3,512 (73.3)
	1 to 5 years	2,713 (8.5)	2,330 (8.6)	383 (8.0)
	More than 5 years	7,681 (24.1)	6,786 (25.1)	895 (18.7)
	Missing	30 (0.1)	28 (0.1)	2 (0.0)
Quintiles of Wealth	Poorest	6,510 (20.4)	5,340 (19.7)	1,170 (24.4)
	Poor	6,380 (20.0)	5,403 (20.0)	977 (20.4)
	Middle	6,320 (19.8)	5,314 (19.6)	1,006 (21.0)
	Richer	6,296 (19.8)	5,470 (20.2)	826 (17.2)
	Richest	6,324 (19.9)	5,516 (20.4)	808 (16.9)
	Missing	21 (0.1)	16 (0.1)	5 (0.1)
Maternal Height (cms)	<145	4,689 (14.7)	3,885 (14.4)	800 (16.7)
	145-<150	9,559 (29.9)	8,025 (29.7)	1,527 (31.9)
	>=150	17,581 (55.1)	15,111 (55.8)	2,454 (51.2)
	Missing	51 (0.2)	38 (0.14)	11 (0.2)
Parity including both LB and SB, at Enrollment	Parity 1 to 4	20,317 (63.8)	17,366 (64.2)	2,951 (61.6)
	More than 4	1,383 (4.3)	1,117 (4.1)	266 (5.6)
	Prior Pregnant but parity 0	787 (2.5)	672 (2.5)	115 (2.4)
	No Prior Pregnant	9,195 (28.9)	7,769 (28.7)	1,426 (29.8)
	Missing	169 (0.5)	135 (0.5)	34 (0.7)
Interpregnancy Interval based on maternal recall	18 to 36 months	7,927 (24.9)	6,787 (25.1)	1,140 (23.8)
	Less than 18 months	11,461 (36.0)	9,701 (35.9)	1,760 (36.7)
	More than 36 months	3,256 (10.2)	2,794 (10.3)	462 (9.6)
	No Prior Pregnancy	9,195 (28.9)	7,769 (28.7)	1,426 (29.8)
	Missing	12 (0.0)	8 (0.0)	4 (0.1)
Any deaths among Prior LB	Prior LB but not died	17,488 (54.9)	14,999 (55.4)	2,489 (51.9)
	Prior LB died	3,618 (11.4)	2,999 (11.1)	619 (12.9)
	Prior Pregnancy but no LB	1,073 (3.4)	909 (3.4)	164 (3.4)
	No prior pregnancy	9,195 (28.9)	7,769 (28.7)	1,426 (29.8)
	Missing	477 (1.5)	383 (1.4)	94 (2.0)
Any prior pregnancy ended in SB	Prior Pregnancy but no SB	21,270 (66.8)	18,127 (67.0)	3,143 (65.6)
	Prior SB	1,371 (4.3)	1,150 (4.2)	221 (4.6)

For peer review only

Variables		Total N=31,851	Term N=27,059	Preterm N=4,792
		N (%)	N (%)	N (%)
	No prior pregnancy	9,195 (28.9)	7,769 (28.7)	1,426 (29.8)
	Missing	15 (0.0)	13 (0.0)	2 (0.0)
Any prior pregnancy ended in miscarriage	Prior Pregnancy but no miscarriage	19,025 (59.7)	16,176 (59.8)	2,849 (59.5)
	Prior miscarriage	3,621 (11.4)	3,104 (11.5)	517 (10.8)
	No prior pregnancy	9,195 (28.9)	7,769 (28.7)	1,426 (29.8)
	Missing	10 (0.0)	10 (0.0)	0 (0.0)
Any prior pregnancy ended in multiples	Prior Pregnancy but no multiples	22,343 (70.1)	19,030 (70.3)	3,313 (69.1)
	Prior multiples	292 (0.9)	241 (0.9)	51 (1.1)
	No prior pregnancy	9,195 (28.9)	7,769 (28.7)	1,426 (29.8)
	Missing	21 (0.1)	19 (0.1)	2 (0.0)
Number of ANC visits	No visit	5,520 (17.3)	4,524 (16.7)	996 (20.8)
	1 visit	4,146 (13.0)	3,420 (12.6)	726 (15.2)
	2-3 visit	9,779 (30.7)	8,158 (30.1)	1,621 (33.8)
	4 or more	8,909 (28.0)	8,021 (29.6)	888 (18.5)
	Missing	3,497 (11.0)	2,936 (10.9)	561 (11.7)
Place of Delivery	Home/Maiti	15,776 (49.5)	13,270 (49.0)	2,506 (52.3)
	HP/Clinic/Hospital	12,016 (37.7)	10,406 (38.5)	1,610 (33.6)
	Way to Facility/Outdoors	610 (1.9)	486 (1.8)	124 (2.6)
	Missing	3,449 (10.8)	2,897 (10.7)	552 (11.5)
Bidi or tobacco use in pregnancy	No	31,498 (98.9)	26,789 (99.0)	4,709 (98.3)
	Yes	353 (1.1)	270 (1.0)	83 (1.7)
Alcohol use (jaard or rakshi) in pregnancy?	No	31,756 (99.7)	26,982 (99.7)	4,774 (99.6)
	Yes	95 (0.3)	77 (0.3)	18 (0.4)
Multiple Birth	Singleton	31,587 (99.2)	26,946 (99.6)	4,641 (96.8)
	Twin/Triplet	264 (0.8)	113 (0.4)	151 (3.2)
Sex of the child	Female	15,182 (47.7)	13,063 (48.3)	2,119 (44.2)
	Male	16,306 (51.2)	13,794 (51.0)	2,512 (52.4)
	Twin/Triplet	264 (0.8)	113 (0.4)	151 (3.2)
	Missing	99 (0.3)	89 (0.3)	10 (0.2)
Induction or CS done	Only Induction	193 (0.6)	166 (0.6)	27 (0.6)
	Only CS	868 (2.7)	735 (2.8)	133 (2.8)
	Both Induction and CS	32 (0.1)	28 (0.1)	4 (0.08)
	None	30,758 (96.6)	26130 (96.6)	4628 (96.6)

For peer review only

Table 2: Distribution of pregnancy-varying variables by preterm and term births

Variables		Total N=31,851	Term N=27,059	Preterm N=4,792
		N (%)	N (%)	N (%)
STI in at least one visit of 2nd trimester?	No	20,823 (65.4)	17,497 (64.7)	3,326 (69.4)
	Yes	4,593 (14.4)	3,855 (14.2)	738 (15.4)
	Missing	6,435 (20.2)	5,707 (21.1)	728 (15.2)
STI in at least one visit of 3rd trimester?	No	25,931 (81.4)	22,512 (83.2)	3,419 (71.3)
	Yes	2,963 (9.3)	2,569 (9.5)	394 (8.2)
	Missing	2,957 (9.3)	1,978 (7.3)	979 (20.4)
Respiratory Problems in at least one visit of 2nd trimester?	No	17,963 (56.4)	15,081 (55.7)	2,882 (60.1)
	Yes	7,452 (23.4)	6,271 (23.2)	1,181 (24.6)
	Missing	6,436 (20.2)	5,707 (21.1)	729 (15.2)
Respiratory Problems in at least one visit of 3rd trimester?	No	22,860 (71.8)	19,743 (73.0)	3,117 (65.0)
	Yes	6,034 (18.9)	5,338 (19.7)	696 (14.5)
	Missing	2,957 9.3)	1,978 (7.3)	979 (20.4)
GI Problems in at least one visit of 2nd trimester?	No	22,742 (71.4)	19,136 (70.7)	3,606 (75.3)
	Yes	2,673 (8.4)	2,216 (8.2)	457 (9.5)
	Missing	6,436 (20.2)	5,707 (21.1)	729 (15.2)
GI Problems in at least one visit of 3rd trimester?	No	26,152 (82.1)	22,712 (83.9)	3,440 (71.8)
	Yes	2,742 (8.6)	2,369 (8.8)	373 (7.8)
	Missing	2,957 (9.3)	1,978 (7.3)	979 (20.4)
Poor app, nausea & vomiting in at least one visit of 2nd trimester?	No	13,121 (41.2)	10,814 (40.0)	2,307 (48.1)
	Yes	12,295 (38.6)	10,538 (38.9)	1,757 (36.7)
	Missing	6,435 (20.2)	5,707 (21.1)	728 (15.2)
Poor appetite, nausea & vomiting in at least one visit of 3rd trimester?	No	22,486 (70.6)	19,437 (71.8)	3,049 (63.6)
	Yes	6,409 (20.1)	5,645 (20.9)	764 (15.9)
	Missing	2,956 (9.3)	1,977 (.3)	979 (20.4)
Vaginal Bleeding in at least one visit of 2nd trimester?	No	25,042 (78.6)	21,036 (77.7)	4,006 (83.6)
	Yes	373 (1.2)	315 (1.2)	58 (1.2)
	Missing	6,436 (20.2)	5,708 (21.1)	728 (15.2)
Vaginal Bleeding in at least one visit of 3rd trimester?	No	28,716 (90.2)	24,938 (92.2)	3,778 (78.8)
	Yes	178 (0.6)	143 (0.5)	35 (0.7)
	Missing	2,957 (9.3)	1,978 (7.3)	979 (20.4)
Swelling in at least one visit of 2nd trimester?	No	24,846 (78.0)	20,904 (77.3)	3,942 (82.3)
	Yes	571 (1.8)	448 (1.7)	123 (2.6)
	Missing	6,434 (20.2)	5,707 (21.1)	727 (15.2)

For peer review only
For peer review only - http://bmjopen.bmj.com/site/about/guidelines.xhtml

**Variables**		**Total N=31,851**	**Term N=27,059**	**Preterm N=4,792**
		**N (%)**	**N (%)**	**N (%)**
Swelling in at least one visit of 3rd trimester?	No	27,754 (87.1)	24,126 (89.2)	3,628 (75.7)
	Yes	1,141 (3.6)	956 (3.5)	185 (3.9)
	Missing	2,956 (9.3)	1,977 (7.3)	979 (20.4)
High Systolic BP in 2nd trimester?	Normal Systolic BP	25,260 (79.3)	21,217 (78.4)	4,043 (84.4)
	High Systolic BP	158 (0.5)	136 (0.5)	22 (0.5)
	Missing	6,433 (20.2)	5,706 (21.1)	727 (15.2)
High Systolic BP in 3rd trimester?	Normal Systolic BP	28,659 (90.0)	24,905 (92.0)	3,754 (78.3)
	High Systolic BP	241 (0.8)	181 (0.7)	60 (1.3)
	Missing	2,951 (9.3)	1,973 (7.3)	978 (20.4)
High diastolic BP in 2nd trimester?	Normal diastolic BP	24,945 (78.3)	20,976 (77.5)	3,969 (82.8)
	High diastolic BP	473 (1.5)	377 (1.4)	96 (2.0)
	Missing	6,433 (20.2)	5,706 (21.1)	727 (15.2)
High diastolic BP in 3rd trimester?	Normal diastolic BP	27,982 (87.9)	24,360 (90.0)	3,622 (75.6)
	High diastolic BP	918 (2.9)	726 (2.7)	192 (4.0)
	Missing	2,951 (9.3)	1,973 (7.3)	978 (20.4)
Average weight in 3rd trimester minus Average weight in 2nd trimester (Mean)		3.5	3.6	2.9

For peer review only - http://bmjopen.bmj.com/site/about/guidelines.xhtml

Outcome data

There were 4,792 preterm births out of 31,851 pregnancies with at least one LB. Hence, the prevalence of preterm birth was 15% (95% CI: 14.6%, 15.4%) among the pregnancies enrolled between September 9, 2010, to January 16, 2017. On looking at severity of preterm, the prevalence were 0.5%, 1.5% and 2.1% and 10.9% for extreme PTB (<28 weeks), very PTB (28-<32 weeks), moderate PTB (32-<34 weeks) and late PTB (34-<37 weeks) respectively.

Main results

The main results are shown in Table 3. Baseline variables that increased the risk of preterm were maternal age less than 18 (ARR=1.13, 95% CI: 1.02-1.26); being Muslim compared to Brahmin and Chhetri (1.53, 1.16-2.01); first pregnancy as compared to parity 1 to 4 (1.15, 1.04-1.28); having a multiple birth (4.91, 4.20-5.75) and having a male child (1.10, 1.02-1.17). Baseline variables that decreased the risk of preterm were maternal education of more than 5 years (0.81, 0.73-0.90); and being wealthier: richer (0.83, 0.74-0.93) wealth quintile compared to the poorest wealth quintile. Baseline variables that showed no association with preterm births in the bivariable/unadjusted models are any prior pregnancy ending in SB, any prior pregnancy ending in multiples, and any prior pregnancy ending in miscarriage, and interpregnancy interval. The baseline variable that showed an association in the bivariable model, but not in the multivariable models was any prior pregnancy ending in death for a live birth.

For morbidity symptoms, some increased the risk of preterm, and all of these showed increased risk when symptoms were present in the 3rd trimester. Having vaginal bleeding (ARR= 1.53, 95% CI: 1.08-2.18); swelling (1.37, 1.17-1.60); high systolic BP (1.47, 1.08-2.01) and high diastolic BP (1.41, 1.17-1.70) in the 3rd trimester significantly increased the risk of preterm. Some symptom variables significantly decreased the risk of preterm. Having respiratory problem in the 3rd trimester (0.86, 0.79-0.94); and having poor appetite, nausea and vomiting in the 2nd trimester (0.86, 0.80-0.92) and in the 3rd trimester (0.86, 0.79-0.94) decreased the risk of preterm. Symptom variables that showed no association with preterm were STI and GI problems. Symptom variables that were significant in the bivariable model, but not significant in the multivariable models were swelling in the 2nd trimester and diastolic blood pressure in the 2nd trimester. For maternal weight, higher weight gain from the 2nd to the 3rd trimester was associated with a decreased the risk of preterm (0.89, 0.87-0.90).

For peer review only - http://bmjopen.bmj.com/site/about/guidelines.xhtml

Table 3: Crude and Adjusted Risk Ratios for associations between risk factors and preterm birth

Name of Variables	Categories	Unadjusted Model	Adjusted Model (N=21,297)
		Risk Ratio (95% CI)	Risk Ratio (95% CI)
Maternal Age	18 to 35	1	1
	Less than 18	1.19*** [1.11,1.28]	1.13* [1.02,1.26]
	More than 35	1.57*** [1.36,1.81]	1.22 [0.98,1.51]
Caste/Religion Categories	Brahmin and Chhetri	1	1
	Vaishya	1.33** [1.09,1.62]	1.23 [0.95,1.59]
	Shudra	1.55*** [1.26,1.90]	1.23 [0.94,1.62]
	Muslim and others	1.96*** [1.60,2.42]	1.53** [1.16,2.01]
Mother's Years of Education	No schooling	1	1
	1 to 5 years	0.86** [0.78,0.95]	0.91 [0.80,1.03]
	More than 5 years	0.71*** [0.66,0.76]	0.81*** [0.73,0.90]
5 quintiles of Wealth	Poorest	1	1
	Poor	0.86*** [0.79,0.93]	0.90* [0.82,1.00]
	Middle	0.89** [0.82,0.96]	0.95 [0.86,1.05]
	Richer	0.73*** [0.67,0.79]	0.83** [0.74,0.93]
	Richest	0.71*** [0.65,0.77]	0.88* [0.78,1.00]
Mother's height(centimeter)	<145	1	1
	145-<150	0.93 [0.86,1.01]	0.98 [0.88,1.08]
	>=150	0.81*** [0.75,0.87]	0.89* [0.81,0.98]
Parity including both LB and SB, at Enrollment	Parity 1 to 4	1	1
	More than 4	1.32*** [1.17,1.48]	1.17 [0.99,1.37]
	Prior Pregnant but parity 0	1.02 [0.85,1.22]	0.92 [0.62,1.37]
	No Prior Pregnant	1.10** [1.04,1.17]	1.15** [1.04,1.28]
Interpregnancy Intervals	18 to 36 months	1	1
	Less than 18 months	1.07 [0.99,1.14]	1.08 [0.99,1.18]
	More than 36 months	0.98 [0.89,1.09]	0.9 [0.79,1.02]
	No Prior Pregnancy	1.11** [1.03,1.20]	1 [1.00,1.00]
Any prior pregnancy ended in death for LB?	Prior LB but not died	1	1
	Prior LB died	1.19*** [1.09,1.29]	1.07 [0.97,1.19]
	Prior Pregnancy but no LB	1.07 [0.92,1.25]	1.06 [0.75,1.49]
	No prior pregnancy	1.12*** [1.06,1.19]	1 [1.00,1.00]
Any prior pregnancy ended in SB?	Prior Pregnancy but no SB	1	
	Prior SB	1.08 [0.94,1.23]	
	No Prior Pregnancy	1.08** [1.02,1.15]	
Any prior pregnancy ended in miscarriage?	Prior Pregnancy but no miscarriage	1	
	Prior miscarriage	0.94 [0.86,1.03]	
	No Prior Pregnancy	1.07* [1.01,1.13]	
Any prior pregnancy ended in multiples?	Prior Pregnancy but no multiples	1	
	Prior multiples	1.14 [0.87,1.49]	
	No prior pregnancy	1.08** [1.02,1.14]	
Multiple Birth	Singleton	1	1
	Twin/Triplet	3.92*** [3.52,4.38]	4.91*** [4.20,5.75]
Sex of the child	Female	1	1
	Male	1.10*** [1.04,1.17]	1.10** [1.02,1.17]

For peer review only - http://bmjopen.bmj.com/site/about/guidelines.xhtml

Name of Variables	Categories	Unadjusted Model<br>Risk Ratio (95% CI)	Adjusted Model (N=21,297)<br>Risk Ratio (95% CI)
	Twin/Triplet	4.13^{***} [3.69,4.63]	1
STI in at least one visit of 2nd trimester?	No	1	
	Yes	0.99 [0.92,1.07]	
STI in at least one visit of 3rd trimester?	No	1	
	Yes	1.01 [0.92,1.12]	
Respiratory Problems in at least one visit of 2nd trimester?	No	1	1
	Yes	1 [0.94,1.06]	1.08 [1.00,1.16]
Respiratory Problems in at least one visit of 3rd trimester?	No	1	1
	Yes	0.85^{***} [0.79,0.92]	0.86^{**} [0.79,0.94]
GI Problems in at least one visit of 2nd trimester?	No	1	
	Yes	1.08 [0.98,1.18]	
GI Problems in at least one visit of 3rd trimester?	No	1	
	Yes	1.04 [0.94,1.16]	
Poor app, nausea & vomiting in at least one visit of 2nd trimester?	No	1	1
	Yes	0.81^{***} [0.77,0.86]	0.86^{***} [0.80,0.92]
Poor appetite, nausea & vomiting in at least one visit of 3rd trimester?	No	1	1
	Yes	0.88^{**} [0.82,0.95]	0.86^{***} [0.79,0.94]
Vaginal Bleeding in at least one visit of 2nd trimester?	No	1	1
	Yes	0.91 [0.71,1.17]	0.84 [0.71,1.17]
Vaginal Bleeding in at least one visit of 3rd trimester?	No	1	1
	Yes	1.44^{*} [1.05,1.98]	1.53^{*}[1.08,2.18]
Swelling in at least one visit of 2nd trimester?	No	1	1
	Yes	1.32^{***} [1.12,1.55]	1.19 [0.98,1.46]
Swelling in at least one visit of 3rd trimester?	No	1	1
	Yes	1.25^{**} [1.09,1.44]	1.37^{***} [1.17,1.60]
High Systolic BP in 2nd trimester?	Normal Systolic BP	1	1
	High Systolic BP	0.89 [0.59,1.34]	0.67 [0.40,1.12]
High Systolic BP in 3rd trimester?	Normal Systolic BP	1	1
	High Systolic BP	1.92^{***} [1.52,2.41]	1.47^{*} [1.08,2.01]
High diastolic BP in 2nd trimester?	Normal diastolic BP	1	1
	High diastolic BP	1.34^{**} [1.12,1.60]	1.09 [0.85,1.40]
High diastolic BP in 3rd trimester?	Normal diastolic BP	1	1
	High diastolic BP	1.57^{***} [1.37,1.80]	1.41^{***} [1.17,1.70]
Average weight in 3rd trimester minus Average weight in 2nd trimester		0.88^{***} [0.87,0.90]	0.89^{***} [0.87,0.90]

* $p < 0.05$, ** $p < 0.01$, *** $p < 0.001$

For peer review only - http://bmjopen.bmj.com/site/about/guidelines.xhtml

DISCUSSION

Our study is one of the only large-scale studies on preterm births using data from an existing pregnancy surveillance in rural Sarlahi, Nepal. The prevalence of preterm birth is 15%, higher than previous estimates from Nepal [18, 19] which were primarily from urban areas and large hospital-based studies. Our study's strength is that it was population-based and included all home and facility deliveries but is confined to a rural and relatively small geographic area (one third of a district). It should also be noted that health care seeking in pregnancy is low considering the low rates of antenatal care and facility deliveries. The low rates of induction and caesarean section point to a very low proportion of the PTBs being due to iatrogenic causes.

In many other settings, both younger and older maternal age has been reported to be risk factors for preterm birth. [24-30]. First pregnancy (primipara) has been shown to be associated with preterm birth in other studies. A study in France showed that primipara as compared to parity 2-3 increased the risk of preterm birth by 1.8 times.[31] Another study in the USA showed that being primipara as compared to multipara increased the risk of very preterm and extremely preterm birth, with the highest risk of 1.37 times for extremely preterm birth.[32] Primipara is a risk factor for hypertensive disorders of pregnancy (HDP), which increases the risk of preterm birth.[33] Our study did not show interpregnancy interval to be the risk factor for preterm birth. However, other studies on relationships between interpregnancy interval and preterm birth consistently showed that shorter interpregnancy intervals increase the risk of preterm births. However, the intervals used were not uniform across studies. One study found that, compared to an IPI of 18–23 months, IPIs <3, 3–5, and 6–12 months had higher risks for preterm birth.[34] Another study with median IPI of 36 months showed that, compared to an IPI of 24–36 months, an IPI of <24 months was associated with preterm delivery.[35] Different studies corroborate our finding that multiple births are a risk factor for preterm birth. [18, 36, 37] Similar to our study, others also found male children at higher risk of being preterm[38-40], but a study in Nepal found that female children had a higher risk of being preterm.[18] This study in Nepal enrolled live births in a hospital setting, and had almost half the prevalence of our study. [18] They could have missed more males that had preterm births at home or on the way to a facility.

Different studies in Nepal [18] and outside of Nepal [41-43] have also shown that higher education of mothers decreases the risk of preterm births. Higher education of mothers can lead

For peer review only - http://bmjopen.bmj.com/site/about/guidelines.xhtml

to increased knowledge and awareness regarding pregnancy-related care and thus decrease adverse outcomes of pregnancy. We found that women in the richer wealth quintile had a lower risk of preterm births. Having higher household economic status probably does not directly affect the gestational age at outcome, instead, it probably is mediated by factors like nutrition, physically demanding work during pregnancy, type of care at home, stress level and other psychological factors.[44]

Pregnancy-varying morbidities that significantly decreased the risk of preterm birth in our analysis were respiratory problems in the 3rd trimester; and poor appetite, nausea and vomiting in the 2nd trimester, and the 3rd trimester. The association found with respiratory problems is not clear, as we did not find any studies showing this association in the literature. A study by Wallin et. al. in Nepal showed similar findings - poor appetite, nausea and vomiting in first trimester was not significantly associated with preterm births, but having these symptoms in the 2nd trimester decreased the risk of preterm by 25%.[45]

Pregnancy-varying morbidities that significantly increased the risk of preterm were vaginal bleeding, swelling of hands and face, high diastolic and systolic BP, all in the 3rd trimester. Other studies show similar results for vaginal bleeding. Vaginal bleeding is associated with fetal exposure to oral pathogens, which thereby increases the risk of preterm birth, however, whether bleeding is the cause or result of fetal exposure to oral pathogens is not clear.[46] A prospective cohort study in the US, separating first and second trimesters showed that vaginal bleeding in both trimesters increased the risk of preterm birth by 3.6 times, while bleeding in the second trimester only, was not associated with preterm birth.[47] A systematic review using 23 studies showed that bleeding in early pregnancy increased the risk of preterm births.[48] A study in China showed that vaginal bleeding in the first-trimester increased the risk of preterm births, and the severity, duration and initial timing of vaginal bleeding had different effects on the severity of preterm births.[46] Due to the low enrollment of women in the 1st trimester, we could not look at the association of vaginal bleeding in the 1st trimester with preterm birth. However, all of the above information indicates that vaginal bleeding can be an important predictor of preterm birth and health care workers should recommend appropriate interventions for women if they present with vaginal bleeding (such as more frequent follow up or referral for higher level care).

For peer review only - http://bmjopen.bmj.com/site/about/guidelines.xhtml

Other studies on blood pressure during pregnancy have also shown that a rise in systolic BP (over 30 mm Hg) or diastolic BP (over 15mm Hg), from early pregnancy to the mid third trimester significantly increased the risk of spontaneous preterm birth by 2 to 3 times.[49] Another study showed that an increase in 10 mm Hg in diastolic BP increased the risk of preterm birth by 29%. [50] These indicate the importance of measuring BP during the 3rd trimester. High BP in the 3rd trimester is an indicator of pre-eclampsia/eclampsia and can predict preterm birth. Measuring BP frequently and monitoring the rise and cause of increased BP is important for predicting preterm birth.

For maternal weight, higher weight gain from the 2nd to the 3rd trimester decreased the risk of preterm. This is consistent with a study done outside Nepal, which showed that very low weight gain was strongly associated with very preterm delivery, and that this varied by pre-pregnancy BMI, where underweight women had the highest association and very obese women had lowest association with preterm.[51] Since our study is in a non-obese population, less maternal weight gain can pose a risk to preterm births. Given preterm births have shorter gestation, the increase in weight gain will likely be less because there is less time to increase weight, especially in the third trimester, when much of the gestational weight it gained.

Strengths and Limitations

In this study, GA at outcome has been measured using date of LMP, and not the standard ultrasound (USG) method. However, as LMP was asked at enrollment which was generally early in pregnancy, there is less recall bias than LMP recalled at delivery or late in pregnancy. In addition, the pregnancy surveillance, that asked women if they had their period in the past 5 weeks and administered a pregnancy test if not, may have improved women's recall of date of LMP at the time of enrollment. Women were followed prospectively at monthly intervals to reduce recall bias about pregnancy morbidities and symptoms. In order to reduce misclassification of stillbirths and live births, women were asked whether the infant moved, breathed or cried after birth.

Some variables associated with increased risk of preterm births in previous studies, like a prior pregnancy ending in a preterm birth, gestational diabetes and maternal anemia could not be included in this analysis because they were not measured in the main trial. However, other

For peer review only - http://bmjopen.bmj.com/site/about/guidelines.xhtml

important morbidity variables have been measured and used in the analysis. These risk factors are likely generalizable for similar populations in South Asia.

CONCLUSION

Preterm birth is a leading risk factor for neonatal and under-5 mortality and morbidity worldwide. To reduce neonatal mortality, preventing preterm births can be a vital step. Some of the risk factors from our study are amenable to antenatal interventions but many others need more understanding of the underlying causal mechanisms. Maternal education and awareness can play a role in the long term, while good quality antenatal care, as suggested by the new WHO recommendation of 8 contacts during pregnancy, may help reduce some PTBs. Future research should focus on basic research involving the field of 'omics' using biological samples and implementation research to improve antenatal care and maternal nutrition.

OTHER INFORMATION

Ethics approval and consent to participate

NOMS was approved by the institutional review board (IRB) of the Johns Hopkins Bloomberg School of Public Health in the USA and by the IRB of the Institute of Medicine, Tribhuvan University, Kathmandu, Nepal. This analysis of secondary data was considered exempt by the Johns Hopkins Bloomberg School of Public Health institutional review board (IRB) (FWA00000287). Verbal consent was obtained from women for their participation and their infants for the primary data collection.

Consent for publication:

No individual information including data, images and video are included in this manuscript.

Availability of data and materials:

No data are available.

Competing interests:

The authors declare that they have no competing interests.

For peer review only

Funding source:

NOMS was supported by the National Institutes for Child Health and Development (HD060712) and the Bill & Melinda Gates Foundation (OPP1084399). The analysis for this paper was funded by the National Institutes for Child Health and Development (HD092411). The funders did not have a role in the design of the study, the data collection, nor the analysis, interpretation and writing of the manuscript.

Authors' contribution:
SS, EH, DM, SZ, REB and JK conceptualized and designed the analysis. SS conducted the analysis and wrote the manuscript. LCM, JMT, SKK, SLC and JK were investigators in the parent trial. All authors reviewed results, analysis, discussed interpretations, and contributed to development and revision of the manuscript.

Acknowledgement: We would like to acknowledge study staff and all participants at Nepal Nutritional Intervention Project-Sarlahi site. We would also like to give special thanks to Dr. Leah Horton at Johns Hopkins Bloomberg School of Public Health for her technical contributions during data management.

For peer review only

REFERENCES:

1 Dbstet A. WHO: recommended definitions, terminology and format for statistical tables related to the perinatal period and use of a new certificate for cause of perinatal deaths, *Acta Obstet Gynecol Scand* 1977;56:247-53.

2 Blencowe H, Cousens S, Oestergaard MZ, et al. National, regional, and worldwide estimates of preterm birth rates in the year 2010 with time trends since 1990 for selected countries: a systematic analysis and implications, *The lancet* 2012;379:2162-72.

3 Perin J, Mulick A, Yeung D, et al. Global, regional, and national causes of under-5 mortality in 2000–19: an updated systematic analysis with implications for the Sustainable Development Goals, *The Lancet Child & Adolescent Health* 2022;6:106-15.

4 Chawanpaiboon S, Vogel JP, Moller A, et al. Global, regional, and national estimates of levels of preterm birth in 2014: a systematic review and modelling analysis, *The Lancet Global Health* 2019;7:e37-46.

5 Araújo BFd, Zatti H, Madi JM, et al. Analysis of neonatal morbidity and mortality in late-preterm newborn infants, *J Pediatr* 2012;88:259-66.

6 Mwaniki MK, Atieno M, Lawn JE, et al. Long-term neurodevelopmental outcomes after intrauterine and neonatal insults: a systematic review, *The Lancet* 2012;379:445-52.

7 Vogel JP, Chawanpaiboon S, Moller A, et al. Best Practice & Research Clinical Obstetrics and Gynaecology, *Best Practice & Research Clinical Obstetrics and Gynaecology* 2018;30:1e10.

8 Orchinik LJ, Taylor HG, Espy KA, et al. Cognitive outcomes for extremely preterm/extremely low birth weight children in kindergarten, *Journal of the International Neuropsychological Society* 2011;17:1067-79.

9 Johnson S, Evans TA, Draper ES, et al. Neurodevelopmental outcomes following late and moderate prematurity: a population-based cohort study, *Archives of Disease in Childhood-Fetal and Neonatal Edition* 2015;100:F301-8.

10 Moreira RS, Magalhães LC, Alves CR. Effect of preterm birth on motor development, behavior, and school performance of school-age children: a systematic review☆,☆☆, *J Pediatr* 2014;90:119-34.

11 Petrou S, Abangma G, Johnson S, et al. Costs and health utilities associated with extremely preterm birth: evidence from the EPICure study, *Value in Health* 2009;12:1124-34.

12 Singer LT, Salvator A, Guo S, et al. Maternal psychological distress and parenting stress after the birth of a very low-birth-weight infant, *JAMA* 1999;281:799-805.

For peer review only

13 Family Health Division of the Department of Health Services of the Ministry of Health/Nepal, New ERA/Nepal, Macro O. Nepal Demographic and Health Survey 2001. 2002.

14 Ministry of Health - MOH/Nepal, New ERA/Nepal, ICF. Nepal Demographic and Health Survey 2016. 2017.

15 Ministry of Health and Population - MOHP/Nepal, New ERA/Nepal, International M. Nepal Demographic And Health Survey 2006. 2007.

16 Vogel JP, Chawanpaiboon S, Moller AB, et al. The global epidemiology of preterm birth, *Best Pract Res Clin Obstet Gynaecol* 2018;52:3-12 doi:S1521-6934(18)30079-8 [pii] [published Online First: Oct].

17 National Planning Commission. Sustainable Development Goals 2016–2030: National (Preliminary) Report, *Kathmandu: National Planning Commission* 2015.

18 Gurung A, Wrammert J, Sunny AK, et al. Incidence, risk factors and consequences of preterm birth–findings from a multi-centric observational study for 14 months in Nepal, *Archives of Public Health* 2020;78:1-9.

19 Ashish KC, Wrammert J, Nelin V, et al. Level of mortality risk for babies born preterm or with a small weight for gestation in a tertiary hospital of Nepal, *BMC Public Health* 2015;15:1-9.

20 Anonymous . Nepal: share of rural population. ;2021.

21 Bryce E, Gurung S, Tong H, et al. Population attributable fractions for risk factors for spontaneous preterm births in 81 low-and middle-income countries: A systematic analysis, *Journal of global health* 2022;12.

22 Dahal DR. Social composition of the population: caste/ethnicity and religion in Nepal, *Population monograph of Nepal* 2003;1:87-135.

23 Rutstein SO, Johnson K. The DHS wealth index. DHS comparative reports no. 6, *Calverton: ORC Macro* 2004.

24 Wagura P, Wasunna A, Laving A, et al. Prevalence and factors associated with preterm birth at kenyatta national hospital, *BMC pregnancy and childbirth* 2018;18:1-8.

25 Fuchs F, Monet B, Ducruet T, et al. Effect of maternal age on the risk of preterm birth: A large cohort study, *PloS one* 2018;13:e0191002.

26 Weng Y, Yang C, Chiu Y. Risk assessment of adverse birth outcomes in relation to maternal age, *PloS one* 2014;9:e114843.

27 Muhihi A, Sudfeld CR, Smith ER, et al. Risk factors for small-for-gestational-age and preterm births among 19,269 Tanzanian newborns, *BMC pregnancy and childbirth* 2016;16:1-12.

For peer review only

28 Anderson C, Smitherman AB, Engel SM, et al. Modifiable and non-modifiable risk factors for preterm delivery among adolescent and young adult cancer survivors, *Cancer Causes & Control* 2018;29:289-95.

29 Leonard SA, Crespi CM, Gee DC, et al. Prepregnancy risk factors for preterm birth and the role of maternal nativity in a low-income, Hispanic population, *Matern Child Health J* 2015;19:2295-302.

30 Kildea SV, Gao Y, Rolfe M, et al. Risk factors for preterm, low birthweight and small for gestational age births among Aboriginal women from remote communities in Northern Australia, *Women and Birth* 2017;30:398-405.

31 Delnord M, Blondel B, Prunet C, et al. Are risk factors for preterm and early-term live singleton birth the same? A population-based study in France, *BMJ open* 2018;8:e018745.

32 Salihu H, Mbah AK, Alio AP, et al. Nulliparity and preterm birth in the era of obesity epidemic, *The journal of maternal-fetal & neonatal medicine* 2010;23:1444-50.

33 Ye C, Ruan Y, Zou L, et al. The 2011 survey on hypertensive disorders of pregnancy (HDP) in China: prevalence, risk factors, complications, pregnancy and perinatal outcomes, *PloS one* 2014;9:e100180.

34 Nerlander LM, Callaghan WM, Smith RA, et al. Short interpregnancy interval associated with preterm birth in U S adolescents, *Matern Child Health J* 2015;19:850-8 doi:10.1007/s10995-014-1583-z [doi] [published Online First: Apr].

35 Mahande MJ, Obure J. Effect of interpregnancy interval on adverse pregnancy outcomes in northern Tanzania: a registry-based retrospective cohort study, *BMC pregnancy and childbirth* 2016;16:1-9.

36 Kim J, Cho M, Choi G. Multiple factors in the second trimester of pregnancy on preterm labor symptoms and preterm birth, *Journal of Korean Academy of Nursing* 2017;47:357-66.

37 Shah R, Mullany LC, Darmstadt GL, et al. Incidence and risk factors of preterm birth in a rural Bangladeshi cohort, *BMC pediatrics* 2014;14:1-11.

38 Peelen MJ, Kazemier BM, Ravelli AC, et al. Impact of fetal gender on the risk of preterm birth, a national cohort study, *Acta Obstet Gynecol Scand* 2016;95:1034-41.

39 Zeitlin J, Ancel P, Larroque B, et al. Fetal sex and indicated very preterm birth: results of the EPIPAGE study, *Obstet Gynecol* 2004;190:1322-5 doi:https://doi.org/10.1016/j.ajog.2003.10.703.

40 Lao TT, Sahota DS, Suen SS, et al. The impact of fetal gender on preterm birth in a southern Chinese population, *The Journal of Maternal-Fetal & Neonatal Medicine* 2011;24:1440-3.

41 Poulsen G, Strandberg-Larsen K, Mortensen L, et al. Exploring Educational Disparities in Risk of Preterm Delivery: A Comparative Study of 12 E uropean Birth Cohorts, *Paediatr Perinat Epidemiol* 2015;29:172-83.

For peer review only - http://bmjopen.bmj.com/site/about/guidelines.xhtml

42 Oftedal A, Busterud K, Irgens LM, et al. Socio-economic risk factors for preterm birth in Norway 1999–2009, *Scand J Public Health* 2016;44:587-92.

43 Auger N, Leduc L, Naimi AI, et al. Delivery at term: impact of university education by week of gestation, *Journal of Obstetrics and Gynaecology Canada* 2016;38:118-24.

44 Kramer MS, Goulet L, Lydon J, et al. Socio-economic disparities in preterm birth: causal pathways and mechanisms, *Paediatr Perinat Epidemiol* 2001;15:104-23.

45 Wallin AR, Tielsch JM, Khatry SK, et al. Nausea, vomiting and poor appetite during pregnancy and adverse birth outcomes in rural Nepal: an observational cohort study, *BMC pregnancy and childbirth* 2020;20:1-12.

46 Boggess KA, Moss K, Murtha A, et al. Antepartum vaginal bleeding, fetal exposure to oral pathogens, and risk for preterm birth at *Am J Obstet Gynecol* 2006;194:954-60 doi:S0002-9378(06)00232-8 [pii] [published Online First: Apr].

47 Yang J, Hartmann KE, Savitz DA, et al. Vaginal bleeding during pregnancy and preterm birth, *Am J Epidemiol* 2004;160:118-25 doi:160/2/118 [pii] [published Online First: Jul 15].

48 Hackney DN, Glantz JC. Vaginal bleeding in early pregnancy and preterm birth: systemic review and analysis of heterogeneity, *J Matern Fetal Neonatal Med* 2011;24:778-86 doi:10.3109/14767058.2010.530707 [doi] [published Online First: Jun].

49 Zhang J, Villar J, Sun W, et al. Blood pressure dynamics during pregnancy and spontaneous preterm birth, *Obstet Gynecol* 2007;197:162. e1,162. e6.

50 Chatzi L, Plana E, Daraki V, et al. Metabolic syndrome in early pregnancy and risk of preterm birth, *Am J Epidemiol* 2009;170:829-36.

51 Dietz PM, Callaghan WM, Cogswell ME, et al. Combined effects of prepregnancy body mass index and weight gain during pregnancy on the risk of preterm delivery, *Epidemiology* 2006;17:170-7 doi:00001648-200603000-00011 [pii] [published Online First: Mar].

For peer review only

Figure 1: Flow Diagram for Participants

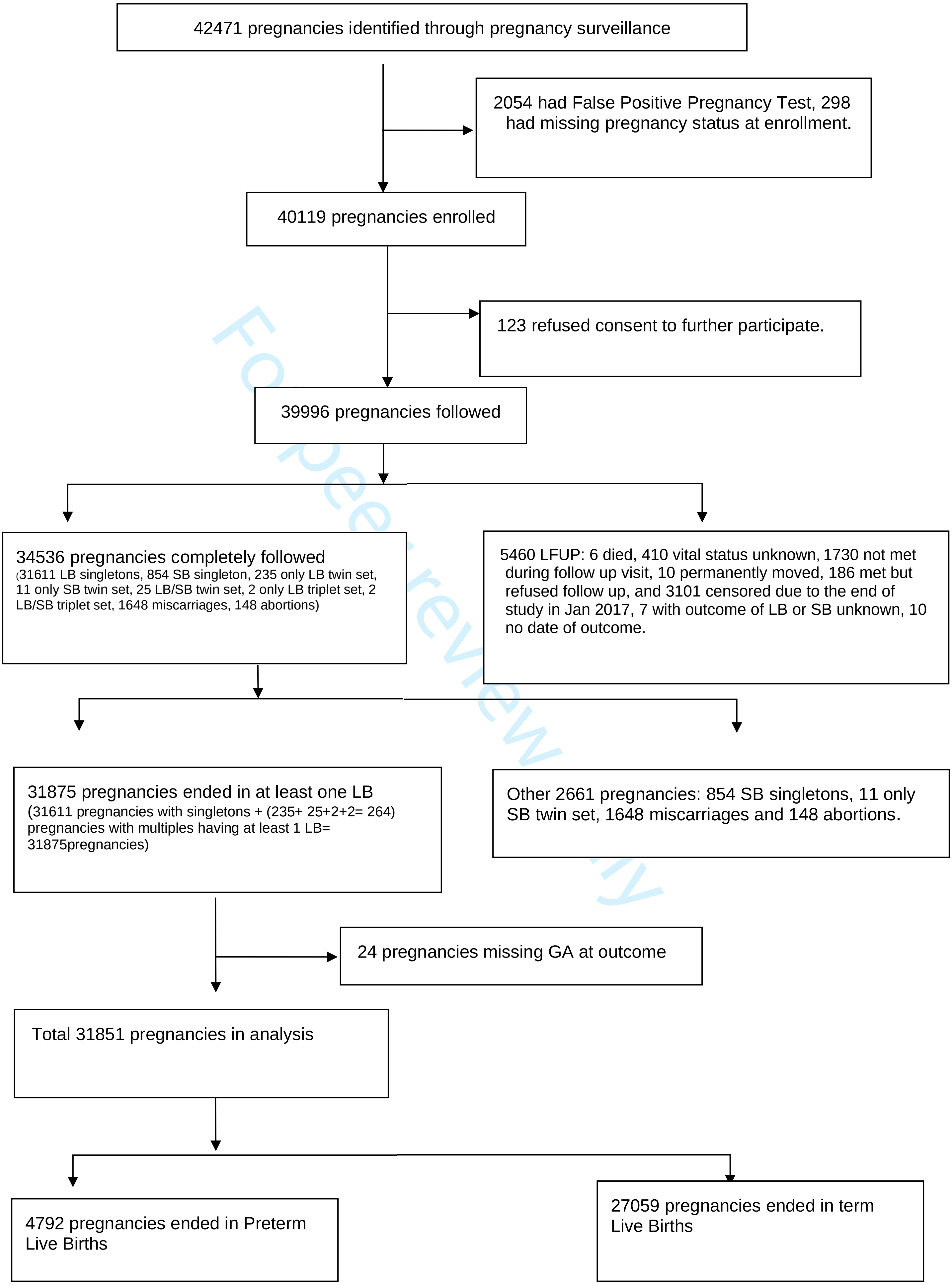

For peer review only

BMJ Open

Prevalence and Predictors of Spontaneous Preterm Births in Nepal- Findings from a prospective, population-based pregnancy cohort in rural Nepal: A secondary data analysis

Journal:	*BMJ Open*
Manuscript ID	bmjopen-2022-066934.R1
Article Type:	Original research
Date Submitted by the Author:	01-Oct-2022
Complete List of Authors:	Subedi, Seema; Johns Hopkins University Bloomberg School of Public Health, Department of International Health; Nepal Nutrition Intervention Project<br>Hazel , Elizabeth; Johns Hopkins University Bloomberg School of Public Health, Department of International Health<br>Mohan, Diwakar; Johns Hopkins University Bloomberg School of Public Health, Department of International Health<br>Zeger, Scott; Johns Hopkins University Bloomberg School of Public Health, Department of Biostatistics<br>Mullany, Luke C.; Johns Hopkins University Bloomberg School of Public Health, Department of International Health<br>Tielsch, James; George Washington University School of Public Health and Health Services, Department of Global Health<br>Khatry, Subarna; Nepal Nutrition Intervention Project<br>LeClerq, Steven C.; Johns Hopkins University Bloomberg School of Public Health, Department of International Health; Nepal Nutrition Intervention Project<br>Black, Robert E.; Johns Hopkins University Bloomberg School of Public Health, Department of International Health<br>Katz, Joanne; Johns Hopkins University Bloomberg School of Public Health, Department of International Health
**Primary Subject Heading**:	Paediatrics
Secondary Subject Heading:	Epidemiology, Public health
Keywords:	OBSTETRICS, Public health < INFECTIOUS DISEASES, Epidemiology < INFECTIOUS DISEASES

SCHOLARONE™
Manuscripts

I, the Submitting Author has the right to grant and does grant on behalf of all authors of the Work (as defined in the below author licence), an exclusive licence and/or a non-exclusive licence for contributions from authors who are: i) UK Crown employees; ii) where BMJ has agreed a CC-BY licence shall apply, and/or iii) in accordance with the terms applicable for US Federal Government officers or employees acting as part of their official duties; on a worldwide, perpetual, irrevocable, royalty-free basis to BMJ Publishing Group Ltd ("BMJ") its licensees and where the relevant Journal is co-owned by BMJ to the co-owners of the Journal, to publish the Work in this journal and any other BMJ products and to exploit all rights, as set out in our licence.

The Submitting Author accepts and understands that any supply made under these terms is made by BMJ to the Submitting Author unless you are acting as an employee on behalf of your employer or a postgraduate student of an affiliated institution which is paying any applicable article publishing charge ("APC") for Open Access articles. Where the Submitting Author wishes to make the Work available on an Open Access basis (and intends to pay the relevant APC), the terms of reuse of such Open Access shall be governed by a Creative Commons licence – details of these licences and which Creative Commons licence will apply to this Work are set out in our licence referred to above.

Other than as permitted in any relevant BMJ Author's Self Archiving Policies, I confirm this Work has not been accepted for publication elsewhere, is not being considered for publication elsewhere and does not duplicate material already published. I confirm all authors consent to publication of this Work and authorise the granting of this licence.

For peer review only

Prevalence and Predictors of Spontaneous Preterm Births in Nepal- Findings from a prospective, population-based pregnancy cohort in rural Nepal: A secondary data analysis

Authors: Seema Subedi[1 4], Elizabeth Hazel[1] Diwakar Mohan[1] Scott Zeger[2], Luke C. Mullany[1], James M. Tielsch[3], Subarna K. Khatry[4], Steven C. LeClerq[1,4], Robert E. Black[1], Joanne Katz[1]

Affiliations:

[1] Department of International Health, Johns Hopkins Bloomberg School of Public Health, 615 N. Wolfe Street, Baltimore, MD, 21205, USA.

[2] Department of Biostatistics, Johns Hopkins Bloomberg School of Public Health, 615 N. Wolfe Street, Baltimore, MD, 21205, USA.

[3] Department of Global Health, Milken Institute School of Public Health, George Washington University, 950 New Hampshire Avenue, Washington, DC, USA

[4] Nepal Nutrition Intervention Project – Sarlahi, Krishna Galli, Lalitpur, Kathmandu, Nepal.

Emails: ssubedi2@jhu.edu, ehazel1@jhu.edu, dmohan3@jhu.edu, sz@jhu.edu, lmullany@gmail.com, jtielsch@email.gwu.edu, skhatry@wlink.com.np, steveleclerq@gmail.com, rblack1@jhu.edu, mailto:jkatz1@jhu.edu

Correspondence: Seema Subedi, MPH. Department of International Health, Johns Hopkins Bloomberg School of Public Health, 615 N. Wolfe Street, Baltimore, MD, 21205, United States.

ssubedi2@jhu.edu

Word Count:

Abstract: 293 words

Main text (excluding tables): 5305

For peer review only

For peer review only

For peer review only - http://bmjopen.bmj.com/site/about/guidelines.xhtml

ABSTRACT

Objective
Preterm birth can have short and long-term complications for a child. Socioeconomic factors and pregnancy-related morbidities may be important to predict and prevent preterm births in low-resource settings. The objective of our study was to find prevalence and predictors of spontaneous preterm birth in rural Nepal.

Design
This is a secondary observational analysis of trial data (registration number NCT01177111)

Setting
Rural Sarlahi district, Nepal

Participants
40,119 pregnant women enrolled from September 9, 2010, to Jan 16, 2017

Outcome Measures
The outcome variable is spontaneous preterm birth. Generalized Estimating Equations (GEE) Poisson regression with robust variance was fitted to present effect estimates as risk ratios.

Result
The prevalence of spontaneous preterm birth was 14.5% (0.5% non-spontaneous). Characteristics not varying in pregnancy associated with increased risk of preterm birth were maternal age less than 18 (ARR=1.13, 95% CI: 1.02-1.26); being Muslim (1.53, 1.16-2.01); first pregnancy (1.15, 1.04-1.28); multiple birth (4.91, 4.20-5.75) and male child (1.10, 1.02-1.17). Those associated with decreased risk were maternal education >5 years (0.81, 0.73-0.90); maternal height >=150 cm (0.89, 0.81-0.98) and being from wealthier families (0.83, 0.74-0.93). Pregnancy related morbidities associated with increased risk of preterm birth were vaginal bleeding (1.53, 1.08-2.18); swelling (1.37, 1.17-1.60); high systolic BP (1.47, 1.08-2.01) and high diastolic BP (1.41, 1.17-1.70) in the 3rd trimester. Those associated with decreased risk were respiratory problem in the 3rd trimester (0.86, 0.79-0.94); and having poor appetite, nausea and vomiting in 2nd trimester (0.86, 0.80-0.92) and 3rd trimester (0.86, 0.79-0.94); and higher weight gain from 2nd to 3rd trimester (0.89, 0.87-0.90).

Conclusion
The prevalence of preterm is high in rural Nepal. Interventions that increase maternal education may play a role. Monitoring morbidities during antenatal care to intervene to reduce them through an effective health system may help reduce preterm.

For peer review only

STRENGTHS AND LIMITATIONS OF THIS STUDY

- This is a large population-based study that allows for analysis of rare and common risk factors for a relatively rare outcome (preterm).
- Previous studies on preterm birth in Nepal were hospital-based, enrolled women during delivery and have explored only the women's socio-demographic factors associated with preterm birth, whereas our study is population-based, enrolls women from earlier in pregnancy, follows them monthly, and has explored symptoms and morbidities variables that change through pregnancy.
- Gestational age (GA) at outcome has been measured using date of last menstrual period (LMP) as usually done in LMICs, however, as LMP was asked at enrollment that was generally early in pregnancy, there is less recall bias than LMP recalled at delivery or late in pregnancy.
- Missing data for second trimester morbidities due to late enrollment of some women in pregnancy is a limitation, but comparison of sociodemographic characteristics suggest limited potential for biases due to this limitation.

For peer review only

For peer review only - http://bmjopen.bmj.com/site/about/guidelines.xhtml

INTRODUCTION:

Preterm birth (PTB) is defined as a birth occurring before 37 completed gestational weeks or fewer than 259 days from a woman's last menstrual period (LMP).[1] In 2010, the global prevalence of preterm birth estimated in 92 countries was 11.1% (95% CI: 9.1%-13.4%), ranging from about 5% in some European countries to 18% in some African countries.[2] Sixty percent of these PTBs occurred in Sub-Saharan Africa and South Asia.[2] Complications of preterm birth was the leading cause of under-5 mortality and accounted for approximately 17.7 % of all under-5 mortality and 36.1% of neonatal mortality, according to the 2019 global estimates. [3] Eighty-one percent of the under-5 deaths from complications of preterm birth occurred in Asia and sub-Saharan Africa countries.[4]

Preterm births can have short and long-term consequences. Short-term consequences comprise increased risks of neonatal respiratory conditions, sepsis, neurological conditions, feeding difficulties, and visual and hearing problems.[5-7] As the child grows, long term consequences include more hospital admissions, poorer neurodevelopment outcomes, difficulties in learning, as well as behavioral and social-emotional problems.[8-10] At the family level, preterm birth can lead to significant economic and psychological difficulties, and at the national level, it leads to significant cost for the health system.[11, 12]

In Nepal, under-five mortality has dropped from 64 deaths to 39 deaths per 1000 live births (LB) from 2001 to 2016.[13-15] In the same period, neonatal mortality rate (NMR) has also steadily declined, (from 39 to 21 per 1,000 LB). [13-15]. Being an important determinant of neonatal mortality, preterm birth has become a greater contributor to under-5 mortality over time.[16] If we do not consider interventions to address preterm births, it would be difficult to achieve Nepal's Sustainable Development Goal (SDG) that aims to reduce the neonatal mortality to 12 per 1000 LB and under-5 mortality to 28 per 1000 LB by 2030.[17]

There are very few studies on the prevalence or risk factors for preterm birth in Nepal,[18, 19] and those that exist have limitations. First, those studies are hospital based. Women enrolled in hospitals during delivery may suffer from systematic recall bias, where women having a preterm birth might report differently from women with term births. Also, at the time of delivery, women might have recall issues in reporting their date of last menstrual period (LMP). Most important, enrolling at facilities has a selection bias, where the preterm births delivered

For peer review only - http://bmjopen.bmj.com/site/about/guidelines.xhtml

at home or on-the-way to facilities are missed, possibly leading to underestimation of the prevalence and a different distribution of risk factors. Second, previous studies have included deliveries taken from urban tertiary hospitals in Nepal. Around 80% of the Nepalese population resides in rural areas[20] and do not have access to delivery services at tertiary centers. Moreover, in rural areas, only 47% of deliveries are assisted by skilled birth attendants. [14] So, the findings from those studies may not be representative of rural Nepal. Third, since the women's enrollment was during delivery, they looked at only risk factors that did not vary in pregnancy and did not analyze changing symptoms, behaviors, and maternal weight gain throughout pregnancy. Some of these symptoms may be indicative of conditions that can be addressed by antenatal care. The objective of our study was to estimate the prevalence and identify predictors/risk factors of spontaneous preterm births in rural Nepal. Understanding and addressing such risk factors is critical to addressing neonatal and child mortality and morbidity, particularly in resource-poor settings like Nepal.

METHODS:

Study Design

This is a secondary data analysis with data taken from the Nepal Oil Massage Study (NOMS), which is a cluster-randomized community-based trial (ClinicalTrials.gov, NCT01177111) on the impact of sunflower seed oil versus standard of care mustard seed oil for neonatal massage on neonatal mortality and morbidity in rural Sarlahi district of Nepal. This study began by identifying married women of childbearing age (15 to 40 years) who consented to pregnancy surveillance. This involved following them every 5 weeks to see whether they became pregnant, based on a positive pregnancy test offered by the study team if a woman reported missing a period. If pregnant, they were consented and enrolled in the trial. During enrollment, demographic data, socioeconomic status, reproductive history, and date of last menstruation were collected. 123 women (0.3%) refused to be followed after enrollment. Those who consented were visited monthly by a field worker until the pregnancy outcome occurred or the study ended. During these monthly visits, field workers asked some basic questions about signs and symptoms of morbidity during the previous 30-day period. At these visits, women also had their weight and blood pressure (BP)/pulse measured, and body temperature recorded. Women reporting signs of morbidity and indicating that these signs were currently present were referred to the local health post or Primary Health Center. Women with fever or elevated blood pressure

For peer review only - http://bmjopen.bmj.com/site/about/guidelines.xhtml

as measured by study staff were similarly referred for care but continued to be included in the study.

As soon as possible after labor began or the baby was delivered, family members or neighbors notified the local female study worker of the birth. She notified a specially trained team who visited the mother and infant as soon after birth as possible. They measured infant weight and time of weight measurement after birth, determined sex of the newborn and whether the baby was a singleton or multiple birth.

Setting and Participants

The study cohort consists of 40,119 pregnancies among married women of child-bearing age, living in 34 Village Development Committees (VDCs) of Sarlahi district, enrolled from September 9, 2010, to Jan 16, 2017, in the NOMS study. Pregnancies were followed monthly until delivery. Live births were categorized as term or preterm. Pregnancies ending in miscarriage, abortion and stillbirths (SB) were excluded from the analysis. Stillbirths were not included because the etiology of these may be quite different from those of preterm births.

Variables

<u>Outcome Variable</u>

The main outcome variable is spontaneous preterm birth among pregnancies that produced at least one live born infant, defined as pregnancies ending less than 259 gestational days from the first day of LMP date. Live births were based on women's self- report. They were asked if the baby moved, cried or breathed after birth. If they said "yes" to one or more of these, the birth was recorded as a live birth. For gestational age (GA), women were asked about their LMP during enrollment, and the GA at outcome was calculated as the difference between reported LMP and the date of the child's birth. Preterm births were classified as spontaneous or non-spontaneous (caesarian section or/and induction), and only spontaneous preterm births were included in the regression analysis.

<u>Independent Variables</u>

Through literature review and expert opinion, certain factors were included in the analysis of predictors. [21] These can be categorized into pregnancy non-varying and pregnancy-varying variables. Pregnancy non-varying variables included sociodemographic, prior pregnancy

For peer review only - http://bmjopen.bmj.com/site/about/guidelines.xhtml

history, current pregnancy and child related variables that do not change during pregnancy. Pregnancy-varying variables included signs and symptoms of morbidity in pregnancy, and maternal weight.

Sociodemographic variables like maternal age at LMP, caste/religion, maternal education, wealth quintile and maternal height were explored. Maternal age was categorized as less than 18, 18-35 and more than 35 years to assess the association of very young women and older women with preterm births. Caste/religion of the mothers (Brahmin /Chhetri, Vaishya, Shudra, Muslim and others) were used as per the caste category system in Nepal. [22] Maternal education (No schooling, 1-5 years and more than 5 years); and maternal height (<145 cm, 145-<150 and ≥150) were used. Household wealth status was measured in quintiles based on a standardized score using principal components analysis of household assets. [23]

Prior pregnancy related variables like parity (1-4, more than 4, prior pregnant but not resulting in live or still birth and no prior pregnant); interpregnancy interval (IPI) defined as the time since the end of last pregnancy to the date of LMP of the current pregnancy, regardless of the outcome (<18, 18-36, and >36 months); any prior live born child who died (No prior LB died and Died); any prior pregnancy that ended in a SB (No prior SB and SB); any prior pregnancy ending in miscarriage (No prior miscarriage and Miscarriage) ; and any prior pregnancy that ended in multiples (No prior multiples and Multiples) were assessed.

Current pregnancy related variables like tobacco intake (ever used any tobacco products during this pregnancy- Yes and No), and alcohol intake (ever used alcohol during this pregnancy- Yes and No) were assessed. Child-level variables like multiple birth (singleton and twin/triplet), and sex of the child (male and female) were included. We used the category with the low risk according to literature of similar settings, to be the reference group if there was no clear hierarchy of risk (such as maternal age, caste) but selected the most at risk group for those where a hierarchy existed (such as maternal education, wealth quintile, maternal height).

Current pregnancy related variables like tobacco and alcohol intake were not included in the regressions because rates of use were very low. Only 0.3% consumed alcohol and only 1.1% used tobacco. Other current pregnancy related variables like number of antenatal care (ANC) visits and place of delivery were shown in descriptive, but omitted from inferential analysis

For peer review only - http://bmjopen.bmj.com/site/about/guidelines.xhtml

because in this setting, women with spontaneous preterm births could have missed the 4th ANC visit in the 9th month and preterm birth could be the cause of a lower number of visits. For place of delivery, spontaneous preterm births were more likely to be delivered at home or on the way to the facility, because many births in this environment are not planned to occur in a facility. However, we also included these variables in the multivariable regressions and provided these as supplemental analyses because ANC may be important in reducing preterm birth.

Symptoms of morbidity during pregnancy such as sexually transmitted diseases (STI), respiratory illness, gastrointestinal (GI) illness, poor appetite, nausea and vomiting, vaginal bleeding, swelling of hands or face, high systolic and diastolic blood pressure were assessed. All these variables were assessed in the 2nd and 3rd trimester, and so labelled as – Problem in at least one visit of the 2nd trimester- Yes or No, and Problem in at least one visit in the 3rd trimester- Yes or No. We did not include symptoms of morbidities in the 1st trimester because only 41% women were enrolled in the 1st trimester, and so 59% missed symptom information in the 1st trimester. Maternal weight gain was defined as the average weight in the 3rd trimester minus the average weight in the 2nd trimester. For measurement of these symptom variables, field workers asked if women had symptoms of morbidity at any time in the past 30 days, at each monthly visit during pregnancy. STI was defined as painful or burning urination, or foul smelling vaginal discharge. Respiratory illness was defined as persistent cough, or difficult or rapid breathing, or wheezing/grunting, or shortness of breath. GI illness was defined as watery stools (4 or more times in a day or blood or white mucus in the stool). Appetite related illness was defined as poor appetite, nausea or vomiting. Vaginal bleeding was defined as spots of blood from the vagina. Swelling was defined as swelling of hands and/or face. Foot/leg swelling was excluded since it is common during pregnancy and not indicative of underlying disease. BP measurements were categorized as high systolic BP if the systolic measurement was >=140 mmHg, and high diastolic BP if diastolic measurement was >=90 mmHg at any monthly visit within the 2nd or 3rd trimester.

Statistical Methods

First, a descriptive analysis was done to show the frequencies of pregnancy non-varying variables (socio demographic, prior pregnancy related, current pregnancy and child related) and pregnancy varying variables (symptoms and maternal weight) by spontaneous preterm and term births. Second, bivariable GEE Poisson regression with robust variance was used to

For peer review only - http://bmjopen.bmj.com/site/about/guidelines.xhtml

examine associations between each risk factor and the outcome to get an unadjusted risk ratio. Since the prevalence of our outcome was more than 10%, we used Poisson regression with robust variance because we wanted to report associations as risk ratios. Third, multivariable GEE Poisson regression with robust variance was used including variables that were significant in the bivariable models, to get the adjusted risk ratios (ARR). GEE was used because in the study, 52% women had multiple pregnancies. Since our unit of analysis is pregnancy and pregnancies were nested within women, women's id variable was used as cluster for GEE modelling.

We included a larger number of potential risk factors to provide a general description of the study population but did not include all of these in the regression analysis. Some variables were highly correlated with each other (such as some reproductive history variables) and we chose just to include one rather than all, and for others, the prevalence was so low that we did not think helpful to include in the regression (for example, smoking and alcohol use). Some of the variables in the unadjusted analysis were not included in the regression because they were not statistically significant in the unadjusted analysis. For example, prior pregnancy ending in miscarriage, stillbirth or a prior multiple birth were not included (as these were highly correlated with each other and not statistically significant in crude models). We did include death of a prior livebirth, which was significant in the crude model.

The descriptive analysis had 31,851 pregnancies. In the regression analysis, we excluded the 1093 pregnancies (3.4%) that ended in caesarian section, induction or both, which leaves 30,758 for analysis. Then, 30.7% out of 30,758 (20.2% missing morbidity in 2nd trimester due to enrollment only in 3rd trimester, 9.4% missing morbidity in 3rd trimester and 1.1% missing other variables) were missing in the regression analysis, and so the final multivariable regression analysis excluded those 9,461 pregnancies, and consisted of 21,297 pregnancies.

Patient and Public Involvement

Patients or the public were not involved in the design, or conduct, or reporting, or dissemination plans of this study.

RESULTS:

Participants

For peer review only

For peer review only - http://bmjopen.bmj.com/site/about/guidelines.xhtml

The analytic population is 31,851 pregnancies that ended in at least one live birth and had information on gestational age at outcome. The detailed flow chart is given in Figure 1. Most women were enrolled in the 1st and 2nd trimester (41% each), followed by the 3rd trimester (18%). Overall, the mean gestational age at enrollment was 18 weeks. For 1st, 2nd and 3rd trimesters, the mean GA at enrollment were 9, 19 and 34 weeks respectively. 52% women (33% with two pregnancies, 14% with three pregnancies, 4% with four pregnancies and 1% with more than four pregnancies) contributed more than one pregnancy to the study.

Descriptive Analysis

For pregnancy non-varying variables, as seen in Table 1, 15% of women were younger (less than 18) and 2% women were older (more than 35 years of age). 9% of women were Muslim caste/religion. Two thirds of women did not go to school, whereas only nearly one fourth had an education of more than five years. 15% of women had height <145cm. About a third (29%) of women had their first pregnancy in this study and 64% had one to four prior live or still births. Among those who had a previous pregnancy, 6% had prior still birth, 16% experienced miscarriage and 16% had a live birth that died, and only 1% had prior multiples. Half the women had an interpregnancy interval of less than 18 months, and 28% of women had four or more ANC visits. Half of the babies were born at home and 2% were born on the way to a facility or outdoors. Only 1.1% consumed tobacco and only 0.3% consumed alcohol during pregnancy. Half of the current pregnancies (51%) resulted in male children, and less than 1% resulted in multiple births. Only 3.4% of pregnancies underwent either caesarian section or induction or both.

For pregnancy-varying variables, as seen in Table 2, poor appetite, nausea and vomiting was the most commonly reported symptom in both the second (39%) and third trimesters (20%); and vaginal bleeding was the least reported symptom (1.2% in the second and 0.6% in the third trimester). Very few women had high systolic blood pressure (0.5% and 0.8%) and high diastolic blood pressure (1.5% and 2.9%) in second and third trimesters respectively. The average weight gained by women from second to third trimester was 3.5 kg.

For peer review only - http://bmjopen.bmj.com/site/about/guidelines.xhtml

Table 1: Distribution of Pregnancy non-varying Variables by preterm and term births

Variables	Categories	Total N=31,851	Term N=27,059	Preterm N=4,792
		N (%)	N (%)	N (%)
Maternal Age at LMP	18 to 35	26,206 (82.3)	22,423 (82.9)	3,783 (78.9)
	Less than 18	4,946 (15.5)	4,100 (15.2)	846 (17.7)
	More than 35	699 2.2)	536 2.0)	163 (3.4)
Caste/Religion	Brahmin and Chhetri	963 (3.0)	857 (3.2)	106 (2.2)
	Vaishya	22,946 (72.0)	19,701 (72.8)	3,245 (67.7)
	Shudra	4,922 (15.5)	4,111 (15.2)	811 (16.9)
	Muslim and others	2,989 (9.4)	2,365 (8.7)	624 (13.0)
	Missing	31 (0.1)	25 (0.1)	6 (0.1)
Maternal Education	No schooling	21,427 (67.3)	17,915 (66.2)	3,512 (73.3)
	1 to 5 years	2,713 (8.5)	2,330 (8.6)	383 (8.0)
	More than 5 years	7,681 (24.1)	6,786 (25.1)	895 (18.7)
	Missing	30 (0.1)	28 (0.1)	2 (0.0)
Quintiles of Wealth	Poorest	6,510 (20.4)	5,340 (19.7)	1,170 (24.4)
	Poor	6,380 (20.0)	5,403 (20.0)	977 (20.4)
	Middle	6,320 (19.8)	5,314 (19.6)	1,006 (21.0)
	Richer	6,296 (19.8)	5,470 (20.2)	826 (17.2)
	Richest	6,324 (19.9)	5,516 (20.4)	808 (16.9)
	Missing	21 (0.1)	16 (0.1)	5 (0.1)
Maternal Height (cms)	<145	4,689 (14.7)	3,885 (14.4)	800 (16.7)
	145-<150	9,559 (29.9)	8,025 (29.7)	1,527 (31.9)
	>=150	17,581 (55.1)	15,111 (55.8)	2,454 (51.2)
	Missing	51 (0.2)	38 (0.14)	11 (0.2)
Parity including both LB and SB, at Enrollment	Parity 1 to 4	20,317 (63.8)	17,366 (64.2)	2,951 (61.6)
	More than 4	1,383 (4.3)	1,117 (4.1)	266 (5.6)
	Prior Pregnant but parity 0	787 (2.5)	672 (2.5)	115 (2.4)
	No Prior Pregnant	9,195 (28.9)	7,769 (28.7)	1,426 (29.8)
	Missing	169 (0.5)	135 (0.5)	34 (0.7)
Interpregnancy Interval based on maternal recall	18 to 36 months	7,927 (24.9)	6,787 (25.1)	1,140 (23.8)
	Less than 18 months	11,461 (36.0)	9,701 (35.9)	1,760 (36.7)
	More than 36 months	3,256 (10.2)	2,794 (10.3)	462 (9.6)
	No Prior Pregnancy	9,195 (28.9)	7,769 (28.7)	1,426 (29.8)
	Missing	12 (0.0)	8 (0.0)	4 (0.1)
Any deaths among Prior LB	Prior LB but not died	17,488 (54.9)	14,999 (55.4)	2,489 (51.9)
	Prior LB died	3,618 (11.4)	2,999 (11.1)	619 (12.9)
	Prior Pregnancy but no LB	1,073 (3.4)	909 (3.4)	164 (3.4)
	No prior pregnancy	9,195 (28.9)	7,769 (28.7)	1,426 (29.8)
	Missing	477 (1.5)	383 (1.4)	94 (2.0)
Any prior pregnancy ended in SB	Prior Pregnancy but no SB	21,270 (66.8)	18,127 (67.0)	3,143 (65.6)
	Prior SB	1,371 (4.3)	1,150 (4.2)	221 (4.6)
	No prior pregnancy	9,195 (28.9)	7,769 (28.7)	1,426 (29.8)
	Missing	15 (0.0)	13 (0.0)	2 (0.0)

For peer review only - http://bmjopen.bmj.com/site/about/guidelines.xhtml

Variables	Categories	Total N=31,851	Term N=27,059	Preterm N=4,792
		N (%)	N (%)	N (%)
Any prior pregnancy ended in miscarriage	Prior Pregnancy but no miscarriage	19,025 (59.7)	16,176 (59.8)	2,849 (59.5)
	Prior miscarriage	3,621 (11.4)	3,104 (11.5)	517 (10.8)
	No prior pregnancy	9,195 (28.9)	7,769 (28.7)	1,426 (29.8)
	Missing	10 (0.0)	10 (0.0)	0 (0.0)
Any prior pregnancy ended in multiples	Prior Pregnancy but no multiples	22,343 (70.1)	19,030 (70.3)	3,313 (69.1)
	Prior multiples	292 (0.9)	241 (0.9)	51 (1.1)
	No prior pregnancy	9,195 (28.9)	7,769 (28.7)	1,426 (29.8)
	Missing	21 (0.1)	19 (0.1)	2 (0.0)
Number of ANC visits	No visit	5,520 (17.3)	4,524 (16.7)	996 (20.8)
	1 visit	4,146 (13.0)	3,420 (12.6)	726 (15.2)
	2-3 visit	9,779 (30.7)	8,158 (30.1)	1,621 (33.8)
	4 or more	8,909 (28.0)	8,021 (29.6)	888 (18.5)
	Missing	3,497 (11.0)	2,936 (10.9)	561 (11.7)
Place of Delivery	Home/Maiti	15,776 (49.5)	13,270 (49.0)	2,506 (52.3)
	HP/Clinic/Hospital	12,016 (37.7)	10,406 (38.5)	1,610 (33.6)
	Way to Facility/Outdoors	610 (1.9)	486 (1.8)	124 (2.6)
	Missing	3,449 (10.8)	2,897 (10.7)	552 (11.5)
Bidi or tobacco use in pregnancy	No	31,498 (98.9)	26,789 (99.0)	4,709 (98.3)
	Yes	353 (1.1)	270 (1.0)	83 (1.7)
Alcohol use (jaard or rakshi) in pregnancy?	No	31,756 (99.7)	26,982 (99.7)	4,774 (99.6)
	Yes	95 (0.3)	77 (0.3)	18 (0.4)
Multiple Birth	Singleton	31,587 (99.2)	26,946 (99.6)	4,641 (96.8)
	Twin/Triplet	264 (0.8)	113 (0.4)	151 (3.2)
Sex of the child	Female	15,182 (47.7)	13,063 (48.3)	2,119 (44.2)
	Male	16,306 (51.2)	13,794 (51.0)	2,512 (52.4)
	Twin/Triplet	264 (0.8)	113 (0.4)	151 (3.2)
	Missing	99 (0.3)	89 (0.3)	10 (0.2)
Induction or CS done	Only Induction	193 (0.6)	166 (0.6)	27 (0.6)
	Only CS	868 (2.7)	735 (2.8)	133 (2.8)
	Both Induction and CS	32 (0.1)	28 (0.1)	4 (0.08)
	None	30,758 (96.6)	26130 (96.6)	4628 (96.6)

For peer review only

Table 2: Distribution of pregnancy-varying variables by preterm and term births

Variables		Total N=31,851	Term N=27,059	Preterm N=4,792
		N (%)	N (%)	N (%)
STI in at least one visit of 2nd trimester?	No	20,823 (65.4)	17,497 (64.7)	3,326 (69.4)
	Yes	4,593 (14.4)	3,855 (14.2)	738 (15.4)
	Missing	6,435 (20.2)	5,707 (21.1)	728 (15.2)
STI in at least one visit of 3rd trimester?	No	25,931 (81.4)	22,512 (83.2)	3,419 (71.3)
	Yes	2,963 (9.3)	2,569 (9.5)	394 (8.2)
	Missing	2,957 (9.3)	1,978 (7.3)	979 (20.4)
Respiratory Problems in at least one visit of 2nd trimester?	No	17,963 (56.4)	15,081 (55.7)	2,882 (60.1)
	Yes	7,452 (23.4)	6,271 (23.2)	1,181 (24.6)
	Missing	6,436 (20.2)	5,707 (21.1)	729 (15.2)
Respiratory Problems in at least one visit of 3rd trimester?	No	22,860 (71.8)	19,743 (73.0)	3,117 (65.0)
	Yes	6,034 (18.9)	5,338 (19.7)	696 (14.5)
	Missing	2,957 9.3)	1,978 (7.3)	979 (20.4)
GI Problems in at least one visit of 2nd trimester?	No	22,742 (71.4)	19,136 (70.7)	3,606 (75.3)
	Yes	2,673 (8.4)	2,216 (8.2)	457 (9.5)
	Missing	6,436 (20.2)	5,707 (21.1)	729 (15.2)
GI Problems in at least one visit of 3rd trimester?	No	26,152 (82.1)	22,712 (83.9)	3,440 (71.8)
	Yes	2,742 (8.6)	2,369 (8.8)	373 (7.8)
	Missing	2,957 (9.3)	1,978 (7.3)	979 (20.4)
Poor appetite, nausea & vomiting in at least one visit of 2nd trimester?	No	13,121 (41.2)	10,814 (40.0)	2,307 (48.1)
	Yes	12,295 (38.6)	10,538 (38.9)	1,757 (36.7)
	Missing	6,435 (20.2)	5,707 (21.1)	728 (15.2)
Poor appetite, nausea & vomiting in at least one visit of 3rd trimester?	No	22,486 (70.6)	19,437 (71.8)	3,049 (63.6)
	Yes	6,409 (20.1)	5,645 (20.9)	764 (15.9)
	Missing	2,956 (9.3)	1,977 (.3)	979 (20.4)
Vaginal Bleeding in at least one visit of 2nd trimester?	No	25,042 (78.6)	21,036 (77.7)	4,006 (83.6)
	Yes	373 (1.2)	315 (1.2)	58 (1.2)
	Missing	6,436 (20.2)	5,708 (21.1)	728 (15.2)
Vaginal Bleeding in at least one visit of 3rd trimester?	No	28,716 (90.2)	24,938 (92.2)	3,778 (78.8)
	Yes	178 (0.6)	143 (0.5)	35 (0.7)
	Missing	2,957 (9.3)	1,978 (7.3)	979 (20.4)
Swelling in at least one visit of 2nd trimester?	No	24,846 (78.0)	20,904 (77.3)	3,942 (82.3)
	Yes	571 (1.8)	448 (1.7)	123 (2.6)
	Missing	6,434 (20.2)	5,707 (21.1)	727 (15.2)

For peer review only

Variables		Total N=31,851	Term N=27,059	Preterm N=4,792
		N (%)	N (%)	N (%)
Swelling in at least one visit of 3rd trimester?	No	27,754 (87.1)	24,126 (89.2)	3,628 (75.7)
	Yes	1,141 (3.6)	956 (3.5)	185 (3.9)
	Missing	2,956 (9.3)	1,977 (7.3)	979 (20.4)
High Systolic BP in 2nd trimester?	Normal Systolic BP	25,260 (79.3)	21,217 (78.4)	4,043 (84.4)
	High Systolic BP	158 (0.5)	136 (0.5)	22 (0.5)
	Missing	6,433 (20.2)	5,706 (21.1)	727 (15.2)
High Systolic BP in 3rd trimester?	Normal Systolic BP	28,659 (90.0)	24,905 (92.0)	3,754 (78.3)
	High Systolic BP	241 (0.8)	181 (0.7)	60 (1.3)
	Missing	2,951 (9.3)	1,973 (7.3)	978 (20.4)
High diastolic BP in 2nd trimester?	Normal diastolic BP	24,945 (78.3)	20,976 (77.5)	3,969 (82.8)
	High diastolic BP	473 (1.5)	377 (1.4)	96 (2.0)
	Missing	6,433 (20.2)	5,706 (21.1)	727 (15.2)
High diastolic BP in 3rd trimester?	Normal diastolic BP	27,982 (87.9)	24,360 (90.0)	3,622 (75.6)
	High diastolic BP	918 (2.9)	726 (2.7)	192 (4.0)
	Missing	2,951 (9.3)	1,973 (7.3)	978 (20.4)
Average weight in 3rd trimester minus Average weight in 2nd trimester in kg (Mean (SD))		3.5 (2.1)	3.6 (2.1)	2.9 (2.2)

For peer review only

For peer review only - http://bmjopen.bmj.com/site/about/guidelines.xhtml

Outcome data

There were 4,792 preterm births out of 31,851 pregnancies with at least one LB. Hence, the prevalence of preterm birth was 15% (95% CI: 14.6%, 15.4%) among the pregnancies enrolled between September 9, 2010, to January 16, 2017. Spontaneous preterm birth was 14.5% and non-spontaneous preterm birth was 0.5%. On looking at severity of spontaneous preterm birth, the prevalence were 0.5%, 1.4% and 2.1% and 10.5% for extreme PTB (<28 weeks), very PTB (28-<32 weeks), moderate PTB (32-<34 weeks) and late PTB (34-<37 weeks) respectively.

Main results

The main results are shown in Table 3. Pregnancy non-varying variables that increased the risk of spontaneous preterm were maternal age less than 18 (ARR=1.13, 95% CI: 1.02-1.26); being Muslim compared to Brahmin and Chhetri (1.53, 1.16-2.01); first pregnancy as compared to parity 1 to 4 (1.15, 1.04-1.28); having a multiple birth (4.91, 4.20-5.75) and having a male child (1.10, 1.02-1.17). Pregnancy non-varying variables that decreased the risk of spontaneous preterm were maternal education of more than 5 years (0.81, 0.73-0.90); maternal height of >=150 cm (0.89, 0.81-0.98) and being wealthier: richer (0.83, 0.74-0.93) wealth quintile compared to the poorest wealth quintile. Pregnancy non-varying variables that showed no association with spontaneous preterm births in the bivariable/unadjusted models are any prior pregnancy ending in SB, any prior pregnancy ending in multiples, and any prior pregnancy ending in miscarriage, and interpregnancy interval. The pregnancy non-varying variable that showed an association in the bivariable model, but not in the multivariable models was any prior pregnancy ending in death for a live birth.

For morbidity symptoms, some increased the risk of preterm, and all of these showed increased risk when symptoms were present in the 3rd trimester. Having vaginal bleeding (ARR= 1.53, 95% CI: 1.08-2.18); swelling (1.37, 1.17-1.60); high systolic BP (1.47, 1.08-2.01) and high diastolic BP (1.41, 1.17-1.70) in the 3rd trimester significantly increased the risk of spontaneous preterm. Some symptom variables significantly decreased the risk of spontaneous preterm. Having respiratory problem in the 3rd trimester (0.86, 0.79-0.94); and having poor appetite, nausea and vomiting in the 2nd trimester (0.86, 0.80-0.92) and in the 3rd trimester (0.86, 0.79-0.94) decreased the risk of spontaneous preterm. Symptom variables that showed no association with spontaneous preterm were STI and GI problems. Symptom variables that were

For peer review only

significant in the bivariable model, but not significant in the multivariable models were swelling in the 2nd trimester and diastolic blood pressure in the 2nd trimester. For maternal weight, higher weight gain from the 2nd to the 3rd trimester was associated with a decreased the risk of spontaneous preterm (0.89, 0.87-0.90).

To examine the possible bias associated with exclusion of pregnancies with missing data, we compared characteristics of women excluded in the regression analysis (n=9,461) (mainly because of missing morbidity in 2nd trimester due to late enrollment) with those included in the regression analysis (n=21,297) (supplementary Table S1).The women excluded in the regression analysis were slightly better off than those included in the regression based on education and socioeconomic status but most relevant, the spontaneous preterm prevalence was 17.9% for those excluded in the regression compared to 13.8% included in the regression.

We also reran the regression model including number of ANC visits. The fewer the number of ANC visits, the higher the risk of spontaneous preterm birth (Table S2). The other regression coefficients did not change in any qualitative way. This could be due to fewer ANC visits putting women at higher risk for spontaneous preterm birth as services provided in ANC (counseling, iron folic acid tablets, blood pressure and weight measurements) are provided less often, but this association may also be due to a shorter duration of pregnancy leading to less time available for ANC visits.

Table 3: Crude and Adjusted Risk Ratios for associations between risk factors and spontaneous preterm birth

**Name of Variables**	**Categories**	**Unadjusted Model**<br>Risk Ratio (95% CI)	**Adjusted Model (N=21,297)**<br>Risk Ratio (95% CI)
Maternal Age at LMP	18 to 35	1	1
	Less than 18	1.19^{***} [1.11,1.28]	1.13^{*} [1.02,1.26]
	More than 35	1.57^{***} [1.36,1.81]	1.22 [0.98,1.51]
Caste/Religion Categories	Brahmin and Chhetri	1	1
	Vaishya	1.33^{**} [1.09,1.62]	1.23 [0.95,1.59]
	Shudra	1.55^{***} [1.26,1.90]	1.23 [0.94,1.62]
	Muslim and others	1.96^{***} [1.60,2.42]	1.53^{**} [1.16,2.01]
Mother's Years of Education	No schooling	1	1
	1 to 5 years	0.86^{**} [0.78,0.95]	0.91 [0.80,1.03]
	More than 5 years	0.71^{***} [0.66,0.76]	0.81^{***} [0.73,0.90]
Quintiles of Wealth	Poorest	1	1
	Poor	0.86^{***} [0.79,0.93]	0.90^{*} [0.82,1.00]
	Middle	0.89^{**} [0.82,0.96]	0.95 [0.86,1.05]

For peer review only

**Name of Variables**	**Categories**	**Unadjusted Model** Risk Ratio (95% CI)	**Adjusted Model (N=21,297)** Risk Ratio (95% CI)
	Richer	0.73*** [0.67,0.79]	0.83** [0.74,0.93]
	Richest	0.71*** [0.65,0.77]	0.88* [0.78,1.00]
Mother's height(centimeter)	<145	1	1
	145-<150	0.93 [0.86,1.01]	0.98 [0.88,1.08]
	>=150	0.81*** [0.75,0.87]	0.89* [0.81,0.98]
Parity including both LB and SB, at Enrollment	Parity 1 to 4	1	1
	More than 4	1.32*** [1.17,1.48]	1.17 [0.99,1.37]
	Prior Pregnant but parity 0	1.02 [0.85,1.22]	0.92 [0.62,1.37]
	No Prior Pregnant	1.10** [1.04,1.17]	1.15** [1.04,1.28]
Interpregnancy Intervals	18 to 36 months	1	1
	Less than 18 months	1.07 [0.99,1.14]	1.08 [0.99,1.18]
	More than 36 months	0.98 [0.89,1.09]	0.9 [0.79,1.02]
	No Prior Pregnancy	1.11** [1.03,1.20]	1 [1.00,1.00]
Any death among prior LB	Prior LB but not died	1	1
	Prior LB died	1.19*** [1.09,1.29]	1.07 [0.97,1.19]
	Prior Pregnancy but no LB	1.07 [0.92,1.25]	1.06 [0.75,1.49]
	No prior pregnancy	1.12*** [1.06,1.19]	1 [1.00,1.00]
Any prior pregnancy ended in SB	Prior Pregnancy but no SB	1	
	Prior SB	1.08 [0.94,1.23]	
	No Prior Pregnancy	1.08** [1.02,1.15]	
Any prior pregnancy ended in miscarriage	Prior Pregnancy but no miscarriage	1	
	Prior miscarriage	0.94 [0.86,1.03]	
	No Prior Pregnancy	1.07* [1.01,1.13]	
Any prior pregnancy ended in multiples	Prior Pregnancy but no multiples	1	
	Prior multiples	1.14 [0.87,1.49]	
	No prior pregnancy	1.08** [1.02,1.14]	
Multiple Birth	Singleton	1	1
	Twin/Triplet	3.92*** [3.52,4.38]	4.91*** [4.20,5.75]
Sex of the child	Female	1	1
	Male	1.10*** [1.04,1.17]	1.10** [1.02,1.17]
	Twin/Triplet	4.13*** [3.69,4.63]	1
STI in at least one visit of 2nd trimester?	No	1	
	Yes	0.99 [0.92,1.07]	
STI in at least one visit of 3rd trimester?	No	1	
	Yes	1.01 [0.92,1.12]	
Respiratory Problems in at least one visit of 2nd trimester?	No	1	1
	Yes	1 [0.94,1.06]	1.08 [1.00,1.16]
Respiratory Problems in at least one visit of 3rd trimester?	No	1	1
	Yes	0.85*** [0.79,0.92]	0.86** [0.79,0.94]
GI Problems in at least one visit of 2nd trimester?	No	1	
	Yes	1.08 [0.98,1.18]	
GI Problems in at least one visit of 3rd trimester?	No	1	
	Yes	1.04 [0.94,1.16]	
Poor appetite, nausea & vomiting in at least one visit of 2nd trimester?	No	1	1
	Yes	0.81*** [0.77,0.86]	0.86*** [0.80,0.92]
Poor appetite, nausea & vomiting in at least	No	1	1

For peer review only

Name of Variables	Categories	Unadjusted Model<br>Risk Ratio (95% CI)	Adjusted Model (N=21,297)<br>Risk Ratio (95% CI)
one visit of 3rd trimester?	Yes	0.88** [0.82,0.95]	0.86*** [0.79,0.94]
Vaginal Bleeding in at least one visit of 2nd trimester?	No	1	1
	Yes	0.91 [0.71,1.17]	0.84 [0.71,1.17]
Vaginal Bleeding in at least one visit of 3rd trimester?	No	1	1
	Yes	1.44* [1.05,1.98]	1.53*[1.08,2.18]
Swelling in at least one visit of 2nd trimester?	No	1	1
	Yes	1.32*** [1.12,1.55]	1.19 [0.98,1.46]
Swelling in at least one visit of 3rd trimester?	No	1	1
	Yes	1.25** [1.09,1.44]	1.37*** [1.17,1.60]
High Systolic BP in 2nd trimester?	Normal Systolic BP	1	1
	High Systolic BP	0.89 [0.59,1.34]	0.67 [0.40,1.12]
High Systolic BP in 3rd trimester?	Normal Systolic BP	1	1
	High Systolic BP	1.92*** [1.52,2.41]	1.47* [1.08,2.01]
High diastolic BP in 2nd trimester?	Normal diastolic BP	1	1
	High diastolic BP	1.34** [1.12,1.60]	1.09 [0.85,1.40]
High diastolic BP in 3rd trimester?	Normal diastolic BP	1	1
	High diastolic BP	1.57*** [1.37,1.80]	1.41*** [1.17,1.70]
Average weight in 3rd trimester minus Average weight in 2nd trimester (kg)		0.88*** [0.87,0.90]	0.89*** [0.87,0.90]

* $p < 0.05$, ** $p < 0.01$, *** $p < 0.001$

For peer review only

For peer review only - http://bmjopen.bmj.com/site/about/guidelines.xhtml

DISCUSSION

Our study is one of the only large-scale studies on preterm births using data from an existing pregnancy surveillance in rural Sarlahi, Nepal. The prevalence of preterm birth is 15%, higher than previous estimates from Nepal [18, 19] which were primarily from urban areas and large hospital-based studies. Our study's strength is that it was population-based and included all home and facility deliveries but is confined to a rural and relatively small geographic area (one third of a district). Our study population is not necessarily representative of all of Nepal, but it is representative of Province 2 in the Terai region within which Sarlahi district is located. For example, the NMR in our study was 31 per 1000 live births. This is similar to the NMR in the 2016 Nepal Demographic Health Survey (NDHS) for Province 2 (30 per 1000). Similarly, 67% of women in our study had no schooling, slightly higher than the 61% in the NDHS for Province 2. NDHS did not provide data on ANC 4+ for Province 2 but rural areas of Nepal had 62% coverage of ANC 4+. It should be noted in our study that health care seeking in pregnancy is low considering the low rates of 4 or more ANC visits (28%) and facility deliveries (38%). The low rates of induction and caesarean section point to a very low proportion of the PTBs being due to iatrogenic causes.

For peer review only

In many other settings, both younger and older maternal age have been reported to be risk factors for preterm birth. [24-30] Being from Muslim caste was positively associated with preterm as compared to Brahmin/Chhetri, which constitutes the major caste in Nepal. Caste/religion is a social construction, and studies in different places have shown that women in minor caste/race/color have higher risk of preterm births. [31-33] It significantly matters what position an individual holds within a society , with regards to occurrence of diseases and also their unequal distribution.[34-36]. First pregnancy (primipara) has been shown to be associated with spontaneous preterm birth in other studies. A study in France showed that primipara as compared to parity 2-3 increased the risk of preterm birth by 1.8 times.[37] Another study in the USA showed that being primipara as compared to multipara increased the risk of very preterm and extremely preterm birth, with the highest risk of 1.37 times for extremely preterm birth.[38] Meta-analysis done using 14 cohort studies from LMICs [39] and a study from sub-saharan African countries [40] also show that primiparity is associated with increased odds of preterm birth. Primipara is a risk factor for hypertensive disorders of pregnancy (HDP), which increases the risk of preterm birth.[41] Our study did not show interpregnancy interval to be the risk factor for spontaneous preterm birth. However, other

For peer review only - http://bmjopen.bmj.com/site/about/guidelines.xhtml

studies on relationships between interpregnancy interval and preterm birth consistently showed that shorter interpregnancy intervals increase the risk of preterm births. However, the intervals used were not uniform across studies. One study found that, compared to an IPI of 18–23 months, IPIs <3, 3–5, and 6–12 months had higher risks for preterm birth.[42] Another study with median IPI of 36 months showed that, compared to an IPI of 24–36 months, an IPI of <24 months was associated with preterm delivery.[43] Different studies corroborate our finding that multiple births are a risk factor for preterm birth. [18, 44, 45] Similar to our study, others also found male children at higher risk of being preterm[46-48], but a study in Nepal found that female children had a higher risk of being preterm.[18] This study in Nepal enrolled live births in a hospital setting, and had almost half the prevalence of our study. [18] They could have missed more males that had preterm births at home or on the way to a facility.

Different studies in Nepal [18] and outside of Nepal [49-51] have also shown that higher education of mothers decreases the risk of preterm births. Higher education of mothers can lead to increased knowledge and awareness regarding pregnancy-related care and thus decrease adverse outcomes of pregnancy. We found greater maternal height to be protective for spontaneous preterm birth, similar to the findings from a meta-analysis done using 12 cohort studies from LMICs. [52] .We found that women in the richer wealth quintile had a lower risk of spontaneous preterm births. Having higher household economic status probably does not directly affect the gestational age at outcome, instead, it probably is mediated by factors like nutrition, physically demanding work during pregnancy, type of care at home, stress level and other psychological factors.[53]

Pregnancy-varying morbidities that significantly decreased the risk of preterm birth in our analysis were respiratory problems in the 3rd trimester; and poor appetite, nausea and vomiting in the 2nd trimester, and the 3rd trimester. On segregating the symptoms within respiratory problems , we found that it was the persistent cough in the 3rd trimester that decreased the risk of preterm. A similar relationship was found between persistent cough and Large for Gestational Age (LGA) in another study done using the same data as ours. [54] However, we could not find any such association in the previous literature. The association might be due to some unmeasured confounders. Or it could be that women with persistent cough in the 3rd trimester made more frequent check-up visits. We saw that 40% of women with persistent cough in the 3rd trimester sought treatment for cough, and almost all had sought treatment

more than once. The pathogenesis of nausea and vomiting in pregnancy is not very clear, but it is broadly accepted to be multifactorial, with the involvement of genetic, endocrine, and gastrointestinal factors. [55] Our findings corroborate with previous findings that nausea and vomiting is associated with reduced risk of preterm birth. [56-59] Specifying by trimesters, a study by Wallin et. al. in Nepal showed similar findings - poor appetite, nausea and vomiting in first trimester was not significantly associated with spontaneous preterm births, but having these symptoms in the 2nd trimester decreased the risk of spontaneous preterm by 25%.[60]

Pregnancy-varying morbidities that significantly increased the risk of spontaneous preterm birth were vaginal bleeding, swelling of hands and face, high diastolic and systolic BP, all in the 3rd trimester. Other studies show similar results for vaginal bleeding. Vaginal bleeding is associated with fetal exposure to oral pathogens, which thereby increases the risk of spontaneous preterm birth, however, whether bleeding is the cause or result of fetal exposure to oral pathogens is not clear.[61] A prospective cohort study, separating first and second trimesters showed that vaginal bleeding in both trimesters increased the risk of preterm birth by 3.6 times, while bleeding in the second trimester only, was not associated with preterm birth.[62] A systematic review using 23 studies showed that bleeding in early pregnancy increased the risk of preterm births.[63] A study in China showed that vaginal bleeding in the first-trimester increased the risk of preterm births, and the severity, duration and initial timing of vaginal bleeding had different effects on the severity of preterm births.[61] Due to the low enrollment of women in the 1st trimester, we could not look at the association of vaginal bleeding in the 1st trimester with spontaneous preterm birth. However, all of the above information indicates that vaginal bleeding can be an important predictor of spontaneous preterm birth and health care workers should recommend appropriate interventions for women if they present with vaginal bleeding (such as more frequent follow up or referral for higher level care).

Other studies on blood pressure during pregnancy have also shown that a rise in systolic BP (over 30 mm Hg) or diastolic BP (over 15mm Hg), from early pregnancy to the mid third trimester significantly increased the risk of spontaneous preterm birth by 2 to 3 times.[64] Another study showed that an increase in 10 mm Hg in diastolic BP increased the risk of preterm birth by 29%. [65] These indicate the importance of measuring BP during the 3rd trimester. High BP in the 3rd trimester is an indicator of pre-eclampsia/eclampsia and can

For peer review only - http://bmjopen.bmj.com/site/about/guidelines.xhtml

predict preterm birth. Measuring BP frequently and monitoring the rise and cause of increased BP is important for predicting spontaneous preterm birth.

For maternal weight, higher weight gain from the 2nd to the 3rd trimester decreased the risk of spontaneous preterm birth. This is consistent with a study done outside Nepal, which showed that very low weight gain was strongly associated with very preterm delivery, and that this varied by pre-pregnancy BMI, where underweight women had the highest association and very obese women had lowest association with preterm.[66] Our study was conducted is a non-obese and undernourished population. We do not have pre-pregnancy BMI, so we looked at the mean BMI in the first trimester. Though the first trimester represents less half of the pregnancies in the study, it hints at undernutrition in the population. The mean BMI was 19.1 kg/m^2 , and 37% had BMI less than 18.5 kg/m^2. So, less maternal weight gain in such population can pose a risk to spontaneous preterm births. Given spontaneous preterm births have shorter gestation, the increase in weight gain will likely be less because there is less time to increase weight, especially in the third trimester, when much of the gestational weight is gained.

Strengths and Limitations

This was a large population-based study that was generally representative of the rural Terai region of Nepal. Multiple variables were collected, including socioeconomic, demographic, pregnancy history, and monthly morbidity in pregnancy that could be examined as risk factors for spontaneous preterm birth. Although there was some missing data in regression analyses, a comparison of those with and without missing data did not show large differences in risk factor prevalence. However, those missing data had higher prevalence of preterm birth. It is possible that if women with missing data were included in the regression, we may have seen stronger associations but the potential bias of these differences is unclear. Gestational age (GA) at birth was measured using date of last menstrual period (LMP) as usually done in the LMICs rather than by ultrasound. However, as LMP was asked at enrollment which was generally early in pregnancy, there is less recall bias than LMP recalled at delivery or late in pregnancy. Using the same method as we used to obtain LMP, Gernand et al. found that LMP based estimates of GA in rural Bangladesh were a mean 2.8 days longer than what was obtained on ultrasound. [67] We therefore believe that this is probably not a significant limitation. Women were

For peer review only - http://bmjopen.bmj.com/site/about/guidelines.xhtml

followed prospectively at monthly intervals to reduce recall bias about pregnancy morbidities and symptoms. In order to reduce misclassification of stillbirths and live births, women were asked whether the infant moved, breathed or cried after birth.

Some variables associated with increased risk of spontaneous preterm births in previous studies, for example, a prior pregnancy ending in a preterm birth, gestational diabetes , maternal anemia and pre-pregnancy maternal nutritional status were not measured in the main trial. However, other important morbidity variables were measured and used in the analysis. We believe these risk factors are likely generalizable for similar populations in South Asia.

CONCLUSION

Preterm birth is a leading risk factor for neonatal and under-5 mortality and morbidity worldwide. To reduce neonatal mortality, preventing preterm births can be a vital step. Some of the risk factors from our study are amenable to antenatal interventions but many others need more understanding of the underlying causal mechanisms. Maternal education and awareness can play a role in the long term, while good quality antenatal care, as suggested by the new WHO recommendation of 8 contacts during pregnancy, may help reduce some PTBs. Future research should focus on basic research involving the field of 'omics' using biological samples and implementation research to improve antenatal care and maternal nutrition.

OTHER INFORMATION

Authors' contribution:

SS, EH, DM, SZ, REB and JK conceptualized and designed the analysis. SS conducted the analysis and wrote the manuscript. LCM, JMT, SKK, SLC and JK were investigators in the parent trial. All authors reviewed results, analysis, discussed interpretations, and contributed to development and revision of the manuscript.

Competing interests:

The authors declare that they have no competing interests.

For peer review only

Funding source:

NOMS was supported by the National Institutes for Child Health and Development (HD060712) and the Bill & Melinda Gates Foundation (OPP1084399). The analysis for this paper was funded by the National Institutes for Child Health and Development (HD092411). The funders did not have a role in the design of the study, the data collection, nor the analysis, interpretation and writing of the manuscript.

Availability of data and materials:

No data are available.

Consent for publication:

No individual information including data, images and video are included in this manuscript.

Ethics approval and consent to participate:

NOMS was approved by the institutional review board (IRB) of the Johns Hopkins Bloomberg School of Public Health in the USA and by the IRB of the Institute of Medicine, Tribhuvan University, Kathmandu, Nepal. This analysis of secondary data was considered exempt by the Johns Hopkins Bloomberg School of Public Health institutional review board (IRB) (FWA00000287). Verbal consent was obtained from women for their participation and their infants for the primary data collection.

Acknowledgement:

We would like to acknowledge study staff and all participants at Nepal Nutritional Intervention Project-Sarlahi site. We would also like to give special thanks to Dr. Leah Horton at Johns Hopkins Bloomberg School of Public Health for her technical contributions during data management.

For peer review only

REFERENCES:

1 Dbstet A. WHO: recommended definitions, terminology and format for statistical tables related to the perinatal period and use of a new certificate for cause of perinatal deaths, *Acta Obstet Gynecol Scand* 1977;56:247-53.

2 Blencowe H, Cousens S, Oestergaard MZ, et al. National, regional, and worldwide estimates of preterm birth rates in the year 2010 with time trends since 1990 for selected countries: a systematic analysis and implications, *The lancet* 2012;379:2162-72.

3 Perin J, Mulick A, Yeung D, et al. Global, regional, and national causes of under-5 mortality in 2000–19: an updated systematic analysis with implications for the Sustainable Development Goals, *The Lancet Child & Adolescent Health* 2022;6:106-15.

4 Chawanpaiboon S, Vogel JP, Moller A, et al. Global, regional, and national estimates of levels of preterm birth in 2014: a systematic review and modelling analysis, *The Lancet Global Health* 2019;7:e37-46.

5 Araújo BFd, Zatti H, Madi JM, et al. Analysis of neonatal morbidity and mortality in late-preterm newborn infants, *J Pediatr* 2012;88:259-66.

6 Mwaniki MK, Atieno M, Lawn JE, et al. Long-term neurodevelopmental outcomes after intrauterine and neonatal insults: a systematic review, *The Lancet* 2012;379:445-52.

7 Vogel JP, Chawanpaiboon S, Moller A, et al. Best Practice & Research Clinical Obstetrics and Gynaecology, *Best Practice & Research Clinical Obstetrics and Gynaecology* 2018;30:1e10.

8 Orchinik LJ, Taylor HG, Espy KA, et al. Cognitive outcomes for extremely preterm/extremely low birth weight children in kindergarten, *Journal of the International Neuropsychological Society* 2011;17:1067-79.

9 Johnson S, Evans TA, Draper ES, et al. Neurodevelopmental outcomes following late and moderate prematurity: a population-based cohort study, *Archives of Disease in Childhood-Fetal and Neonatal Edition* 2015;100:F301-8.

10 Moreira RS, Magalhães LC, Alves CR. Effect of preterm birth on motor development, behavior, and school performance of school-age children: a systematic review☆,☆☆, *J Pediatr* 2014;90:119-34.

11 Petrou S, Abangma G, Johnson S, et al. Costs and health utilities associated with extremely preterm birth: evidence from the EPICure study, *Value in Health* 2009;12:1124-34.

12 Singer LT, Salvator A, Guo S, et al. Maternal psychological distress and parenting stress after the birth of a very low-birth-weight infant, *JAMA* 1999;281:799-805.

13 Family Health Division of the Department of Health Services of the Ministry of Health/Nepal, New ERA/Nepal, Macro O. Nepal Demographic and Health Survey 2001. 2002.

For peer review only - http://bmjopen.bmj.com/site/about/guidelines.xhtml

14 Ministry of Health - MOH/Nepal, New ERA/Nepal, ICF. Nepal Demographic and Health Survey 2016. 2017.

15 Ministry of Health and Population - MOHP/Nepal, New ERA/Nepal, International M. Nepal Demographic And Health Survey 2006. 2007.

16 Vogel JP, Chawanpaiboon S, Moller AB, et al. The global epidemiology of preterm birth, *Best Pract Res Clin Obstet Gynaecol* 2018;52:3-12 doi:S1521-6934(18)30079-8 [pii] [published Online First: Oct].

17 National Planning Commission. Sustainable Development Goals 2016–2030: National (Preliminary) Report, *Kathmandu: National Planning Commission* 2015.

18 Gurung A, Wrammert J, Sunny AK, et al. Incidence, risk factors and consequences of preterm birth–findings from a multi-centric observational study for 14 months in Nepal, *Archives of Public Health* 2020;78:1-9.

19 Ashish KC, Wrammert J, Nelin V, et al. Level of mortality risk for babies born preterm or with a small weight for gestation in a tertiary hospital of Nepal, *BMC Public Health* 2015;15:1-9.

20 Anonymous . Nepal: share of rural population. ;2021.

21 Bryce E, Gurung S, Tong H, et al. Population attributable fractions for risk factors for spontaneous preterm births in 81 low-and middle-income countries: A systematic analysis, *Journal of global health* 2022;12.

22 Dahal DR. Social composition of the population: caste/ethnicity and religion in Nepal, *Population monograph of Nepal* 2003;1:87-135.

23 Rutstein SO, Johnson K. The DHS wealth index. DHS comparative reports no. 6, *Calverton: ORC Macro* 2004.

24 Wagura P, Wasunna A, Laving A, et al. Prevalence and factors associated with preterm birth at kenyatta national hospital, *BMC pregnancy and childbirth* 2018;18:1-8.

25 Fuchs F, Monet B, Ducruet T, et al. Effect of maternal age on the risk of preterm birth: A large cohort study, *PloS one* 2018;13:e0191002.

26 Weng Y, Yang C, Chiu Y. Risk assessment of adverse birth outcomes in relation to maternal age, *PloS one* 2014;9:e114843.

27 Muhihi A, Sudfeld CR, Smith ER, et al. Risk factors for small-for-gestational-age and preterm births among 19,269 Tanzanian newborns, *BMC pregnancy and childbirth* 2016;16:1-12.

28 Anderson C, Smitherman AB, Engel SM, et al. Modifiable and non-modifiable risk factors for preterm delivery among adolescent and young adult cancer survivors, *Cancer Causes & Control* 2018;29:289-95.

For peer review only

29 Leonard SA, Crespi CM, Gee DC, et al. Prepregnancy risk factors for preterm birth and the role of maternal nativity in a low-income, Hispanic population, *Matern Child Health J* 2015;19:2295-302.

30 Kildea SV, Gao Y, Rolfe M, et al. Risk factors for preterm, low birthweight and small for gestational age births among Aboriginal women from remote communities in Northern Australia, *Women and Birth* 2017;30:398-405.

31 Menon R, Dunlop AL, Kramer MR, et al. An overview of racial disparities in preterm birth rates: caused by infection or inflammatory response? *Acta Obstet Gynecol Scand* 2011;90:1325-31.

32 Kramer MR, Hogue CJ, Dunlop AL, et al. Preconceptional stress and racial disparities in preterm birth: an overview, *Acta Obstet Gynecol Scand* 2011;90:1307-16.

33 Patel RR, Steer P, Doyle P, et al. Does gestation vary by ethnic group? A London-based study of over 122 000 pregnancies with spontaneous onset of labour, *Int J Epidemiol* 2004;33:107-13.

34 Goodman AH. Why genes don't count (for racial differences in health). *Am J Public Health* 2000;90:1699.

35 Krieger N. A glossary for social epidemiology, *Journal of Epidemiology & Community Health* 2001;55:693-700.

36 Wilkinson RG, Marmot M. Social determinants of health: the solid facts: World Health Organization 2003.

37 Delnord M, Blondel B, Prunet C, et al. Are risk factors for preterm and early-term live singleton birth the same? A population-based study in France, *BMJ open* 2018;8:e018745.

38 Salihu H, Mbah AK, Alio AP, et al. Nulliparity and preterm birth in the era of obesity epidemic, *The journal of maternal-fetal & neonatal medicine* 2010;23:1444-50.

39 Kozuki N, Lee AC, Silveira MF, et al. The associations of parity and maternal age with small-for-gestational-age, preterm, and neonatal and infant mortality: a meta-analysis, *BMC Public Health* 2013;13:1-10.

40 Alamneh TS, Teshale AB, Worku MG, et al. Preterm birth and its associated factors among reproductive aged women in sub-Saharan Africa: evidence from the recent demographic and health surveys of sub-Sharan African countries, *BMC pregnancy and childbirth* 2021;21:1-11.

41 Ye C, Ruan Y, Zou L, et al. The 2011 survey on hypertensive disorders of pregnancy (HDP) in China: prevalence, risk factors, complications, pregnancy and perinatal outcomes, *PloS one* 2014;9:e100180.

42 Nerlander LM, Callaghan WM, Smith RA, et al. Short interpregnancy interval associated with preterm birth in U S adolescents, *Matern Child Health J* 2015;19:850-8 doi:10.1007/s10995-014-1583-z [doi] [published Online First: Apr].

For peer review only

43 Mahande MJ, Obure J. Effect of interpregnancy interval on adverse pregnancy outcomes in northern Tanzania: a registry-based retrospective cohort study, *BMC pregnancy and childbirth* 2016;16:1-9.

44 Kim J, Cho M, Choi G. Multiple factors in the second trimester of pregnancy on preterm labor symptoms and preterm birth, *Journal of Korean Academy of Nursing* 2017;47:357-66.

45 Shah R, Mullany LC, Darmstadt GL, et al. Incidence and risk factors of preterm birth in a rural Bangladeshi cohort, *BMC pediatrics* 2014;14:1-11.

46 Peelen MJ, Kazemier BM, Ravelli AC, et al. Impact of fetal gender on the risk of preterm birth, a national cohort study, *Acta Obstet Gynecol Scand* 2016;95:1034-41.

47 Zeitlin J, Ancel P, Larroque B, et al. Fetal sex and indicated very preterm birth: results of the EPIPAGE study, *Obstet Gynecol* 2004;190:1322-5 doi:https://doi.org/10.1016/j.ajog.2003.10.703.

48 Lao TT, Sahota DS, Suen SS, et al. The impact of fetal gender on preterm birth in a southern Chinese population, *The Journal of Maternal-Fetal & Neonatal Medicine* 2011;24:1440-3.

49 Poulsen G, Strandberg-Larsen K, Mortensen L, et al. Exploring Educational Disparities in Risk of Preterm Delivery: A Comparative Study of 12 E uropean Birth Cohorts, *Paediatr Perinat Epidemiol* 2015;29:172-83.

50 Oftedal A, Busterud K, Irgens LM, et al. Socio-economic risk factors for preterm birth in Norway 1999–2009, *Scand J Public Health* 2016;44:587-92.

51 Auger N, Leduc L, Naimi AI, et al. Delivery at term: impact of university education by week of gestation, *Journal of Obstetrics and Gynaecology Canada* 2016;38:118-24.

52 Anonymous . Short maternal stature increases risk of small-for-gestational-age and preterm births in low-and middle-income countries: individual participant data meta-analysis and population attributable fraction, *J Nutr* 2015;145:2542-50.

53 Kramer MS, Goulet L, Lydon J, et al. Socio-economic disparities in preterm birth: causal pathways and mechanisms, *Paediatr Perinat Epidemiol* 2001;15:104-23.

54 Hazel EA, Mohan D, Zeger S, et al. Demographic, socio-economic, obstetric, and behavioral factors associated with small-and large-for-gestational-age from a prospective, population-based pregnancy cohort in rural Nepal: a secondary data analysis, *BMC Pregnancy and Childbirth* 2022;22:1-13.

55 Bustos M, Venkataramanan R, Caritis S. Nausea and vomiting of pregnancy - What's new? *Auton Neurosci* 2017;202:62-72 doi:S1566-0702(16)30048-0 [pii] [published Online First: Jan].

56 Tierson FD, Olsen CL, Hook EB. Nausea and vomiting of pregnancy and association with pregnancy outcome, *Obstet Gynecol* 1986;155:1017-22.

For peer review only - http://bmjopen.bmj.com/site/about/guidelines.xhtml

57 Petitti DB. Nausea and pregnancy outcome, *Birth* 1986;13:223-6.

58 Koren G, Clark S, Hankins GD, et al. Maternal safety of the delayed-release doxylamine and pyridoxine combination for nausea and vomiting of pregnancy; a randomized placebo controlled trial, *BMC pregnancy and childbirth* 2015;15:1-6.

59 Klebanoff MA, Koslowe PA, Kaslow R, et al. Epidemiology of vomiting in early pregnancy. *Obstet Gynecol* 1985;66:612-6.

60 Wallin AR, Tielsch JM, Khatry SK, et al. Nausea, vomiting and poor appetite during pregnancy and adverse birth outcomes in rural Nepal: an observational cohort study, *BMC pregnancy and childbirth* 2020;20:1-12.

61 Boggess KA, Moss K, Murtha A, et al. Antepartum vaginal bleeding, fetal exposure to oral pathogens, and risk for preterm birth at *Am J Obstet Gynecol* 2006;194:954-60 doi:S0002-9378(06)00232-8 [pii] [published Online First: Apr].

62 Yang J, Hartmann KE, Savitz DA, et al. Vaginal bleeding during pregnancy and preterm birth, *Am J Epidemiol* 2004;160:118-25 doi:160/2/118 [pii] [published Online First: Jul 15].

63 Hackney DN, Glantz JC. Vaginal bleeding in early pregnancy and preterm birth: systemic review and analysis of heterogeneity, *J Matern Fetal Neonatal Med* 2011;24:778-86 doi:10.3109/14767058.2010.530707 [doi] [published Online First: Jun].

64 Zhang J, Villar J, Sun W, et al. Blood pressure dynamics during pregnancy and spontaneous preterm birth, *Obstet Gynecol* 2007;197:162. e1,162. e6.

65 Chatzi L, Plana E, Daraki V, et al. Metabolic syndrome in early pregnancy and risk of preterm birth, *Am J Epidemiol* 2009;170:829-36.

66 Dietz PM, Callaghan WM, Cogswell ME, et al. Combined effects of prepregnancy body mass index and weight gain during pregnancy on the risk of preterm delivery, *Epidemiology* 2006;17:170-7 doi:00001648-200603000-00011 [pii] [published Online First: Mar].

67 Gernand AD, Paul RR, Ullah B, et al. A home calendar and recall method of last menstrual period for estimating gestational age in rural Bangladesh: a validation study, *Journal of Health, Population and Nutrition* 2016;35:1-9.

For peer review only

Figure 1- Flow Diagram of Participants

For peer review only

For peer review only - http://bmjopen.bmj.com/site/about/guidelines.xhtml

Figure 1: Flow Diagram for Participants

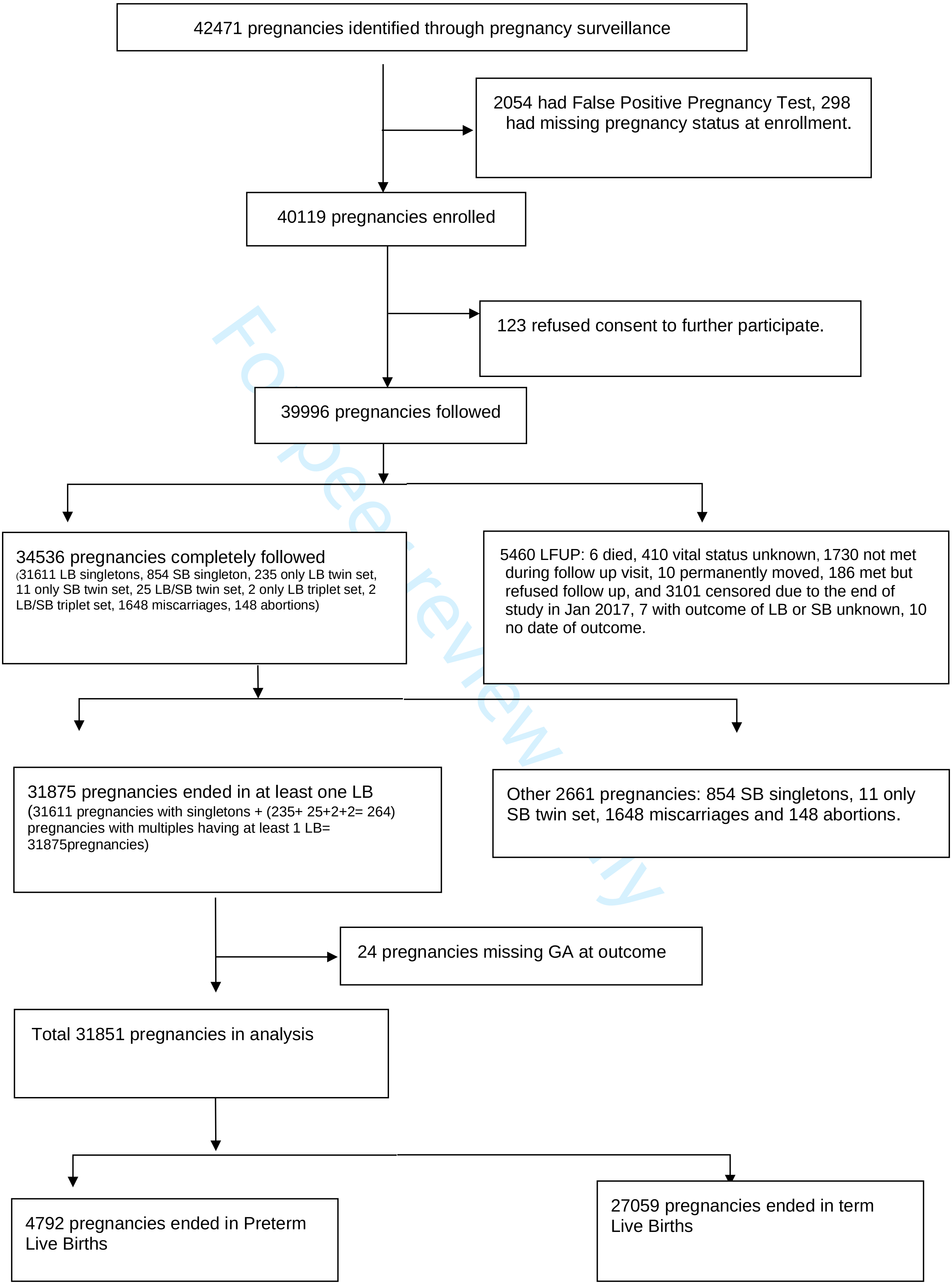

For peer review only

SUPPLEMENTARY TABLES

Table S1. Comparing Pregnancy non-varying Variables by pregnancies Included and Excluded in the Regression Analysis

**Variables**	**Categories**	**Total**<br>**N=30,758**	**Included in regression**<br>**N=21,297**	**Excluded in regression**<br>**N=9,461**	**p-value**
		**N (%)**	**N (%)**	**N (%)**	
Maternal Age at LMP	18 to 35	25,300 (82.3)	17,683 (83.0)	7,617 (80.5)	<0.001
	Less than 18	4,792 (15.6)	3,169 (14.9)	1,623 (17.2)	
	More than 35	666 (2.2)	445 (2.1)	221 (2.3)	
Caste/Ethnicity Categories	Brahmin and Chhetri	879 (2.9)	661 (3.1)	218 (2.3)	<0.001
	Vaishya	22,104 (71.9)	15,412 (72.4)	6,692 (70.7)	
	Shudra	4,826 (15.7)	3,392 (15.9)	1,434 (15.2)	
	Muslim and others	2,919 (9.5)	1,832 (8.6)	1,087 (11.5)	
	Missing	30 (0.1)	0 (0.0)	30 (0.3)	
Mother's Education	No schooling	20,891 (67.9)	14,561 (68.4)	6,330 (66.9)	0.032
	1 to 5 years	2,613 (8.5)	1,819 (8.5)	794 (8.4)	
	More than 5 years	7,224 (23.5)	4,917 (23.1)	2,307 (24.4)	
	Missing	30 (0.1)	0 (0.0)	30 (0.3)	
Quintiles of Wealth	Poorest	6,354 (20.7)	4,414 (20.7)	1,940 (20.5)	0.004
	Poorer	6,210 (20.2)	4,386 (20.6)	1,824 (19.3)	
	Middle	6,152 (20.0)	4,289 (20.1)	1,863 (19.7)	
	Richer	6,036 (19.6)	4,172 (19.6)	1,864 (19.7)	
	Richest	5,985 (19.5)	4,036 (19.0)	1,949 (20.6)	
	Missing	21 (0.1)	0 (0.0)	21 (0.2)	
Maternal Height (centimeter)	<145	4,510 (14.7)	3,193 (15.0)	1,317 (13.9)	0.042
	145-<150	9,227 (30.0)	6,413 (30.1)	2,814 (29.7)	
	>=150	16,974 (55.2)	11,691 (54.9)	5,283 (55.8)	
	Missing	47 (0.2)	0 (0.0)	47 (0.5)	
Parity including both LB and SB, at Enrollment	Parity 1 to 4	19,805 (64.4)	14,137 (66.4)	5,668 (59.9)	<0.001
	More than 4	1,351 (4.4)	875 (4.1)	476 (5.0)	
	Prior Pregnant but parity 0	723 (2.4)	515 (2.4)	208 (2.2)	
	No Prior Pregnant	8,717 (28.3)	5,770 (27.1)	2,947 (31.1)	
	Missing	162 (0.5)	0 (0.0)	162 (1.7)	
Interpregnancy Interval based on maternal recall	18 to 36 months	7,723 (25.1)	5,540 (26.0)	2,183 (23.1)	<0.001
	Less than 18 months	11,201 (36.4)	7,693 (36.1)	3,508 (37.1)	
	More than 36 months	3,106 (10.1)	2,294 (10.8)	812 (8.6)	
	No Prior Pregnancy	8,717 (28.3)	5,770 (27.1)	2,947 (31.1)	
	Missing	11 (0.0)	0 (0.0)	11 (0.1)	
Any deaths among Prior LB	Prior LB but not died	17,089 (55.6)	12,273 (57.6)	4,816 (50.9)	<0.001
	Prior LB died	3,518 (11.4)	2,555 (12.0)	963 (10.2)	
	Prior Pregnancy but no LB	980 (3.2)	699 (3.3)	281 (3.0)	
	No prior pregnancy	8,717 (28.3)	5,770 (27.1)	2,947 (31.1)	
	Missing	454 (1.5)	0 (0.0)	454 (4.8)	
Any prior pregnancy ended in SB	Prior pregnancy but no SB	20,736 (67.4)	14,704 (69.0)	6,032 (63.8)	<0.001

Variables	Categories	Total<br><br>N=30,758	Included in regression<br><br>N=21,297	Excluded in regression<br><br>N=9,461	p-value
	Prior SB	1,291 (4.2)	815 (3.8)	476 (5.0)	
	No prior pregnancy	8,717 (28.3)	5,770 (27.1)	2,947 (31.1)	
	Missing	14 (0.0)	8 (0.0)	6 (0.1)	
Any prior pregnancy ended in miscarriage?	Prior pregnancy but no miscarriage	18,554 (60.3)	12,959 (60.8)	5,595 (59.1)	<0.001
	Prior miscarriage	3,478 (11.3)	2,565 (12.0)	913 (9.7)	
	No prior pregnancy	8,717 (28.3)	5,770 (27.1)	2,947 (31.1)	
	Missing	9 (0.0)	3 (0.0)	6 (0.1)	
Any prior pregnancy ended in multiples?	Prior pregnancy but no multiples	21,735 (70.7)	15,383 (72.2)	6,352 (67.1)	<0.001
	Prior multiples	286 (0.9)	135 (0.6)	151 (1.6)	
	No prior pregnancy	8,717 (28.3)	5,770 (27.1)	2,947 (31.1)	
	Missing	20 (0.1)	9 (0.0)	11 (0.1)	
Number of ANC visits	No visit	5,431 (17.7)	3,788 (17.8)	1,643 (17.4)	<0.001
	1 visit	4,047 (13.2)	2,836 (13.3)	1,211 (12.8)	
	2-3 visit	9,443 (30.7)	6,809 (32.0)	2,634 (27.8)	
	4 or more	8,342 (27.1)	6,532 (30.7)	1,810 (19.1)	
	Missing	3,495 (11.4)	1,332 (6.3)	2,163 (22.9)	
Place of Delivery	Home/Maiti	15,669 (50.9)	11,348 (53.3)	4,321 (45.7)	0.002
	HP/Clinic/Hospital	11,038 (35.9)	8,210 (38.6)	2,828 (29.9)	
	Way to Facility/Outdoors	602 (2.0)	439 (2.1)	163 (1.7)	
	Missing	3,449 (11.2)	1,300 (6.1)	2,149 (22.7)	
Bidi or tobacco use in pregnancy	No	30,410 (98.9)	21,060 (98.9)	9,350 (98.8)	0.64
	Yes	348 (1.1)	237 (1.1)	111 (1.2)	
Alcohol use (jaard or rakshi) in pregnancy?	No	30,665 (99.7)	21,230 (99.7)	9,435 (99.7)	0.56
	Yes	93 (0.3)	67 (0.3)	26 (0.3)	
Multiple Birth	Singleton	30,508 (99.2)	21,147 (99.3)	9,361 (98.9)	0.001
	Twin/Triplet	250 (0.8)	150 (0.7)	100 (1.1)	
Sex of the child	Female	14,673 (47.7)	10,178 (47.8)	4,495 (47.5)	0.004
	Male	15,736 (51.2)	10,969 (51.5)	4,767 (50.4)	
	Twin/Triplet	250 (0.8)	150 (0.7)	100 (1.1)	
	Missing	99 (0.3)	0 (0.0)	99 (1.0)	
Preterm Birth	Term	26,130 (85.0)	18,363 (86.2)	7,767 (82.1)	<0.001
	Preterm	4,628 (15.0)	2,934 (13.8)	1,694 (17.9)	
Gestational Age at outcome in weeks (Mean (SD))		39.4 (3.4)	39.5 (2.7)	39.2 (4.6)	<0.001

For peer review only

Table S2-Comparing the Adjusted Risk Ratios for associations between risk factors and spontaneous preterm birth in different models

**Name of Variables**	**Categories**	**Model 1 Unadjusted Model**<br><br>Risk Ratio (95%CI)	**Model 2 Adjusted Model without ANC/Place of Delivery (N=21,297)**<br><br>Risk Ratio (95%CI)	**Model 3 Adjusted- Added ANC**<br><br>**(N=19,965)**<br><br>Risk Ratio (95%CI)	**Model 4 Adjusted- Added ANC and Place of Delivery**<br><br>**(N=19,964)**<br><br>Risk Ratio (95%CI)
Maternal Age at LMP	18 to 35	1.00 [1.00,1.00]	1.00 [1.00,1.00]	1.00 [1.00,1.00]	1.00 [1.00,1.00]
	Less than 18	1.19*** [1.11,1.28]	1.13* [1.02,1.26]	1.11 [1.00,1.24]	1.11* [1.00,1.24]
	More than 35	1.57*** [1.36,1.81]	1.22 [0.98,1.51]	1.20 [0.97,1.49]	1.20 [0.97,1.49]
Caste/Ethnicity Categories	Brahmin and Chhetri	1.00 [1.00,1.00]	1.00 [1.00,1.00]	1.00 [1.00,1.00]	1.00 [1.00,1.00]
	Vaishya	1.33** [1.09,1.62]	1.23 [0.95,1.59]	1.19 [0.92,1.54]	1.20 [0.92,1.54]
	Shudra	1.55*** [1.26,1.90]	1.23 [0.94,1.62]	1.18 [0.90,1.55]	1.18 [0.90,1.55]
	Muslim and others	1.96*** [1.60,2.42]	1.53** [1.16,2.01]	1.53** [1.16,2.01]	1.53** [1.16,2.02]
Mother's Years of Education	No schooling	1.00 [1.00,1.00]	1.00 [1.00,1.00]	1.00 [1.00,1.00]	1.00 [1.00,1.00]
	1 to 5 years	0.86** [0.78,0.95]	0.91 [0.80,1.03]	0.95 [0.83,1.08]	0.95 [0.83,1.08]
	More than 5 years	0.71*** [0.66,0.76]	0.81*** [0.73,0.90]	0.85** [0.77,0.95]	0.85** [0.76,0.94]
Quintiles of Wealth	Poorest	1.00 [1.00,1.00]	1.00 [1.00,1.00]	1.00 [1.00,1.00]	1.00 [1.00,1.00]
	Poorer	0.86*** [0.79,0.93]	0.90* [0.82,1.00]	0.91 [0.83,1.01]	0.91 [0.83,1.01]
	Middle	0.89** [0.82,0.96]	0.95 [0.86,1.05]	0.98 [0.88,1.08]	0.97 [0.88,1.08]
	Richer	0.73*** [0.67,0.79]	0.83** [0.74,0.93]	0.88* [0.78,0.98]	0.87* [0.78,0.98]
	Richest	0.71*** [0.65,0.77]	0.88* [0.78,1.00]	0.91 [0.80,1.03]	0.90 [0.80,1.02]
Mother's height(centimeter)	<145	1.00 [1.00,1.00]	1.00 [1.00,1.00]	1.00 [1.00,1.00]	1.00 [1.00,1.00]
	145-<150	0.93 [0.86,1.01]	0.98 [0.88,1.08]	0.98 [0.89,1.09]	0.99 [0.89,1.09]
	>=150	0.81*** [0.75,0.87]	0.89* [0.81,0.98]	0.90* [0.82,1.00]	0.91 [0.82,1.00]
Parity including both LB and SB, at Enrollment	Parity 1 to 4	1.00 [1.00,1.00]	1.00 [1.00,1.00]	1.00 [1.00,1.00]	1.00 [1.00,1.00]
	More than 4	1.32*** [1.17,1.48]	1.17 [0.99,1.37]	1.11 [0.95,1.31]	1.12 [0.95,1.31]
	Prior Pregnant but parity 0	1.02 [0.85,1.22]	0.92 [0.62,1.37]	1.12 [0.73,1.73]	1.11 [0.72,1.72]
	No Prior Pregnant	1.10** [1.04,1.17]	1.15** [1.04,1.28]	1.20** [1.07,1.34]	1.19** [1.07,1.33]
Interpregnancy Intervals	18 to 36 months	1.00 [1.00,1.00]	1.00 [1.00,1.00]	1.00 [1.00,1.00]	1.00 [1.00,1.00]
	Less than 18 months	1.07 [0.99,1.14]	1.08 [0.99,1.18]	1.09 [0.99,1.19]	1.09 [0.99,1.19]
	More than 36 months	0.98 [0.89,1.09]	0.90 [0.79,1.02]	0.93 [0.82,1.06]	0.93 [0.82,1.06]
	No Prior Pregnancy	1.11** [1.03,1.20]	1.00 [1.00,1.00]	1.00 [1.00,1.00]	1.00 [1.00,1.00]
Any death among prior LB	Prior LB but not died	1.00 [1.00,1.00]	1.00 [1.00,1.00]	1.00 [1.00,1.00]	1.00 [1.00,1.00]
	Prior LB died	1.19*** [1.09,1.29]	1.07 [0.97,1.19]	1.07 [0.96,1.20]	1.07 [0.96,1.20]
	Prior Pregnancy but no LB	1.07 [0.92,1.25]	1.06 [0.75,1.49]	0.97 [0.66,1.41]	0.96 [0.66,1.41]
	No prior pregnancy	1.12*** [1.06,1.19]			

For peer review only

Name of Variables	Categories	Model 1<br>Unadjusted Model<br><br>Risk Ratio (95%CI)	Model 2<br>Adjusted Model without ANC/Place of Delivery<br>(N=21,297)<br>Risk Ratio (95%CI)	Model 3<br>Adjusted- Added ANC<br><br>(N=19,965)<br>Risk Ratio (95%CI)	Model 4<br>Adjusted- Added ANC and Place of Delivery<br>(N=19,964)<br>Risk Ratio (95%CI)
Any prior pregnancy ended in SB	Prior pregnancy but no SB	1.00 [1.00,1.00]			
	Prior SB	1.08 [0.94,1.23]			
	No prior pregnancy	1.08** [1.02,1.15]			
Any prior pregnancy ended in miscarriage	Prior pregnancy but no miscarriage	1.00 [1.00,1.00]			
	Prior miscarriage	0.94 [0.86,1.03]			
	No prior pregnancy	1.07* [1.01,1.13]			
Any prior pregnancy ended in multiples	Prior pregnancy but no multiples	1.00 [1.00,1.00]			
	Prior multiples	1.14 [0.87,1.49]			
	No prior pregnancy	1.08** [1.02,1.14]			
Number of ANC Visits	No visit	1.00 [1.00,1.00]		1.00 [1.00,1.00]	1.00 [1.00,1.00]
	1 visit	0.98 [0.89,1.07]		1.00 [0.90,1.12]	1.00 [0.90,1.12]
	2-3 visit	0.92* [0.85,0.99]		0.94 [0.86,1.03]	0.93 [0.85,1.02]
	4 or more	0.54*** [0.50,0.59]		0.64*** [0.57,0.71]	0.62*** [0.56,0.70]
Place of Delivery	Home/Maiti	1.00 [1.00,1.00]			1.00 [1.00,1.00]
	HP/Clinic/Hospital	0.84*** [0.79,0.89]			1.04 [0.96,1.12]
	Way to Facility/Outdoors	1.28** [1.09,1.51]			1.23 [1.00,1.52]
Multiple Birth	Singleton	1.00 [1.00,1.00]	1.00 [1.00,1.00]	1.00 [1.00,1.00]	1.00 [1.00,1.00]
	Twin/Triplet	3.92*** [3.52,4.38]	4.91*** [4.20,5.75]	4.97*** [4.25,5.82]	4.96*** [4.24,5.81]
Sex of the Child	Female	1.00 [1.00,1.00]	1.00 [1.00,1.00]	1.00 [1.00,1.00]	1.00 [1.00,1.00]
	Male	1.10*** [1.04,1.17]	1.10** [1.02,1.17]	1.08* [1.01,1.16]	1.08* [1.01,1.16]
	Twin/Triplet	4.13*** [3.69,4.63]	1.00 [1.00,1.00]	1.00 [1.00,1.00]	1.00 [1.00,1.00]
STI in at least one visit of 2nd trimester?	No	1.00 [1.00,1.00]			
	Yes	0.99 [0.92,1.07]			
STI in at least one visit of 3rd trimester?	No	1.00 [1.00,1.00]			
	Yes	1.01 [0.92,1.12]			
Respiratory Problem in at least one visit of 2nd trimester?	No	1.00 [1.00,1.00]	1.00 [1.00,1.00]	1.00 [1.00,1.00]	1.00 [1.00,1.00]
	Yes	1.00 [0.94,1.06]	1.08 [1.00,1.16]	1.09* [1.01,1.18]	1.09* [1.01,1.18]
Respiratory Problem in at least one visit of 3rd trimester?	No	1.00 [1.00,1.00]	1.00 [1.00,1.00]	1.00 [1.00,1.00]	1.00 [1.00,1.00]
	Yes	0.85*** [0.79,0.92]	0.86** [0.79,0.94]	0.86** [0.78,0.94]	0.86** [0.78,0.94]
GI Problem in at least one visit of 2nd trimester?	No	1.00 [1.00,1.00]			
	Yes	1.08 [0.98,1.18]			
GI Problem in at least one visit of 3rd trimester?	No	1.00 [1.00,1.00]			
	Yes	1.04 [0.94,1.16]			

For peer review only

Name of Variables	Categories	Model 1 Unadjusted Model<br><br>Risk Ratio (95%CI)	Model 2 Adjusted Model without ANC/Place of Delivery (N=21,297)<br><br>Risk Ratio (95%CI)	Model 3 Adjusted- Added ANC<br><br>(N=19,965)<br><br>Risk Ratio (95%CI)	Model 4 Adjusted- Added ANC and Place of Delivery<br><br>(N=19,964)<br><br>Risk Ratio (95%CI)
Poor appetite, nausea & vomiting in at least one visit of 2nd trimester?	No	1.00 [1.00,1.00]	1.00 [1.00,1.00]	1.00 [1.00,1.00]	1.00 [1.00,1.00]
	Yes	0.81^{***} [0.77,0.86]	0.86^{***} [0.80,0.92]	0.88^{***} [0.82,0.94]	0.88^{***} [0.81,0.94]
Poor appetite, nausea & vomiting in at least one visit of 3rd trimester?	No	1.00 [1.00,1.00]	1.00 [1.00,1.00]	1.00 [1.00,1.00]	1.00 [1.00,1.00]
	Yes	0.88^{**} [0.82,0.95]	0.86^{***} [0.79,0.94]	0.87^{**} [0.79,0.95]	0.87^{**} [0.79,0.95]
Vaginal Bleeding in at least one visit of 2nd trimester?	No	1.00 [1.00,1.00]	1.00 [1.00,1.00]	1.00 [1.00,1.00]	1.00 [1.00,1.00]
	Yes	0.91 [0.71,1.17]	0.84 [0.62,1.16]	0.83 [0.60,1.14]	0.83 [0.60,1.15]
Vaginal Bleeding in at least one visit of 3rd trimester?	No	1.00 [1.00,1.00]	1.00 [1.00,1.00]	1.00 [1.00,1.00]	1.00 [1.00,1.00]
	Yes	1.44^{*} [1.05,1.98]	1.53^{*} [1.08,2.18]	1.49^{*} [1.04,2.13]	1.50^{*} [1.04,2.15]
Swelling in at least one visit of 2nd trimester?	No	1.00 [1.00,1.00]	1.00 [1.00,1.00]	1.00 [1.00,1.00]	1.00 [1.00,1.00]
	Yes	1.32^{***} [1.12,1.55]	1.19 [0.98,1.46]	1.21 [0.98,1.48]	1.21 [0.99,1.48]
Swelling in at least one visit of 3rd trimester?	No	1.00 [1.00,1.00]	1.00 [1.00,1.00]	1.00 [1.00,1.00]	1.00 [1.00,1.00]
	Yes	1.25^{**} [1.09,1.44]	1.37^{***} [1.17,1.60]	1.36^{***} [1.15,1.60]	1.36^{***} [1.15,1.60]
High Systolic BP in one visit of 2nd trimester?	Normal Systolic BP	1.00 [1.00,1.00]	1.00 [1.00,1.00]	1.00 [1.00,1.00]	1.00 [1.00,1.00]
	High Systolic BP	0.89 [0.59,1.34]	0.67 [0.40,1.12]	0.65 [0.39,1.09]	0.65 [0.39,1.09]
High Systolic BP in one visit of 3rd trimester?	Normal Systolic BP	1.00 [1.00,1.00]	1.00 [1.00,1.00]	1.00 [1.00,1.00]	1.00 [1.00,1.00]
	High Systolic BP	1.92^{***} [1.52,2.41]	1.47^{*} [1.08,2.01]	1.49^{*} [1.08,2.07]	1.49^{*} [1.07,2.07]
High Diastolic BP in one visit of 2nd trimester?	Normal diastolic BP	1.00 [1.00,1.00]	1.00 [1.00,1.00]	1.00 [1.00,1.00]	1.00 [1.00,1.00]
	High diastolic BP	1.34^{**} [1.12,1.60]	1.09 [0.85,1.40]	1.06 [0.82,1.37]	1.06 [0.82,1.38]
High Diastolic BP in one visit of 3rd trimester?	Normal diastolic BP	1.00 [1.00,1.00]	1.00 [1.00,1.00]	1.00 [1.00,1.00]	1.00 [1.00,1.00]
	High diastolic BP	1.57^{***} [1.37,1.80]	1.41^{***} [1.17,1.70]	1.35^{**} [1.12,1.64]	1.35^{**} [1.12,1.64]
Average weight in 3rd trimester minus Average weight in 2nd trimester (kg)		0.88^{***} [0.87,0.90]	0.89^{***} [0.87,0.90]	0.89^{***} [0.87,0.90]	0.89^{***} [0.87,0.90]

* $p < 0.05$, ** $p < 0.01$, *** $p < 0.001$

BMJ Open

Prevalence and Predictors of Spontaneous Preterm Births in Nepal- Findings from a prospective, population-based pregnancy cohort in rural Nepal: A secondary data analysis

Journal:	*BMJ Open*
Manuscript ID	bmjopen-2022-066934.R2
Article Type:	Original research
Date Submitted by the Author:	05-Nov-2022

Complete List of Authors:	Subedi, Seema; Johns Hopkins University Bloomberg School of Public Health, Department of International Health; Nepal Nutrition Intervention Project<br>Hazel , Elizabeth; Johns Hopkins University Bloomberg School of Public Health, Department of International Health<br>Mohan, Diwakar; Johns Hopkins University Bloomberg School of Public Health, Department of International Health<br>Zeger, Scott; Johns Hopkins University Bloomberg School of Public Health, Department of Biostatistics<br>Mullany, Luke C.; Johns Hopkins University Bloomberg School of Public Health, Department of International Health<br>Tielsch, James; George Washington University School of Public Health and Health Services, Department of Global Health<br>Khatry, Subarna; Nepal Nutrition Intervention Project<br>LeClerq, Steven C.; Johns Hopkins University Bloomberg School of Public Health, Department of International Health; Nepal Nutrition Intervention Project<br>Black, Robert E.; Johns Hopkins University Bloomberg School of Public Health, Department of International Health<br>Katz, Joanne; Johns Hopkins University Bloomberg School of Public Health, Department of International Health

**Primary Subject Heading**:	Paediatrics
Secondary Subject Heading:	Epidemiology, Public health
Keywords:	OBSTETRICS, Public health < INFECTIOUS DISEASES, Epidemiology < INFECTIOUS DISEASES

SCHOLARONE™
Manuscripts

I, the Submitting Author has the right to grant and does grant on behalf of all authors of the Work (as defined in the below author licence), an exclusive licence and/or a non-exclusive licence for contributions from authors who are: i) UK Crown employees; ii) where BMJ has agreed a CC-BY licence shall apply, and/or iii) in accordance with the terms applicable for US Federal Government officers or employees acting as part of their official duties; on a worldwide, perpetual, irrevocable, royalty-free basis to BMJ Publishing Group Ltd ("BMJ") its licensees and where the relevant Journal is co-owned by BMJ to the co-owners of the Journal, to publish the Work in this journal and any other BMJ products and to exploit all rights, as set out in our licence.

The Submitting Author accepts and understands that any supply made under these terms is made by BMJ to the Submitting Author unless you are acting as an employee on behalf of your employer or a postgraduate student of an affiliated institution which is paying any applicable article publishing charge ("APC") for Open Access articles. Where the Submitting Author wishes to make the Work available on an Open Access basis (and intends to pay the relevant APC), the terms of reuse of such Open Access shall be governed by a Creative Commons licence – details of these licences and which Creative Commons licence will apply to this Work are set out in our licence referred to above.

Other than as permitted in any relevant BMJ Author's Self Archiving Policies, I confirm this Work has not been accepted for publication elsewhere, is not being considered for publication elsewhere and does not duplicate material already published. I confirm all authors consent to publication of this Work and authorise the granting of this licence.

For peer review only

Prevalence and Predictors of Spontaneous Preterm Births in Nepal- Findings from a prospective, population-based pregnancy cohort in rural Nepal: A secondary data analysis

Authors: Seema Subedi[1 4], Elizabeth Hazel[1] Diwakar Mohan[1] Scott Zeger[2], Luke C. Mullany[1], James M. Tielsch[3], Subarna K. Khatry[4], Steven C. LeClerq[1,4], Robert E. Black[1], Joanne Katz[1]

Affiliations:

[1] Department of International Health, Johns Hopkins Bloomberg School of Public Health, 615 N. Wolfe Street, Baltimore, MD, 21205, USA.

[2] Department of Biostatistics, Johns Hopkins Bloomberg School of Public Health, 615 N. Wolfe Street, Baltimore, MD, 21205, USA.

[3] Department of Global Health, Milken Institute School of Public Health, George Washington University, 950 New Hampshire Avenue, Washington, DC, USA

[4] Nepal Nutrition Intervention Project – Sarlahi, Krishna Galli, Lalitpur, Kathmandu, Nepal.

Emails: ssubedi2@jhu.edu, ehazel1@jhu.edu, dmohan3@jhu.edu, sz@jhu.edu, lmullany@gmail.com, jtielsch@email.gwu.edu, skhatry@wlink.com.np, steveleclerq@gmail.com, rblack1@jhu.edu, mailto:jkatz1@jhu.edu

Correspondence: Seema Subedi, MPH. Department of International Health, Johns Hopkins Bloomberg School of Public Health, 615 N. Wolfe Street, Baltimore, MD, 21205, United States.

ssubedi2@jhu.edu

Word Count:

Abstract: 293 words

Main text (excluding tables): 5382

For peer review only

For peer review only

For peer review only - http://bmjopen.bmj.com/site/about/guidelines.xhtml

ABSTRACT

Objective

Preterm birth can have short and long-term complications for a child. Socioeconomic factors and pregnancy-related morbidities may be important to predict and prevent preterm births in low-resource settings. The objective of our study was to find prevalence and predictors of spontaneous preterm birth in rural Nepal.

Design

This is a secondary observational analysis of trial data (registration number NCT01177111)

Setting

Rural Sarlahi district, Nepal

Participants

40,119 pregnant women enrolled from September 9, 2010, to Jan 16, 2017

Outcome Measures

The outcome variable is spontaneous preterm birth. Generalized Estimating Equations (GEE) Poisson regression with robust variance was fitted to present effect estimates as risk ratios.

Result

The prevalence of spontaneous preterm birth was 14.5% (0.5% non-spontaneous). Characteristics not varying in pregnancy associated with increased risk of preterm birth were maternal age less than 18 (ARR=1.13, 95% CI: 1.02-1.26); being Muslim (1.53, 1.16-2.01); first pregnancy (1.15, 1.04-1.28); multiple birth (4.91, 4.20-5.75) and male child (1.10, 1.02-1.17). Those associated with decreased risk were maternal education >5 years (0.81, 0.73-0.90); maternal height >=150 cm (0.89, 0.81-0.98) and being from wealthier families (0.83, 0.74-0.93). Pregnancy related morbidities associated with increased risk of preterm birth were vaginal bleeding (1.53, 1.08-2.18); swelling (1.37, 1.17-1.60); high systolic BP (1.47, 1.08-2.01) and high diastolic BP (1.41, 1.17-1.70) in the 3rd trimester. Those associated with decreased risk were respiratory problem in the 3rd trimester (0.86, 0.79-0.94); and having poor appetite, nausea and vomiting in 2nd trimester (0.86, 0.80-0.92) and 3rd trimester (0.86, 0.79-0.94); and higher weight gain from 2nd to 3rd trimester (0.89, 0.87-0.90).

Conclusion

The prevalence of preterm is high in rural Nepal. Interventions that increase maternal education may play a role. Monitoring morbidities during antenatal care to intervene to reduce them through an effective health system may help reduce preterm.

For peer review only

STRENGTHS AND LIMITATIONS OF THIS STUDY

- This is a large population-based study that allows for analysis of rare and common risk factors for a relatively rare outcome (preterm).
- Previous studies on preterm birth in Nepal were hospital-based, enrolled women during delivery and have explored only the women's socio-demographic factors associated with preterm birth, whereas our study is population-based, enrolls women from earlier in pregnancy, follows them monthly, and has explored symptoms and morbidities variables that change through pregnancy.
- Gestational age (GA) at outcome has been measured using date of last menstrual period (LMP) as usually done in LMICs, however, as LMP was asked at enrollment that was generally early in pregnancy, there is less recall bias than LMP recalled at delivery or late in pregnancy.
- Missing data for second trimester morbidities due to late enrollment of some women in pregnancy is a limitation, but comparison of sociodemographic characteristics suggest limited potential for biases due to this limitation.

For peer review only

For peer review only - http://bmjopen.bmj.com/site/about/guidelines.xhtml

INTRODUCTION:

Preterm birth (PTB) is defined as a birth occurring before 37 completed gestational weeks or fewer than 259 days from a woman's last menstrual period (LMP).[1] In 2010, the global prevalence of preterm birth estimated in 92 countries was 11.1% (95% CI: 9.1%-13.4%), ranging from about 5% in some European countries to 18% in some African countries.[2] Sixty percent of these PTBs occurred in Sub-Saharan Africa and South Asia.[2] Complications of preterm birth was the leading cause of under-5 mortality and accounted for approximately 17.7 % of all under-5 mortality and 36.1% of neonatal mortality, according to the 2019 global estimates. [3] Eighty-one percent of the under-5 deaths from complications of preterm birth occurred in Asia and sub-Saharan Africa countries.[4]

Preterm births can have short and long-term consequences. Short-term consequences comprise increased risks of neonatal respiratory conditions, sepsis, neurological conditions, feeding difficulties, and visual and hearing problems.[5-7] As the child grows, long term consequences include more hospital admissions, poorer neurodevelopment outcomes, difficulties in learning, as well as behavioral and social-emotional problems.[8-10] At the family level, preterm birth can lead to significant economic and psychological difficulties, and at the national level, it leads to significant cost for the health system.[11, 12]

In Nepal, under-five mortality has dropped from 64 deaths to 39 deaths per 1000 live births (LB) from 2001 to 2016.[13-15] In the same period, neonatal mortality rate (NMR) has also steadily declined, (from 39 to 21 per 1,000 LB). [13-15]. Being an important determinant of neonatal mortality, preterm birth has become a greater contributor to under-5 mortality over time.[16] If we do not consider interventions to address preterm births, it would be difficult to achieve Nepal's Sustainable Development Goal (SDG) that aims to reduce the neonatal mortality to 12 per 1000 LB and under-5 mortality to 28 per 1000 LB by 2030.[17]

There are very few studies on the prevalence or risk factors for preterm birth in Nepal,[18, 19] and those that exist have limitations. First, those studies are hospital based. Women enrolled in hospitals during delivery may suffer from systematic recall bias, where women having a preterm birth might report differently from women with term births. Also, at the time of delivery, women might have recall issues in reporting their date of last menstrual period (LMP). Most important, enrolling at facilities has a selection bias, where the preterm births delivered

For peer review only - http://bmjopen.bmj.com/site/about/guidelines.xhtml

at home or on-the-way to facilities are missed, possibly leading to underestimation of the prevalence and a different distribution of risk factors. Second, previous studies have included deliveries taken from urban tertiary hospitals in Nepal. Around 80% of the Nepalese population resides in rural areas[20] and do not have access to delivery services at tertiary centers. Moreover, in rural areas, only 47% of deliveries are assisted by skilled birth attendants. [14] So, the findings from those studies may not be representative of rural Nepal. Third, since the women's enrollment was during delivery, they looked at only risk factors that did not vary in pregnancy and did not analyze changing symptoms, behaviors, and maternal weight gain throughout pregnancy. Some of these symptoms may be indicative of conditions that can be addressed by antenatal care. The objective of our study was to estimate the prevalence and identify predictors/risk factors of spontaneous preterm births in rural Nepal. Understanding and addressing such risk factors is critical to addressing neonatal and child mortality and morbidity, particularly in resource-poor settings like Nepal.

METHODS:

Study Design

This is a secondary data analysis with data taken from the Nepal Oil Massage Study (NOMS), which is a cluster-randomized community-based trial (ClinicalTrials.gov, NCT01177111) on the impact of sunflower seed oil versus standard of care mustard seed oil for neonatal massage on neonatal mortality and morbidity in rural Sarlahi district of Nepal. This study began by identifying married women of childbearing age (15 to 40 years) who consented to pregnancy surveillance. This involved following them every 5 weeks to see whether they became pregnant, based on a positive pregnancy test offered by the study team if a woman reported missing a period. If pregnant, they were consented and enrolled in the trial. During enrollment, demographic data, socioeconomic status, reproductive history, and date of last menstruation were collected. 123 women (0.3%) refused to be followed after enrollment. Those who consented were visited monthly by a field worker until the pregnancy outcome occurred or the study ended. During these monthly visits, field workers asked some basic questions about signs and symptoms of morbidity during the previous 30-day period. At these visits, women also had their weight and blood pressure (BP)/pulse measured, and body temperature recorded. Women reporting signs of morbidity and indicating that these signs were currently present were referred to the local health post or Primary Health Center. Women with fever or elevated blood pressure

For peer review only - http://bmjopen.bmj.com/site/about/guidelines.xhtml

as measured by study staff were similarly referred for care but continued to be included in the study.

As soon as possible after labor began or the baby was delivered, family members or neighbors notified the local female study worker of the birth. She notified a specially trained team who visited the mother and infant as soon after birth as possible. They measured infant weight and time of weight measurement after birth, determined sex of the newborn and whether the baby was a singleton or multiple birth.

Setting and Participants

The study cohort consists of 40,119 pregnancies among married women of child-bearing age, living in 34 Village Development Committees (VDCs) of Sarlahi district, enrolled from September 9, 2010, to Jan 16, 2017, in the NOMS study. Pregnancies were followed monthly until delivery. Live births were categorized as term or preterm. Pregnancies ending in miscarriage, abortion and stillbirths (SB) were excluded from the analysis. Stillbirths were not included because the etiology of these may be quite different from those of preterm births.

Variables

Outcome Variable

The main outcome variable is spontaneous preterm birth among pregnancies that produced at least one live born infant, defined as pregnancies ending less than 259 gestational days from the first day of LMP date. Live births were based on women's self- report. They were asked if the baby moved, cried or breathed after birth. If they said "yes" to one or more of these, the birth was recorded as a live birth. For gestational age (GA), women were asked about their LMP during enrollment, and the GA at outcome was calculated as the difference between reported LMP and the date of the child's birth. Preterm births were classified as spontaneous or non-spontaneous (caesarian section or/and induction), and only spontaneous preterm births were included in the regression analysis.

Independent Variables

Through literature review and expert opinion, certain factors were included in the analysis of predictors. [21] These can be categorized into pregnancy non-varying and pregnancy-varying variables. Pregnancy non-varying variables included sociodemographic, prior pregnancy

For peer review only - http://bmjopen.bmj.com/site/about/guidelines.xhtml

history, current pregnancy and child related variables that do not change during pregnancy. Pregnancy-varying variables included signs and symptoms of morbidity in pregnancy, and maternal weight.

Sociodemographic variables like maternal age at LMP, caste/religion, maternal education, wealth quintile and maternal height were explored. Maternal age was categorized as less than 18, 18-35 and more than 35 years to assess the association of very young women and older women with preterm births. Caste/religion of the mothers (Brahmin /Chhetri, Vaishya, Shudra, Muslim and others) were used as per the caste category system in Nepal. [22] Maternal education (No schooling, 1-5 years and more than 5 years); and maternal height (<145 cm, 145-<150 and ≥150) were used. Household wealth status was measured in quintiles based on a standardized score using principal components analysis of household assets. [23]

Prior pregnancy related variables like parity (1-4, more than 4, prior pregnant but not resulting in live or still birth and no prior pregnant); interpregnancy interval (IPI) defined as the time since the end of last pregnancy to the date of LMP of the current pregnancy, regardless of the outcome (<18, 18-36, and >36 months); any prior live born child who died (No prior LB died and Died); any prior pregnancy that ended in a SB (No prior SB and SB); any prior pregnancy ending in miscarriage (No prior miscarriage and Miscarriage) ; and any prior pregnancy that ended in multiples (No prior multiples and Multiples) were assessed.

Current pregnancy related variables like tobacco intake (ever used any tobacco products during this pregnancy- Yes and No), and alcohol intake (ever used alcohol during this pregnancy- Yes and No) were assessed. Child-level variables like multiple birth (singleton and twin/triplet), and sex of the child (male and female) were included. We used the category with the low risk according to literature of similar settings, to be the reference group if there was no clear hierarchy of risk (such as maternal age, caste) but selected the most at risk group for those where a hierarchy existed (such as maternal education, wealth quintile, maternal height).

Current pregnancy related variables like tobacco and alcohol intake were not included in the regressions because rates of use were very low. Only 0.3% consumed alcohol and only 1.1% used tobacco. Other current pregnancy related variables like number of antenatal care (ANC) visits and place of delivery were shown in descriptive, but omitted from inferential analysis

For peer review only - http://bmjopen.bmj.com/site/about/guidelines.xhtml

because in this setting, women with spontaneous preterm births could have missed the 4th ANC visit in the 9th month and preterm birth could be the cause of a lower number of visits. For place of delivery, spontaneous preterm births were more likely to be delivered at home or on the way to the facility, because many births in this environment are not planned to occur in a facility. However, we also included these variables in the multivariable regressions and provided these as supplemental analyses because ANC may be important in reducing preterm birth.

Symptoms of morbidity during pregnancy such as sexually transmitted diseases (STI), respiratory illness, gastrointestinal (GI) illness, poor appetite, nausea and vomiting, vaginal bleeding, swelling of hands or face, high systolic and diastolic blood pressure were assessed. All these variables were assessed in the 2nd and 3rd trimester, and so labelled as – Problem in at least one visit of the 2nd trimester- Yes or No, and Problem in at least one visit in the 3rd trimester- Yes or No. We did not include symptoms of morbidities in the 1st trimester because only 41% women were enrolled in the 1st trimester, and so 59% missed symptom information in the 1st trimester. Maternal weight gain was defined as the average weight in the 3rd trimester minus the average weight in the 2nd trimester. For measurement of these symptom variables, field workers asked if women had symptoms of morbidity at any time in the past 30 days, at each monthly visit during pregnancy. STI was defined as painful or burning urination, or foul smelling vaginal discharge. Respiratory illness was defined as persistent cough, or difficult or rapid breathing, or wheezing/grunting, or shortness of breath. GI illness was defined as watery stools (4 or more times in a day or blood or white mucus in the stool). Appetite related illness was defined as poor appetite, nausea or vomiting. Vaginal bleeding was defined as spots of blood from the vagina. Swelling was defined as swelling of hands and/or face. Foot/leg swelling was excluded since it is common during pregnancy and not indicative of underlying disease. BP measurements were categorized as high systolic BP if the systolic measurement was >=140 mmHg, and high diastolic BP if diastolic measurement was >=90 mmHg at any monthly visit within the 2nd or 3rd trimester.

Statistical Methods

First, a descriptive analysis was done to show the frequencies of pregnancy non-varying variables (socio demographic, prior pregnancy related, current pregnancy and child related) and pregnancy varying variables (symptoms and maternal weight) by spontaneous preterm and term births. Second, bivariable GEE Poisson regression with robust variance was used to

For peer review only - http://bmjopen.bmj.com/site/about/guidelines.xhtml

examine associations between each risk factor and the outcome to get an unadjusted risk ratio. Since the prevalence of our outcome was more than 10%, we used Poisson regression with robust variance because we wanted to report associations as risk ratios. Third, multivariable GEE Poisson regression with robust variance was used including variables that were significant in the bivariable models, to get the adjusted risk ratios (ARR). GEE was used because in the study, 52% women had multiple pregnancies. Since our unit of analysis is pregnancy and pregnancies were nested within women, women's id variable was used as cluster for GEE modelling.

For peer review only

We included a larger number of potential risk factors to provide a general description of the study population but did not include all of these in the regression analysis. Some variables were highly correlated with each other (such as some reproductive history variables) and we chose just to include one rather than all, and for others, the prevalence was so low that we did not think helpful to include in the regression (for example, smoking and alcohol use). Some of the variables in the unadjusted analysis were not included in the regression because they were not statistically significant in the unadjusted analysis. For example, prior pregnancy ending in miscarriage, stillbirth or a prior multiple birth were not included (as these were highly correlated with each other and not statistically significant in crude models). We did include death of a prior livebirth, which was significant in the crude model.

The descriptive analysis had 31,851 pregnancies. In the regression analysis, we excluded the 1093 pregnancies (3.4%) that ended in caesarian section, induction or both, which leaves 30,758 for analysis. Then, 30.7% out of 30,758 (20.2% missing morbidity in 2nd trimester due to enrollment only in 3rd trimester, 9.4% missing morbidity in 3rd trimester and 1.1% missing other variables) were missing in the regression analysis, and so the final multivariable regression analysis excluded those 9,461 pregnancies, and consisted of 21,297 pregnancies.

Patient and Public Involvement

Patients or the public were not involved in the design, or conduct, or reporting, or dissemination plans of this study.

RESULTS:

Participants

For peer review only - http://bmjopen.bmj.com/site/about/guidelines.xhtml

The analytic population is 31,851 pregnancies that ended in at least one live birth and had information on gestational age at outcome. The detailed flow chart is given in Figure 1. Most women were enrolled in the 1st and 2nd trimester (41% each), followed by the 3rd trimester (18%). Overall, the mean gestational age at enrollment was 18 weeks. For 1st, 2nd and 3rd trimesters, the mean GA at enrollment were 9, 19 and 34 weeks respectively. 52% women (33% with two pregnancies, 14% with three pregnancies, 4% with four pregnancies and 1% with more than four pregnancies) contributed more than one pregnancy to the study.

Descriptive Analysis

For pregnancy non-varying variables, as seen in Table 1, 15% of women were younger (less than 18) and 2% women were older (more than 35 years of age). 9% of women were Muslim caste/religion. Two thirds of women did not go to school, whereas only nearly one fourth had an education of more than five years. 15% of women had height <145cm. About a third (29%) of women had their first pregnancy in this study and 64% had one to four prior live or still births. Among those who had a previous pregnancy, 6% had prior still birth, 16% experienced miscarriage and 16% had a live birth that died, and only 1% had prior multiples. Half the women had an interpregnancy interval of less than 18 months, and 28% of women had four or more ANC visits. Half of the babies were born at home and 2% were born on the way to a facility or outdoors. Only 1.1% consumed tobacco and only 0.3% consumed alcohol during pregnancy. Half of the current pregnancies (51%) resulted in male children, and less than 1% resulted in multiple births. Only 3.4% of pregnancies underwent either caesarian section or induction or both.

For pregnancy-varying variables, as seen in Table 2, poor appetite, nausea and vomiting was the most commonly reported symptom in both the second (39%) and third trimesters (20%); and vaginal bleeding was the least reported symptom (1.2% in the second and 0.6% in the third trimester). Very few women had high systolic blood pressure (0.5% and 0.8%) and high diastolic blood pressure (1.5% and 2.9%) in second and third trimesters respectively. The average weight gained by women from second to third trimester was 3.5 kg.

For peer review only

Table 1: Distribution of Pregnancy non-varying Variables by preterm and term births

Variables	Categories	Total N=31,851	Term N=27,059	Preterm N=4,792
		N (%)	N (%)	N (%)
Maternal Age at LMP	18 to 35	26,206 (82.3)	22,423 (82.9)	3,783 (78.9)
	Less than 18	4,946 (15.5)	4,100 (15.2)	846 (17.7)
	More than 35	699 2.2)	536 2.0)	163 (3.4)
Caste/Religion	Brahmin and Chhetri	963 (3.0)	857 (3.2)	106 (2.2)
	Vaishya	22,946 (72.0)	19,701 (72.8)	3,245 (67.7)
	Shudra	4,922 (15.5)	4,111 (15.2)	811 (16.9)
	Muslim and others	2,989 (9.4)	2,365 (8.7)	624 (13.0)
	Missing	31 (0.1)	25 (0.1)	6 (0.1)
Maternal Education	No schooling	21,427 (67.3)	17,915 (66.2)	3,512 (73.3)
	1 to 5 years	2,713 (8.5)	2,330 (8.6)	383 (8.0)
	More than 5 years	7,681 (24.1)	6,786 (25.1)	895 (18.7)
	Missing	30 (0.1)	28 (0.1)	2 (0.0)
Quintiles of Wealth	Poorest	6,510 (20.4)	5,340 (19.7)	1,170 (24.4)
	Poor	6,380 (20.0)	5,403 (20.0)	977 (20.4)
	Middle	6,320 (19.8)	5,314 (19.6)	1,006 (21.0)
	Richer	6,296 (19.8)	5,470 (20.2)	826 (17.2)
	Richest	6,324 (19.9)	5,516 (20.4)	808 (16.9)
	Missing	21 (0.1)	16 (0.1)	5 (0.1)
Maternal Height (cms)	<145	4,689 (14.7)	3,885 (14.4)	800 (16.7)
	145-<150	9,559 (29.9)	8,025 (29.7)	1,527 (31.9)
	>=150	17,581 (55.1)	15,111 (55.8)	2,454 (51.2)
	Missing	51 (0.2)	38 (0.14)	11 (0.2)
Parity including both LB and SB, at Enrollment	Parity 1 to 4	20,317 (63.8)	17,366 (64.2)	2,951 (61.6)
	More than 4	1,383 (4.3)	1,117 (4.1)	266 (5.6)
	Prior Pregnant but parity 0	787 (2.5)	672 (2.5)	115 (2.4)
	No Prior Pregnant	9,195 (28.9)	7,769 (28.7)	1,426 (29.8)
	Missing	169 (0.5)	135 (0.5)	34 (0.7)
Interpregnancy Interval based on maternal recall	18 to 36 months	7,927 (24.9)	6,787 (25.1)	1,140 (23.8)
	Less than 18 months	11,461 (36.0)	9,701 (35.9)	1,760 (36.7)
	More than 36 months	3,256 (10.2)	2,794 (10.3)	462 (9.6)
	No Prior Pregnancy	9,195 (28.9)	7,769 (28.7)	1,426 (29.8)
	Missing	12 (0.0)	8 (0.0)	4 (0.1)
Any deaths among Prior LB	Prior LB but not died	17,488 (54.9)	14,999 (55.4)	2,489 (51.9)
	Prior LB died	3,618 (11.4)	2,999 (11.1)	619 (12.9)
	Prior Pregnancy but no LB	1,073 (3.4)	909 (3.4)	164 (3.4)
	No prior pregnancy	9,195 (28.9)	7,769 (28.7)	1,426 (29.8)
	Missing	477 (1.5)	383 (1.4)	94 (2.0)
Any prior pregnancy ended in SB	Prior Pregnancy but no SB	21,270 (66.8)	18,127 (67.0)	3,143 (65.6)
	Prior SB	1,371 (4.3)	1,150 (4.2)	221 (4.6)
	No prior pregnancy	9,195 (28.9)	7,769 (28.7)	1,426 (29.8)
	Missing	15 (0.0)	13 (0.0)	2 (0.0)

For peer review only - http://bmjopen.bmj.com/site/about/guidelines.xhtml
For peer review only

Variables	Categories	Total N=31,851	Term N=27,059	Preterm N=4,792
		N (%)	N (%)	N (%)
Any prior pregnancy ended in miscarriage	Prior Pregnancy but no miscarriage	19,025 (59.7)	16,176 (59.8)	2,849 (59.5)
	Prior miscarriage	3,621 (11.4)	3,104 (11.5)	517 (10.8)
	No prior pregnancy	9,195 (28.9)	7,769 (28.7)	1,426 (29.8)
	Missing	10 (0.0)	10 (0.0)	0 (0.0)
Any prior pregnancy ended in multiples	Prior Pregnancy but no multiples	22,343 (70.1)	19,030 (70.3)	3,313 (69.1)
	Prior multiples	292 (0.9)	241 (0.9)	51 (1.1)
	No prior pregnancy	9,195 (28.9)	7,769 (28.7)	1,426 (29.8)
	Missing	21 (0.1)	19 (0.1)	2 (0.0)
Number of ANC visits	No visit	5,520 (17.3)	4,524 (16.7)	996 (20.8)
	1 visit	4,146 (13.0)	3,420 (12.6)	726 (15.2)
	2-3 visit	9,779 (30.7)	8,158 (30.1)	1,621 (33.8)
	4 or more	8,909 (28.0)	8,021 (29.6)	888 (18.5)
	Missing	3,497 (11.0)	2,936 (10.9)	561 (11.7)
Place of Delivery	Home/Maiti	15,776 (49.5)	13,270 (49.0)	2,506 (52.3)
	HP/Clinic/Hospital	12,016 (37.7)	10,406 (38.5)	1,610 (33.6)
	Way to Facility/Outdoors	610 (1.9)	486 (1.8)	124 (2.6)
	Missing	3,449 (10.8)	2,897 (10.7)	552 (11.5)
Bidi or tobacco use in pregnancy	No	31,498 (98.9)	26,789 (99.0)	4,709 (98.3)
	Yes	353 (1.1)	270 (1.0)	83 (1.7)
Alcohol use (jaard or rakshi) in pregnancy?	No	31,756 (99.7)	26,982 (99.7)	4,774 (99.6)
	Yes	95 (0.3)	77 (0.3)	18 (0.4)
Multiple Birth	Singleton	31,587 (99.2)	26,946 (99.6)	4,641 (96.8)
	Twin/Triplet	264 (0.8)	113 (0.4)	151 (3.2)
Sex of the child	Female	15,182 (47.7)	13,063 (48.3)	2,119 (44.2)
	Male	16,306 (51.2)	13,794 (51.0)	2,512 (52.4)
	Twin/Triplet	264 (0.8)	113 (0.4)	151 (3.2)
	Missing	99 (0.3)	89 (0.3)	10 (0.2)
Induction or CS done	Only Induction	193 (0.6)	166 (0.6)	27 (0.6)
	Only CS	868 (2.7)	735 (2.8)	133 (2.8)
	Both Induction and CS	32 (0.1)	28 (0.1)	4 (0.08)
	None	30,758 (96.6)	26130 (96.6)	4628 (96.6)

For peer review only

Table 2: Distribution of pregnancy-varying variables by preterm and term births

Variables		Total N=31,851	Term N=27,059	Preterm N=4,792
		N (%)	N (%)	N (%)
STI in at least one visit of 2nd trimester?	No	20,823 (65.4)	17,497 (64.7)	3,326 (69.4)
	Yes	4,593 (14.4)	3,855 (14.2)	738 (15.4)
	Missing	6,435 (20.2)	5,707 (21.1)	728 (15.2)
STI in at least one visit of 3rd trimester?	No	25,931 (81.4)	22,512 (83.2)	3,419 (71.3)
	Yes	2,963 (9.3)	2,569 (9.5)	394 (8.2)
	Missing	2,957 (9.3)	1,978 (7.3)	979 (20.4)
Respiratory Problems in at least one visit of 2nd trimester?	No	17,963 (56.4)	15,081 (55.7)	2,882 (60.1)
	Yes	7,452 (23.4)	6,271 (23.2)	1,181 (24.6)
	Missing	6,436 (20.2)	5,707 (21.1)	729 (15.2)
Respiratory Problems in at least one visit of 3rd trimester?	No	22,860 (71.8)	19,743 (73.0)	3,117 (65.0)
	Yes	6,034 (18.9)	5,338 (19.7)	696 (14.5)
	Missing	2,957 9.3)	1,978 (7.3)	979 (20.4)
GI Problems in at least one visit of 2nd trimester?	No	22,742 (71.4)	19,136 (70.7)	3,606 (75.3)
	Yes	2,673 (8.4)	2,216 (8.2)	457 (9.5)
	Missing	6,436 (20.2)	5,707 (21.1)	729 (15.2)
GI Problems in at least one visit of 3rd trimester?	No	26,152 (82.1)	22,712 (83.9)	3,440 (71.8)
	Yes	2,742 (8.6)	2,369 (8.8)	373 (7.8)
	Missing	2,957 (9.3)	1,978 (7.3)	979 (20.4)
Poor appetite, nausea & vomiting in at least one visit of 2nd trimester?	No	13,121 (41.2)	10,814 (40.0)	2,307 (48.1)
	Yes	12,295 (38.6)	10,538 (38.9)	1,757 (36.7)
	Missing	6,435 (20.2)	5,707 (21.1)	728 (15.2)
Poor appetite, nausea & vomiting in at least one visit of 3rd trimester?	No	22,486 (70.6)	19,437 (71.8)	3,049 (63.6)
	Yes	6,409 (20.1)	5,645 (20.9)	764 (15.9)
	Missing	2,956 (9.3)	1,977 (.3)	979 (20.4)
Vaginal Bleeding in at least one visit of 2nd trimester?	No	25,042 (78.6)	21,036 (77.7)	4,006 (83.6)
	Yes	373 (1.2)	315 (1.2)	58 (1.2)
	Missing	6,436 (20.2)	5,708 (21.1)	728 (15.2)
Vaginal Bleeding in at least one visit of 3rd trimester?	No	28,716 (90.2)	24,938 (92.2)	3,778 (78.8)
	Yes	178 (0.6)	143 (0.5)	35 (0.7)
	Missing	2,957 (9.3)	1,978 (7.3)	979 (20.4)
Swelling in at least one visit of 2nd trimester?	No	24,846 (78.0)	20,904 (77.3)	3,942 (82.3)
	Yes	571 (1.8)	448 (1.7)	123 (2.6)
	Missing	6,434 (20.2)	5,707 (21.1)	727 (15.2)

For peer review only

Variables		Total N=31,851	Term N=27,059	Preterm N=4,792
		N (%)	N (%)	N (%)
Swelling in at least one visit of 3rd trimester?	No	27,754 (87.1)	24,126 (89.2)	3,628 (75.7)
	Yes	1,141 (3.6)	956 (3.5)	185 (3.9)
	Missing	2,956 (9.3)	1,977 (7.3)	979 (20.4)
High Systolic BP in 2nd trimester?	Normal Systolic BP	25,260 (79.3)	21,217 (78.4)	4,043 (84.4)
	High Systolic BP	158 (0.5)	136 (0.5)	22 (0.5)
	Missing	6,433 (20.2)	5,706 (21.1)	727 (15.2)
High Systolic BP in 3rd trimester?	Normal Systolic BP	28,659 (90.0)	24,905 (92.0)	3,754 (78.3)
	High Systolic BP	241 (0.8)	181 (0.7)	60 (1.3)
	Missing	2,951 (9.3)	1,973 (7.3)	978 (20.4)
High diastolic BP in 2nd trimester?	Normal diastolic BP	24,945 (78.3)	20,976 (77.5)	3,969 (82.8)
	High diastolic BP	473 (1.5)	377 (1.4)	96 (2.0)
	Missing	6,433 (20.2)	5,706 (21.1)	727 (15.2)
High diastolic BP in 3rd trimester?	Normal diastolic BP	27,982 (87.9)	24,360 (90.0)	3,622 (75.6)
	High diastolic BP	918 (2.9)	726 (2.7)	192 (4.0)
	Missing	2,951 (9.3)	1,973 (7.3)	978 (20.4)
Average weight in 3rd trimester minus Average weight in 2nd trimester in kg (Mean (SD))		3.5 (2.1)	3.6 (2.1)	2.9 (2.2)

For peer review only

Outcome data

There were 4,792 preterm births out of 31,851 pregnancies with at least one LB. Hence, the prevalence of preterm birth was 15% (95% CI: 14.6%, 15.4%) among the pregnancies enrolled between September 9, 2010, to January 16, 2017. Spontaneous preterm birth was 14.5% and non-spontaneous preterm birth was 0.5%. On looking at severity of spontaneous preterm birth, the prevalence were 0.5%, 1.4% and 2.1% and 10.5% for extreme PTB (<28 weeks), very PTB (28-<32 weeks), moderate PTB (32-<34 weeks) and late PTB (34-<37 weeks) respectively.

Main results

The main results are shown in Table 3. Pregnancy non-varying variables that increased the risk of spontaneous preterm were maternal age less than 18 (ARR=1.13, 95% CI: 1.02-1.26); being Muslim compared to Brahmin and Chhetri (1.53, 1.16-2.01); first pregnancy as compared to parity 1 to 4 (1.15, 1.04-1.28); having a multiple birth (4.91, 4.20-5.75) and having a male child (1.10, 1.02-1.17). Pregnancy non-varying variables that decreased the risk of spontaneous preterm were maternal education of more than 5 years (0.81, 0.73-0.90); maternal height of >=150 cm (0.89, 0.81-0.98) and being wealthier: richer (0.83, 0.74-0.93) wealth quintile compared to the poorest wealth quintile. Pregnancy non-varying variables that showed no association with spontaneous preterm births in the bivariable/unadjusted models are any prior pregnancy ending in SB, any prior pregnancy ending in multiples, and any prior pregnancy ending in miscarriage, and interpregnancy interval. The pregnancy non-varying variable that showed an association in the bivariable model, but not in the multivariable models was any prior pregnancy ending in death for a live birth.

For morbidity symptoms, some increased the risk of preterm, and all of these showed increased risk when symptoms were present in the 3rd trimester. Having vaginal bleeding (ARR= 1.53, 95% CI: 1.08-2.18); swelling (1.37, 1.17-1.60); high systolic BP (1.47, 1.08-2.01) and high diastolic BP (1.41, 1.17-1.70) in the 3rd trimester significantly increased the risk of spontaneous preterm. Some symptom variables significantly decreased the risk of spontaneous preterm. Having respiratory problem in the 3rd trimester (0.86, 0.79-0.94); and having poor appetite, nausea and vomiting in the 2nd trimester (0.86, 0.80-0.92) and in the 3rd trimester (0.86, 0.79-0.94) decreased the risk of spontaneous preterm. Symptom variables that showed no association with spontaneous preterm were STI and GI problems. Symptom variables that were

For peer review only

significant in the bivariable model, but not significant in the multivariable models were swelling in the 2nd trimester and diastolic blood pressure in the 2nd trimester. For maternal weight, higher weight gain from the 2nd to the 3rd trimester was associated with a decreased the risk of spontaneous preterm (0.89, 0.87-0.90).

To examine the possible bias associated with exclusion of pregnancies with missing data, we compared characteristics of women excluded in the regression analysis (n=9,461) (mainly because of missing morbidity in 2nd trimester due to late enrollment) with those included in the regression analysis (n=21,297) (supplementary Table S1).The women excluded in the regression analysis were slightly better off than those included in the regression based on education and socioeconomic status but most relevant, the spontaneous preterm prevalence was 17.9% for those excluded in the regression compared to 13.8% included in the regression.

We also reran the regression model including number of ANC visits. The fewer the number of ANC visits, the higher the risk of spontaneous preterm birth (Table S2). The other regression coefficients did not change in any qualitative way. This could be due to fewer ANC visits putting women at higher risk for spontaneous preterm birth as services provided in ANC (counseling, iron folic acid tablets, blood pressure and weight measurements) are provided less often, but this association may also be due to a shorter duration of pregnancy leading to less time available for ANC visits.

Table 3: Crude and Adjusted Risk Ratios for associations between risk factors and spontaneous preterm birth

**Name of Variables**	**Categories**	**Unadjusted Model**	**Adjusted Model (N=21,297)**
		Risk Ratio (95% CI)	Risk Ratio (95% CI)
Maternal Age at LMP	18 to 35	1	1
	Less than 18	1.19*** [1.11,1.28]	1.13* [1.02,1.26]
	More than 35	1.57*** [1.36,1.81]	1.22 [0.98,1.51]
Caste/Religion Categories	Brahmin and Chhetri	1	1
	Vaishya	1.33** [1.09,1.62]	1.23 [0.95,1.59]
	Shudra	1.55*** [1.26,1.90]	1.23 [0.94,1.62]
	Muslim and others	1.96*** [1.60,2.42]	1.53** [1.16,2.01]
Mother's Years of Education	No schooling	1	1
	1 to 5 years	0.86** [0.78,0.95]	0.91 [0.80,1.03]
	More than 5 years	0.71*** [0.66,0.76]	0.81*** [0.73,0.90]
Quintiles of Wealth	Poorest	1	1
	Poor	0.86*** [0.79,0.93]	0.90* [0.82,1.00]
	Middle	0.89** [0.82,0.96]	0.95 [0.86,1.05]

For peer review only - http://bmjopen.bmj.com/site/about/guidelines.xhtml

**Name of Variables**	**Categories**	**Unadjusted Model**<br>Risk Ratio (95% CI)	**Adjusted Model (N=21,297)**<br>Risk Ratio (95% CI)
	Richer	0.73*** [0.67,0.79]	0.83** [0.74,0.93]
	Richest	0.71*** [0.65,0.77]	0.88* [0.78,1.00]
Mother's height(centimeter)	<145	1	1
	145-<150	0.93 [0.86,1.01]	0.98 [0.88,1.08]
	>=150	0.81*** [0.75,0.87]	0.89* [0.81,0.98]
Parity including both LB and SB, at Enrollment	Parity 1 to 4	1	1
	More than 4	1.32*** [1.17,1.48]	1.17 [0.99,1.37]
	Prior Pregnant but parity 0	1.02 [0.85,1.22]	0.92 [0.62,1.37]
	No Prior Pregnant	1.10** [1.04,1.17]	1.15** [1.04,1.28]
Interpregnancy Intervals	18 to 36 months	1	1
	Less than 18 months	1.07 [0.99,1.14]	1.08 [0.99,1.18]
	More than 36 months	0.98 [0.89,1.09]	0.9 [0.79,1.02]
	No Prior Pregnancy	1.11** [1.03,1.20]	1 [1.00,1.00]
Any death among prior LB	Prior LB but not died	1	1
	Prior LB died	1.19*** [1.09,1.29]	1.07 [0.97,1.19]
	Prior Pregnancy but no LB	1.07 [0.92,1.25]	1.06 [0.75,1.49]
	No prior pregnancy	1.12*** [1.06,1.19]	1 [1.00,1.00]
Any prior pregnancy ended in SB	Prior Pregnancy but no SB	1	
	Prior SB	1.08 [0.94,1.23]	
	No Prior Pregnancy	1.08** [1.02,1.15]	
Any prior pregnancy ended in miscarriage	Prior Pregnancy but no miscarriage	1	
	Prior miscarriage	0.94 [0.86,1.03]	
	No Prior Pregnancy	1.07* [1.01,1.13]	
Any prior pregnancy ended in multiples	Prior Pregnancy but no multiples	1	
	Prior multiples	1.14 [0.87,1.49]	
	No prior pregnancy	1.08** [1.02,1.14]	
Multiple Birth	Singleton	1	1
	Twin/Triplet	3.92*** [3.52,4.38]	4.91*** [4.20,5.75]
Sex of the child	Female	1	1
	Male	1.10*** [1.04,1.17]	1.10** [1.02,1.17]
	Twin/Triplet	4.13*** [3.69,4.63]	1
STI in at least one visit of 2nd trimester?	No	1	
	Yes	0.99 [0.92,1.07]	
STI in at least one visit of 3rd trimester?	No	1	
	Yes	1.01 [0.92,1.12]	
Respiratory Problems in at least one visit of 2nd trimester?	No	1	1
	Yes	1 [0.94,1.06]	1.08 [1.00,1.16]
Respiratory Problems in at least one visit of 3rd trimester?	No	1	1
	Yes	0.85*** [0.79,0.92]	0.86** [0.79,0.94]
GI Problems in at least one visit of 2nd trimester?	No	1	
	Yes	1.08 [0.98,1.18]	
GI Problems in at least one visit of 3rd trimester?	No	1	
	Yes	1.04 [0.94,1.16]	
Poor appetite, nausea & vomiting in at least one visit of 2nd trimester?	No	1	1
	Yes	0.81*** [0.77,0.86]	0.86*** [0.80,0.92]
Poor appetite, nausea & vomiting in at least	No	1	1

For peer review only

Name of Variables	Categories	Unadjusted Model<br>Risk Ratio (95% CI)	Adjusted Model (N=21,297)<br>Risk Ratio (95% CI)
one visit of 3rd trimester?	Yes	0.88** [0.82,0.95]	0.86*** [0.79,0.94]
Vaginal Bleeding in at least one visit of 2nd trimester?	No	1	1
	Yes	0.91 [0.71,1.17]	0.84 [0.71,1.17]
Vaginal Bleeding in at least one visit of 3rd trimester?	No	1	1
	Yes	1.44* [1.05,1.98]	1.53*[1.08,2.18]
Swelling in at least one visit of 2nd trimester?	No	1	1
	Yes	1.32*** [1.12,1.55]	1.19 [0.98,1.46]
Swelling in at least one visit of 3rd trimester?	No	1	1
	Yes	1.25** [1.09,1.44]	1.37*** [1.17,1.60]
High Systolic BP in 2nd trimester?	Normal Systolic BP	1	1
	High Systolic BP	0.89 [0.59,1.34]	0.67 [0.40,1.12]
High Systolic BP in 3rd trimester?	Normal Systolic BP	1	1
	High Systolic BP	1.92*** [1.52,2.41]	1.47* [1.08,2.01]
High diastolic BP in 2nd trimester?	Normal diastolic BP	1	1
	High diastolic BP	1.34** [1.12,1.60]	1.09 [0.85,1.40]
High diastolic BP in 3rd trimester?	Normal diastolic BP	1	1
	High diastolic BP	1.57*** [1.37,1.80]	1.41*** [1.17,1.70]
Average weight in 3rd trimester minus Average weight in 2nd trimester (kg)		0.88*** [0.87,0.90]	0.89*** [0.87,0.90]

* $p < 0.05$, ** $p < 0.01$, *** $p < 0.001$

For peer review only

For peer review only - http://bmjopen.bmj.com/site/about/guidelines.xhtml

DISCUSSION

Our study is one of the only large-scale studies on preterm births using data from an existing pregnancy surveillance in rural Sarlahi, Nepal. The prevalence of preterm birth is 15%, higher than previous estimates from Nepal [18, 19] which were primarily from urban areas and large hospital-based studies. Our study's strength is that it was population-based and included all home and facility deliveries but is confined to a rural and relatively small geographic area (one third of a district). Our study population is not necessarily representative of all of Nepal, but it is representative of Province 2 in the Terai region within which Sarlahi district is located. For example, the NMR in our study was 31 per 1000 live births. This is similar to the NMR in the 2016 Nepal Demographic Health Survey (NDHS) for Province 2 (30 per 1000). Similarly, 67% of women in our study had no schooling, slightly higher than the 61% in the NDHS for Province 2. NDHS did not provide data on ANC 4+ for Province 2 but rural areas of Nepal had 62% coverage of ANC 4+. It should be noted in our study that health care seeking in pregnancy is low considering the low rates of 4 or more ANC visits (28%) and facility deliveries (38%). The low rates of induction and caesarean section point to a very low proportion of the PTBs being due to iatrogenic causes.

For peer review only

In many other settings, both younger and older maternal age have been reported to be risk factors for preterm birth. [24-30] Being from Muslim caste was positively associated with preterm as compared to Brahmin/Chhetri, which constitutes the major caste in Nepal. Caste/religion is a social construction, and studies in different places have shown that women in minor caste/race/color have higher risk of preterm births. [31-33] It significantly matters what position an individual holds within a society , with regards to occurrence of diseases and also their unequal distribution.[34-36]. First pregnancy (primipara) has been shown to be associated with spontaneous preterm birth in other studies. A study in France showed that primipara as compared to parity 2-3 increased the risk of preterm birth by 1.8 times.[37] Another study in the USA showed that being primipara as compared to multipara increased the risk of very preterm and extremely preterm birth, with the highest risk of 1.37 times for extremely preterm birth.[38] Meta-analysis done using 14 cohort studies from LMICs [39] and a study from sub-saharan African countries [40] also show that primiparity is associated with increased odds of preterm birth. Primipara is a risk factor for hypertensive disorders of pregnancy (HDP), which increases the risk of preterm birth.[41] Our study did not show interpregnancy interval to be the risk factor for spontaneous preterm birth. However, other

studies on relationships between interpregnancy interval and preterm birth consistently showed that shorter interpregnancy intervals increase the risk of preterm births. However, the intervals used were not uniform across studies. One study found that, compared to an IPI of 18–23 months, IPIs <3, 3–5, and 6–12 months had higher risks for preterm birth.[42] Another study with median IPI of 36 months showed that, compared to an IPI of 24–36 months, an IPI of <24 months was associated with preterm delivery.[43] Different studies corroborate our finding that multiple births are a risk factor for preterm birth. [18, 44, 45] Similar to our study, others also found male children at higher risk of being preterm[46-48], but a study in Nepal found that female children had a higher risk of being preterm.[18] This study in Nepal enrolled live births in a hospital setting, and had almost half the prevalence of our study. [18] They could have missed more males that had preterm births at home or on the way to a facility.

Different studies in Nepal [18] and outside of Nepal [49-51] have also shown that higher education of mothers decreases the risk of preterm births. Higher education of mothers can lead to increased knowledge and awareness regarding pregnancy-related care and thus decrease adverse outcomes of pregnancy. We found greater maternal height to be protective for spontaneous preterm birth, similar to the findings from a meta-analysis done using 12 cohort studies from LMICs. [52] .We found that women in the richer wealth quintile had a lower risk of spontaneous preterm births. Having higher household economic status probably does not directly affect the gestational age at outcome, instead, it probably is mediated by factors like nutrition, physically demanding work during pregnancy, type of care at home, stress level and other psychological factors.[53]

Pregnancy-varying morbidities that significantly decreased the risk of preterm birth in our analysis were respiratory problems in the 3rd trimester; and poor appetite, nausea and vomiting in the 2nd trimester, and the 3rd trimester. On segregating the symptoms within respiratory problems , we found that it was the persistent cough in the 3rd trimester that decreased the risk of preterm. A similar relationship was found between persistent cough and Large for Gestational Age (LGA) in another study done using the same data as ours. [54] However, we could not find any such association in the previous literature. The association might be due to some unmeasured confounders. Or it could be that women with persistent cough in the 3rd trimester made more frequent check-up visits. We saw that 40% of women with persistent cough in the 3rd trimester sought treatment for cough, and almost all had sought treatment

For peer review only - http://bmjopen.bmj.com/site/about/guidelines.xhtml

more than once. The pathogenesis of nausea and vomiting in pregnancy is not very clear, but it is broadly accepted to be multifactorial, with the involvement of genetic, endocrine, and gastrointestinal factors. [55] Our findings corroborate with previous findings that nausea and vomiting is associated with reduced risk of preterm birth. [56-59] Specifying by trimesters, a study by Wallin et. al. in Nepal showed similar findings - poor appetite, nausea and vomiting in first trimester was not significantly associated with spontaneous preterm births, but having these symptoms in the 2nd trimester decreased the risk of spontaneous preterm by 25%.[60]

Pregnancy-varying morbidities that significantly increased the risk of spontaneous preterm birth were vaginal bleeding, swelling of hands and face, high diastolic and systolic BP, all in the 3rd trimester. Other studies show similar results for vaginal bleeding. Vaginal bleeding is associated with fetal exposure to oral pathogens, which thereby increases the risk of spontaneous preterm birth, however, whether bleeding is the cause or result of fetal exposure to oral pathogens is not clear.[61] A prospective cohort study, separating first and second trimesters showed that vaginal bleeding in both trimesters increased the risk of preterm birth by 3.6 times, while bleeding in the second trimester only, was not associated with preterm birth.[62] A systematic review using 23 studies showed that bleeding in early pregnancy increased the risk of preterm births.[63] A study in China showed that vaginal bleeding in the first-trimester increased the risk of preterm births, and the severity, duration and initial timing of vaginal bleeding had different effects on the severity of preterm births.[61] Due to the low enrollment of women in the 1st trimester, we could not look at the association of vaginal bleeding in the 1st trimester with spontaneous preterm birth. However, all of the above information indicates that vaginal bleeding can be an important predictor of spontaneous preterm birth and health care workers should recommend appropriate interventions for women if they present with vaginal bleeding (such as more frequent follow up or referral for higher level care).

Other studies on blood pressure during pregnancy have also shown that a rise in systolic BP (over 30 mm Hg) or diastolic BP (over 15mm Hg), from early pregnancy to the mid third trimester significantly increased the risk of spontaneous preterm birth by 2 to 3 times.[64] Another study showed that an increase in 10 mm Hg in diastolic BP increased the risk of preterm birth by 29%. [65] These indicate the importance of measuring BP during the 3rd trimester. High BP in the 3rd trimester is an indicator of pre-eclampsia/eclampsia and can

For peer review only - http://bmjopen.bmj.com/site/about/guidelines.xhtml

predict preterm birth. Measuring BP frequently and monitoring the rise and cause of increased BP is important for predicting spontaneous preterm birth.

For maternal weight, higher weight gain from the 2nd to the 3rd trimester decreased the risk of spontaneous preterm birth. This is consistent with a study done outside Nepal, which showed that very low weight gain was strongly associated with very preterm delivery, and that this varied by pre-pregnancy BMI, where underweight women had the highest association and very obese women had lowest association with preterm.[66] Our study was conducted is a non-obese and undernourished population. We do not have pre-pregnancy BMI, so we looked at the mean BMI in the first trimester. Though the first trimester represents less half of the pregnancies in the study, it hints at undernutrition in the population. The mean BMI was 19.1 kg/m^2 , and 37% had BMI less than 18.5 kg/m^2. So, less maternal weight gain in such population can pose a risk to spontaneous preterm births. Given spontaneous preterm births have shorter gestation, the increase in weight gain will likely be less because there is less time to increase weight, especially in the third trimester, when much of the gestational weight is gained.

Strengths and Limitations

This was a large population-based study that was generally representative of the rural Terai region of Nepal. Multiple variables were collected, including socioeconomic, demographic, pregnancy history, and monthly morbidity in pregnancy that could be examined as risk factors for spontaneous preterm birth. Although there was some missing data in regression analyses, a comparison of those with and without missing data did not show large differences in risk factor prevalence. However, those missing data had higher prevalence of preterm birth. It is possible that if women with missing data were included in the regression, we may have seen stronger associations but the potential bias of these differences is unclear. Gestational age (GA) at birth was measured using date of last menstrual period (LMP) as usually done in the LMICs rather than by ultrasound. However, as LMP was asked at enrollment which was generally early in pregnancy, there is less recall bias than LMP recalled at delivery or late in pregnancy. Using the same method as we used to obtain LMP, Gernand et al. found that LMP based estimates of GA in rural Bangladesh were a mean 2.8 days longer than what was obtained on ultrasound. [67] We therefore believe that this is probably not a significant limitation. Women were

For peer review only - http://bmjopen.bmj.com/site/about/guidelines.xhtml

followed prospectively at monthly intervals to reduce recall bias about pregnancy morbidities and symptoms. In order to reduce misclassification of stillbirths and live births, women were asked whether the infant moved, breathed or cried after birth.

Some variables associated with increased risk of spontaneous preterm births in previous studies, for example, a prior pregnancy ending in a preterm birth, gestational diabetes , maternal anemia and pre-pregnancy maternal nutritional status were not measured in the main trial. However, other important morbidity variables were measured and used in the analysis. Some covariates were highly correlated with each other (such as some reproductive history ones) and so, not all were included in the multivariable regression. Some covariates were not statistically significant in unadjusted analyses and there was not a compelling biological or sociological reason to include them in the adjusted model. Other important variables like smoking and alcohol, although measured, could not be included in the final regression analysis as their prevalence was very low in this population. We believe these risk factors are likely generalizable for similar populations in South Asia.

CONCLUSION

Preterm birth is a leading risk factor for neonatal and under-5 mortality and morbidity worldwide. To reduce neonatal mortality, preventing preterm births can be a vital step. Some of the risk factors from our study are amenable to antenatal interventions but many others need more understanding of the underlying causal mechanisms. Maternal education and awareness can play a role in the long term, while good quality antenatal care, as suggested by the new WHO recommendation of 8 contacts during pregnancy, may help reduce some PTBs. Future research should focus on basic research involving the field of 'omics' using biological samples and implementation research to improve antenatal care and maternal nutrition.

OTHER INFORMATION

Authors' contribution:

SS, EH, DM, SZ, REB and JK conceptualized and designed the analysis. SS conducted the analysis and wrote the manuscript. LCM, JMT, SKK, SLC and JK were investigators in the parent trial. All authors reviewed results, analysis, discussed interpretations, and contributed to development and revision of the manuscript.

For peer review only - http://bmjopen.bmj.com/site/about/guidelines.xhtml

Competing interests:

The authors declare that they have no competing interests.

Funding source:

NOMS was supported by the National Institutes for Child Health and Development (HD060712) and the Bill & Melinda Gates Foundation (OPP1084399). The analysis for this paper was funded by the National Institutes for Child Health and Development (HD092411). The funders did not have a role in the design of the study, the data collection, nor the analysis, interpretation and writing of the manuscript.

Availability of data and materials:

No data are available.

Consent for publication:

No individual information including data, images and video are included in this manuscript.

Ethics approval and consent to participate:

NOMS was approved by the institutional review board (IRB) of the Johns Hopkins Bloomberg School of Public Health in the USA and by the IRB of the Institute of Medicine, Tribhuvan University, Kathmandu, Nepal. This analysis of secondary data was considered exempt by the Johns Hopkins Bloomberg School of Public Health institutional review board (IRB) (FWA00000287). Verbal consent was obtained from women for their participation and their infants for the primary data collection.

Acknowledgement:

We would like to acknowledge study staff and all participants at Nepal Nutritional Intervention Project-Sarlahi site. We would also like to give special thanks to Dr. Leah Horton at Johns Hopkins Bloomberg School of Public Health for her technical contributions during data management.

For peer review only

REFERENCES:

1 Dbstet A. WHO: recommended definitions, terminology and format for statistical tables related to the perinatal period and use of a new certificate for cause of perinatal deaths, *Acta Obstet Gynecol Scand* 1977;56:247-53.

2 Blencowe H, Cousens S, Oestergaard MZ, et al. National, regional, and worldwide estimates of preterm birth rates in the year 2010 with time trends since 1990 for selected countries: a systematic analysis and implications, *The lancet* 2012;379:2162-72.

3 Perin J, Mulick A, Yeung D, et al. Global, regional, and national causes of under-5 mortality in 2000–19: an updated systematic analysis with implications for the Sustainable Development Goals, *The Lancet Child & Adolescent Health* 2022;6:106-15.

4 Chawanpaiboon S, Vogel JP, Moller A, et al. Global, regional, and national estimates of levels of preterm birth in 2014: a systematic review and modelling analysis, *The Lancet Global Health* 2019;7:e37-46.

5 Araújo BFd, Zatti H, Madi JM, et al. Analysis of neonatal morbidity and mortality in late-preterm newborn infants, *J Pediatr* 2012;88:259-66.

6 Mwaniki MK, Atieno M, Lawn JE, et al. Long-term neurodevelopmental outcomes after intrauterine and neonatal insults: a systematic review, *The Lancet* 2012;379:445-52.

7 Vogel JP, Chawanpaiboon S, Moller A, et al. Best Practice & Research Clinical Obstetrics and Gynaecology, *Best Practice & Research Clinical Obstetrics and Gynaecology* 2018;30:1e10.

8 Orchinik LJ, Taylor HG, Espy KA, et al. Cognitive outcomes for extremely preterm/extremely low birth weight children in kindergarten, *Journal of the International Neuropsychological Society* 2011;17:1067-79.

9 Johnson S, Evans TA, Draper ES, et al. Neurodevelopmental outcomes following late and moderate prematurity: a population-based cohort study, *Archives of Disease in Childhood-Fetal and Neonatal Edition* 2015;100:F301-8.

For peer review only

For peer review only - http://bmjopen.bmj.com/site/about/guidelines.xhtml

10 Moreira RS, Magalhães LC, Alves CR. Effect of preterm birth on motor development, behavior, and school performance of school-age children: a systematic review☆,☆☆, *J Pediatr* 2014;90:119-34.

11 Petrou S, Abangma G, Johnson S, et al. Costs and health utilities associated with extremely preterm birth: evidence from the EPICure study, *Value in Health* 2009;12:1124-34.

12 Singer LT, Salvator A, Guo S, et al. Maternal psychological distress and parenting stress after the birth of a very low-birth-weight infant, *JAMA* 1999;281:799-805.

13 Family Health Division of the Department of Health Services of the Ministry of Health/Nepal, New ERA/Nepal, Macro O. Nepal Demographic and Health Survey 2001. 2002.

14 Ministry of Health - MOH/Nepal, New ERA/Nepal, ICF. Nepal Demographic and Health Survey 2016. 2017.

15 Ministry of Health and Population - MOHP/Nepal, New ERA/Nepal, International M. Nepal Demographic And Health Survey 2006. 2007.

16 Vogel JP, Chawanpaiboon S, Moller AB, et al. The global epidemiology of preterm birth, *Best Pract Res Clin Obstet Gynaecol* 2018;52:3-12 doi:S1521-6934(18)30079-8 [pii] [published Online First: Oct].

17 National Planning Commission. Sustainable Development Goals 2016–2030: National (Preliminary) Report, *Kathmandu: National Planning Commission* 2015.

18 Gurung A, Wrammert J, Sunny AK, et al. Incidence, risk factors and consequences of preterm birth–findings from a multi-centric observational study for 14 months in Nepal, *Archives of Public Health* 2020;78:1-9.

19 Ashish KC, Wrammert J, Nelin V, et al. Level of mortality risk for babies born preterm or with a small weight for gestation in a tertiary hospital of Nepal, *BMC Public Health* 2015;15:1-9.

20 Anonymous . Nepal: share of rural population. ;2021.

21 Bryce E, Gurung S, Tong H, et al. Population attributable fractions for risk factors for spontaneous preterm births in 81 low-and middle-income countries: A systematic analysis, *Journal of global health* 2022;12.

22 Dahal DR. Social composition of the population: caste/ethnicity and religion in Nepal, *Population monograph of Nepal* 2003;1:87-135.

23 Rutstein SO, Johnson K. The DHS wealth index. DHS comparative reports no. 6, *Calverton: ORC Macro* 2004.

24 Wagura P, Wasunna A, Laving A, et al. Prevalence and factors associated with preterm birth at kenyatta national hospital, *BMC pregnancy and childbirth* 2018;18:1-8.

For peer review only

25 Fuchs F, Monet B, Ducruet T, et al. Effect of maternal age on the risk of preterm birth: A large cohort study, *PloS one* 2018;13:e0191002.

26 Weng Y, Yang C, Chiu Y. Risk assessment of adverse birth outcomes in relation to maternal age, *PloS one* 2014;9:e114843.

27 Muhihi A, Sudfeld CR, Smith ER, et al. Risk factors for small-for-gestational-age and preterm births among 19,269 Tanzanian newborns, *BMC pregnancy and childbirth* 2016;16:1-12.

28 Anderson C, Smitherman AB, Engel SM, et al. Modifiable and non-modifiable risk factors for preterm delivery among adolescent and young adult cancer survivors, *Cancer Causes & Control* 2018;29:289-95.

29 Leonard SA, Crespi CM, Gee DC, et al. Prepregnancy risk factors for preterm birth and the role of maternal nativity in a low-income, Hispanic population, *Matern Child Health J* 2015;19:2295-302.

30 Kildea SV, Gao Y, Rolfe M, et al. Risk factors for preterm, low birthweight and small for gestational age births among Aboriginal women from remote communities in Northern Australia, *Women and Birth* 2017;30:398-405.

31 Menon R, Dunlop AL, Kramer MR, et al. An overview of racial disparities in preterm birth rates: caused by infection or inflammatory response? *Acta Obstet Gynecol Scand* 2011;90:1325-31.

32 Kramer MR, Hogue CJ, Dunlop AL, et al. Preconceptional stress and racial disparities in preterm birth: an overview, *Acta Obstet Gynecol Scand* 2011;90:1307-16.

33 Patel RR, Steer P, Doyle P, et al. Does gestation vary by ethnic group? A London-based study of over 122 000 pregnancies with spontaneous onset of labour, *Int J Epidemiol* 2004;33:107-13.

34 Goodman AH. Why genes don't count (for racial differences in health). *Am J Public Health* 2000;90:1699.

35 Krieger N. A glossary for social epidemiology, *Journal of Epidemiology & Community Health* 2001;55:693-700.

36 Wilkinson RG, Marmot M. Social determinants of health: the solid facts: World Health Organization 2003.

37 Delnord M, Blondel B, Prunet C, et al. Are risk factors for preterm and early-term live singleton birth the same? A population-based study in France, *BMJ open* 2018;8:e018745.

38 Salihu H, Mbah AK, Alio AP, et al. Nulliparity and preterm birth in the era of obesity epidemic, *The journal of maternal-fetal & neonatal medicine* 2010;23:1444-50.

For peer review only

39 Kozuki N, Lee AC, Silveira MF, et al. The associations of parity and maternal age with small-for-gestational-age, preterm, and neonatal and infant mortality: a meta-analysis, *BMC Public Health* 2013;13:1-10.

40 Alamneh TS, Teshale AB, Worku MG, et al. Preterm birth and its associated factors among reproductive aged women in sub-Saharan Africa: evidence from the recent demographic and health surveys of sub-Sharan African countries, *BMC pregnancy and childbirth* 2021;21:1-11.

41 Ye C, Ruan Y, Zou L, et al. The 2011 survey on hypertensive disorders of pregnancy (HDP) in China: prevalence, risk factors, complications, pregnancy and perinatal outcomes, *PloS one* 2014;9:e100180.

42 Nerlander LM, Callaghan WM, Smith RA, et al. Short interpregnancy interval associated with preterm birth in U S adolescents, *Matern Child Health J* 2015;19:850-8 doi:10.1007/s10995-014-1583-z [doi] [published Online First: Apr].

43 Mahande MJ, Obure J. Effect of interpregnancy interval on adverse pregnancy outcomes in northern Tanzania: a registry-based retrospective cohort study, *BMC pregnancy and childbirth* 2016;16:1-9.

44 Kim J, Cho M, Choi G. Multiple factors in the second trimester of pregnancy on preterm labor symptoms and preterm birth, *Journal of Korean Academy of Nursing* 2017;47:357-66.

45 Shah R, Mullany LC, Darmstadt GL, et al. Incidence and risk factors of preterm birth in a rural Bangladeshi cohort, *BMC pediatrics* 2014;14:1-11.

46 Peelen MJ, Kazemier BM, Ravelli AC, et al. Impact of fetal gender on the risk of preterm birth, a national cohort study, *Acta Obstet Gynecol Scand* 2016;95:1034-41.

47 Zeitlin J, Ancel P, Larroque B, et al. Fetal sex and indicated very preterm birth: results of the EPIPAGE study, *Obstet Gynecol* 2004;190:1322-5 doi:https://doi.org/10.1016/j.ajog.2003.10.703.

48 Lao TT, Sahota DS, Suen SS, et al. The impact of fetal gender on preterm birth in a southern Chinese population, *The Journal of Maternal-Fetal & Neonatal Medicine* 2011;24:1440-3.

49 Poulsen G, Strandberg-Larsen K, Mortensen L, et al. Exploring Educational Disparities in Risk of Preterm Delivery: A Comparative Study of 12 E uropean Birth Cohorts, *Paediatr Perinat Epidemiol* 2015;29:172-83.

50 Oftedal A, Busterud K, Irgens LM, et al. Socio-economic risk factors for preterm birth in Norway 1999–2009, *Scand J Public Health* 2016;44:587-92.

51 Auger N, Leduc L, Naimi AI, et al. Delivery at term: impact of university education by week of gestation, *Journal of Obstetrics and Gynaecology Canada* 2016;38:118-24.

For peer review only

52 Anonymous . Short maternal stature increases risk of small-for-gestational-age and preterm births in low-and middle-income countries: individual participant data meta-analysis and population attributable fraction, *J Nutr* 2015;145:2542-50.

53 Kramer MS, Goulet L, Lydon J, et al. Socio-economic disparities in preterm birth: causal pathways and mechanisms, *Paediatr Perinat Epidemiol* 2001;15:104-23.

54 Hazel EA, Mohan D, Zeger S, et al. Demographic, socio-economic, obstetric, and behavioral factors associated with small-and large-for-gestational-age from a prospective, population-based pregnancy cohort in rural Nepal: a secondary data analysis, *BMC Pregnancy and Childbirth* 2022;22:1-13.

55 Bustos M, Venkataramanan R, Caritis S. Nausea and vomiting of pregnancy - What's new? *Auton Neurosci* 2017;202:62-72 doi:S1566-0702(16)30048-0 [pii] [published Online First: Jan].

56 Tierson FD, Olsen CL, Hook EB. Nausea and vomiting of pregnancy and association with pregnancy outcome, *Obstet Gynecol* 1986;155:1017-22.

57 Petitti DB. Nausea and pregnancy outcome, *Birth* 1986;13:223-6.

58 Koren G, Clark S, Hankins GD, et al. Maternal safety of the delayed-release doxylamine and pyridoxine combination for nausea and vomiting of pregnancy; a randomized placebo controlled trial, *BMC pregnancy and childbirth* 2015;15:1-6.

59 Klebanoff MA, Koslowe PA, Kaslow R, et al. Epidemiology of vomiting in early pregnancy. *Obstet Gynecol* 1985;66:612-6.

60 Wallin AR, Tielsch JM, Khatry SK, et al. Nausea, vomiting and poor appetite during pregnancy and adverse birth outcomes in rural Nepal: an observational cohort study, *BMC pregnancy and childbirth* 2020;20:1-12.

61 Boggess KA, Moss K, Murtha A, et al. Antepartum vaginal bleeding, fetal exposure to oral pathogens, and risk for preterm birth at *Am J Obstet Gynecol* 2006;194:954-60 doi:S0002-9378(06)00232-8 [pii] [published Online First: Apr].

62 Yang J, Hartmann KE, Savitz DA, et al. Vaginal bleeding during pregnancy and preterm birth, *Am J Epidemiol* 2004;160:118-25 doi:160/2/118 [pii] [published Online First: Jul 15].

63 Hackney DN, Glantz JC. Vaginal bleeding in early pregnancy and preterm birth: systemic review and analysis of heterogeneity, *J Matern Fetal Neonatal Med* 2011;24:778-86 doi:10.3109/14767058.2010.530707 [doi] [published Online First: Jun].

64 Zhang J, Villar J, Sun W, et al. Blood pressure dynamics during pregnancy and spontaneous preterm birth, *Obstet Gynecol* 2007;197:162. e1,162. e6.

65 Chatzi L, Plana E, Daraki V, et al. Metabolic syndrome in early pregnancy and risk of preterm birth, *Am J Epidemiol* 2009;170:829-36.

For peer review only

66 Dietz PM, Callaghan WM, Cogswell ME, et al. Combined effects of prepregnancy body mass index and weight gain during pregnancy on the risk of preterm delivery, *Epidemiology* 2006;17:170-7 doi:00001648-200603000-00011 [pii] [published Online First: Mar].

67 Gernand AD, Paul RR, Ullah B, et al. A home calendar and recall method of last menstrual period for estimating gestational age in rural Bangladesh: a validation study, *Journal of Health, Population and Nutrition* 2016;35:1-9.

For peer review only

Figure 1- Flow Diagram of Participants

Figure 1: Flow Diagram for Participants

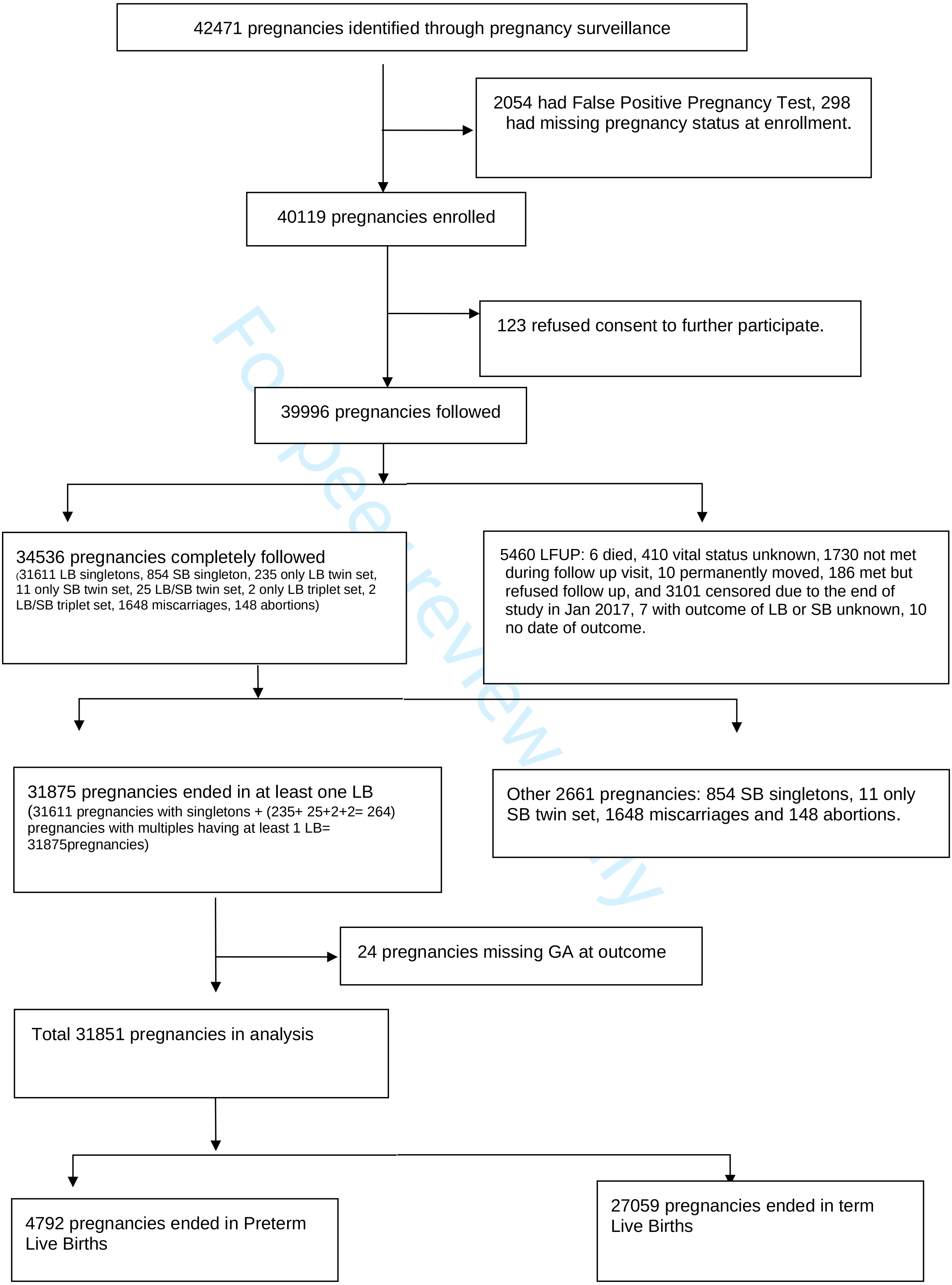

SUPPLEMENTARY TABLES

Table S1. Comparing Pregnancy non-varying Variables by pregnancies Included and Excluded in the Regression Analysis

**Variables**	**Categories**	**Total** N=30,758	**Included in regression** N=21,297	**Excluded in regression** N=9,461	**p-value**
		**N (%)**	**N (%)**	**N (%)**	
Maternal Age at LMP	18 to 35	25,300 (82.3)	17,683 (83.0)	7,617 (80.5)	<0.001
	Less than 18	4,792 (15.6)	3,169 (14.9)	1,623 (17.2)	
	More than 35	666 (2.2)	445 (2.1)	221 (2.3)	
Caste/Ethnicity Categories	Brahmin and Chhetri	879 (2.9)	661 (3.1)	218 (2.3)	<0.001
	Vaishya	22,104 (71.9)	15,412 (72.4)	6,692 (70.7)	
	Shudra	4,826 (15.7)	3,392 (15.9)	1,434 (15.2)	
	Muslim and others	2,919 (9.5)	1,832 (8.6)	1,087 (11.5)	
	Missing	30 (0.1)	0 (0.0)	30 (0.3)	
Mother's Education	No schooling	20,891 (67.9)	14,561 (68.4)	6,330 (66.9)	0.032
	1 to 5 years	2,613 (8.5)	1,819 (8.5)	794 (8.4)	
	More than 5 years	7,224 (23.5)	4,917 (23.1)	2,307 (24.4)	
	Missing	30 (0.1)	0 (0.0)	30 (0.3)	
Quintiles of Wealth	Poorest	6,354 (20.7)	4,414 (20.7)	1,940 (20.5)	0.004
	Poorer	6,210 (20.2)	4,386 (20.6)	1,824 (19.3)	
	Middle	6,152 (20.0)	4,289 (20.1)	1,863 (19.7)	
	Richer	6,036 (19.6)	4,172 (19.6)	1,864 (19.7)	
	Richest	5,985 (19.5)	4,036 (19.0)	1,949 (20.6)	
	Missing	21 (0.1)	0 (0.0)	21 (0.2)	
Maternal Height (centimeter)	<145	4,510 (14.7)	3,193 (15.0)	1,317 (13.9)	0.042
	145-<150	9,227 (30.0)	6,413 (30.1)	2,814 (29.7)	
	>=150	16,974 (55.2)	11,691 (54.9)	5,283 (55.8)	
	Missing	47 (0.2)	0 (0.0)	47 (0.5)	
Parity including both LB and SB, at Enrollment	Parity 1 to 4	19,805 (64.4)	14,137 (66.4)	5,668 (59.9)	<0.001
	More than 4	1,351 (4.4)	875 (4.1)	476 (5.0)	
	Prior Pregnant but parity 0	723 (2.4)	515 (2.4)	208 (2.2)	
	No Prior Pregnant	8,717 (28.3)	5,770 (27.1)	2,947 (31.1)	
	Missing	162 (0.5)	0 (0.0)	162 (1.7)	
Interpregnancy Interval based on maternal recall	18 to 36 months	7,723 (25.1)	5,540 (26.0)	2,183 (23.1)	<0.001
	Less than 18 months	11,201 (36.4)	7,693 (36.1)	3,508 (37.1)	
	More than 36 months	3,106 (10.1)	2,294 (10.8)	812 (8.6)	
	No Prior Pregnancy	8,717 (28.3)	5,770 (27.1)	2,947 (31.1)	
	Missing	11 (0.0)	0 (0.0)	11 (0.1)	
Any deaths among Prior LB	Prior LB but not died	17,089 (55.6)	12,273 (57.6)	4,816 (50.9)	<0.001
	Prior LB died	3,518 (11.4)	2,555 (12.0)	963 (10.2)	
	Prior Pregnancy but no LB	980 (3.2)	699 (3.3)	281 (3.0)	
	No prior pregnancy	8,717 (28.3)	5,770 (27.1)	2,947 (31.1)	
	Missing	454 (1.5)	0 (0.0)	454 (4.8)	
Any prior pregnancy ended in SB	Prior pregnancy but no SB	20,736 (67.4)	14,704 (69.0)	6,032 (63.8)	<0.001

For peer review only

Variables	Categories	Total<br><br>N=30,758	Included in regression<br><br>N=21,297	Excluded in regression<br><br>N=9,461	p-value
	Prior SB	1,291 (4.2)	815 (3.8)	476 (5.0)	
	No prior pregnancy	8,717 (28.3)	5,770 (27.1)	2,947 (31.1)	
	Missing	14 (0.0)	8 (0.0)	6 (0.1)	
Any prior pregnancy ended in miscarriage?	Prior pregnancy but no miscarriage	18,554 (60.3)	12,959 (60.8)	5,595 (59.1)	<0.001
	Prior miscarriage	3,478 (11.3)	2,565 (12.0)	913 (9.7)	
	No prior pregnancy	8,717 (28.3)	5,770 (27.1)	2,947 (31.1)	
	Missing	9 (0.0)	3 (0.0)	6 (0.1)	
Any prior pregnancy ended in multiples?	Prior pregnancy but no multiples	21,735 (70.7)	15,383 (72.2)	6,352 (67.1)	<0.001
	Prior multiples	286 (0.9)	135 (0.6)	151 (1.6)	
	No prior pregnancy	8,717 (28.3)	5,770 (27.1)	2,947 (31.1)	
	Missing	20 (0.1)	9 (0.0)	11 (0.1)	
Number of ANC visits	No visit	5,431 (17.7)	3,788 (17.8)	1,643 (17.4)	<0.001
	1 visit	4,047 (13.2)	2,836 (13.3)	1,211 (12.8)	
	2-3 visit	9,443 (30.7)	6,809 (32.0)	2,634 (27.8)	
	4 or more	8,342 (27.1)	6,532 (30.7)	1,810 (19.1)	
	Missing	3,495 (11.4)	1,332 (6.3)	2,163 (22.9)	
Place of Delivery	Home/Maiti	15,669 (50.9)	11,348 (53.3)	4,321 (45.7)	0.002
	HP/Clinic/Hospital	11,038 (35.9)	8,210 (38.6)	2,828 (29.9)	
	Way to Facility/Outdoors	602 (2.0)	439 (2.1)	163 (1.7)	
	Missing	3,449 (11.2)	1,300 (6.1)	2,149 (22.7)	
Bidi or tobacco use in pregnancy	No	30,410 (98.9)	21,060 (98.9)	9,350 (98.8)	0.64
	Yes	348 (1.1)	237 (1.1)	111 (1.2)	
Alcohol use (jaard or rakshi) in pregnancy?	No	30,665 (99.7)	21,230 (99.7)	9,435 (99.7)	0.56
	Yes	93 (0.3)	67 (0.3)	26 (0.3)	
Multiple Birth	Singleton	30,508 (99.2)	21,147 (99.3)	9,361 (98.9)	0.001
	Twin/Triplet	250 (0.8)	150 (0.7)	100 (1.1)	
Sex of the child	Female	14,673 (47.7)	10,178 (47.8)	4,495 (47.5)	0.004
	Male	15,736 (51.2)	10,969 (51.5)	4,767 (50.4)	
	Twin/Triplet	250 (0.8)	150 (0.7)	100 (1.1)	
	Missing	99 (0.3)	0 (0.0)	99 (1.0)	
Preterm Birth	Term	26,130 (85.0)	18,363 (86.2)	7,767 (82.1)	<0.001
	Preterm	4,628 (15.0)	2,934 (13.8)	1,694 (17.9)	
Gestational Age at outcome in weeks (Mean (SD))		39.4 (3.4)	39.5 (2.7)	39.2 (4.6)	<0.001

For peer review only

Table S2-Comparing the Adjusted Risk Ratios for associations between risk factors and spontaneous preterm birth in different models

**Name of Variables**	**Categories**	**Model 1 Unadjusted Model**<br><br>Risk Ratio (95%CI)	**Model 2 Adjusted Model without ANC/Place of Delivery (N=21,297)**<br><br>Risk Ratio (95%CI)	**Model 3 Adjusted- Added ANC**<br><br>**(N=19,965)**<br><br>Risk Ratio (95%CI)	**Model 4 Adjusted- Added ANC and Place of Delivery**<br><br>**(N=19,964)**<br><br>Risk Ratio (95%CI)
Maternal Age at LMP	18 to 35	1.00 [1.00,1.00]	1.00 [1.00,1.00]	1.00 [1.00,1.00]	1.00 [1.00,1.00]
	Less than 18	1.19*** [1.11,1.28]	1.13* [1.02,1.26]	1.11 [1.00,1.24]	1.11* [1.00,1.24]
	More than 35	1.57*** [1.36,1.81]	1.22 [0.98,1.51]	1.20 [0.97,1.49]	1.20 [0.97,1.49]
Caste/Ethnicity Categories	Brahmin and Chhetri	1.00 [1.00,1.00]	1.00 [1.00,1.00]	1.00 [1.00,1.00]	1.00 [1.00,1.00]
	Vaishya	1.33** [1.09,1.62]	1.23 [0.95,1.59]	1.19 [0.92,1.54]	1.20 [0.92,1.54]
	Shudra	1.55*** [1.26,1.90]	1.23 [0.94,1.62]	1.18 [0.90,1.55]	1.18 [0.90,1.55]
	Muslim and others	1.96*** [1.60,2.42]	1.53** [1.16,2.01]	1.53** [1.16,2.01]	1.53** [1.16,2.02]
Mother's Years of Education	No schooling	1.00 [1.00,1.00]	1.00 [1.00,1.00]	1.00 [1.00,1.00]	1.00 [1.00,1.00]
	1 to 5 years	0.86** [0.78,0.95]	0.91 [0.80,1.03]	0.95 [0.83,1.08]	0.95 [0.83,1.08]
	More than 5 years	0.71*** [0.66,0.76]	0.81*** [0.73,0.90]	0.85** [0.77,0.95]	0.85** [0.76,0.94]
Quintiles of Wealth	Poorest	1.00 [1.00,1.00]	1.00 [1.00,1.00]	1.00 [1.00,1.00]	1.00 [1.00,1.00]
	Poorer	0.86*** [0.79,0.93]	0.90* [0.82,1.00]	0.91 [0.83,1.01]	0.91 [0.83,1.01]
	Middle	0.89** [0.82,0.96]	0.95 [0.86,1.05]	0.98 [0.88,1.08]	0.97 [0.88,1.08]
	Richer	0.73*** [0.67,0.79]	0.83** [0.74,0.93]	0.88* [0.78,0.98]	0.87* [0.78,0.98]
	Richest	0.71*** [0.65,0.77]	0.88* [0.78,1.00]	0.91 [0.80,1.03]	0.90 [0.80,1.02]
Mother's height(centimeter)	<145	1.00 [1.00,1.00]	1.00 [1.00,1.00]	1.00 [1.00,1.00]	1.00 [1.00,1.00]
	145-<150	0.93 [0.86,1.01]	0.98 [0.88,1.08]	0.98 [0.89,1.09]	0.99 [0.89,1.09]
	>=150	0.81*** [0.75,0.87]	0.89* [0.81,0.98]	0.90* [0.82,1.00]	0.91 [0.82,1.00]
Parity including both LB and SB, at Enrollment	Parity 1 to 4	1.00 [1.00,1.00]	1.00 [1.00,1.00]	1.00 [1.00,1.00]	1.00 [1.00,1.00]
	More than 4	1.32*** [1.17,1.48]	1.17 [0.99,1.37]	1.11 [0.95,1.31]	1.12 [0.95,1.31]
	Prior Pregnant but parity 0	1.02 [0.85,1.22]	0.92 [0.62,1.37]	1.12 [0.73,1.73]	1.11 [0.72,1.72]
	No Prior Pregnant	1.10** [1.04,1.17]	1.15** [1.04,1.28]	1.20** [1.07,1.34]	1.19** [1.07,1.33]
Interpregnancy Intervals	18 to 36 months	1.00 [1.00,1.00]	1.00 [1.00,1.00]	1.00 [1.00,1.00]	1.00 [1.00,1.00]
	Less than 18 months	1.07 [0.99,1.14]	1.08 [0.99,1.18]	1.09 [0.99,1.19]	1.09 [0.99,1.19]
	More than 36 months	0.98 [0.89,1.09]	0.90 [0.79,1.02]	0.93 [0.82,1.06]	0.93 [0.82,1.06]
	No Prior Pregnancy	1.11** [1.03,1.20]	1.00 [1.00,1.00]	1.00 [1.00,1.00]	1.00 [1.00,1.00]
Any death among prior LB	Prior LB but not died	1.00 [1.00,1.00]	1.00 [1.00,1.00]	1.00 [1.00,1.00]	1.00 [1.00,1.00]
	Prior LB died	1.19*** [1.09,1.29]	1.07 [0.97,1.19]	1.07 [0.96,1.20]	1.07 [0.96,1.20]
	Prior Pregnancy but no LB	1.07 [0.92,1.25]	1.06 [0.75,1.49]	0.97 [0.66,1.41]	0.96 [0.66,1.41]
	No prior pregnancy	1.12*** [1.06,1.19]			

For peer review only

Name of Variables	Categories	Model 1 Unadjusted Model<br>Risk Ratio (95%CI)	Model 2 Adjusted Model without ANC/Place of Delivery (N=21,297)<br>Risk Ratio (95%CI)	Model 3 Adjusted- Added ANC (N=19,965)<br>Risk Ratio (95%CI)	Model 4 Adjusted- Added ANC and Place of Delivery (N=19,964)<br>Risk Ratio (95%CI)
Any prior pregnancy ended in SB	Prior pregnancy but no SB	1.00 [1.00,1.00]			
	Prior SB	1.08 [0.94,1.23]			
	No prior pregnancy	1.08** [1.02,1.15]			
Any prior pregnancy ended in miscarriage	Prior pregnancy but no miscarriage	1.00 [1.00,1.00]			
	Prior miscarriage	0.94 [0.86,1.03]			
	No prior pregnancy	1.07* [1.01,1.13]			
Any prior pregnancy ended in multiples	Prior pregnancy but no multiples	1.00 [1.00,1.00]			
	Prior multiples	1.14 [0.87,1.49]			
	No prior pregnancy	1.08** [1.02,1.14]			
Number of ANC Visits	No visit	1.00 [1.00,1.00]		1.00 [1.00,1.00]	1.00 [1.00,1.00]
	1 visit	0.98 [0.89,1.07]		1.00 [0.90,1.12]	1.00 [0.90,1.12]
	2-3 visit	0.92* [0.85,0.99]		0.94 [0.86,1.03]	0.93 [0.85,1.02]
	4 or more	0.54*** [0.50,0.59]		0.64*** [0.57,0.71]	0.62*** [0.56,0.70]
Place of Delivery	Home/Maiti	1.00 [1.00,1.00]			1.00 [1.00,1.00]
	HP/Clinic/Hospital	0.84*** [0.79,0.89]			1.04 [0.96,1.12]
	Way to Facility/Outdoors	1.28** [1.09,1.51]			1.23 [1.00,1.52]
Multiple Birth	Singleton	1.00 [1.00,1.00]	1.00 [1.00,1.00]	1.00 [1.00,1.00]	1.00 [1.00,1.00]
	Twin/Triplet	3.92*** [3.52,4.38]	4.91*** [4.20,5.75]	4.97*** [4.25,5.82]	4.96*** [4.24,5.81]
Sex of the Child	Female	1.00 [1.00,1.00]	1.00 [1.00,1.00]	1.00 [1.00,1.00]	1.00 [1.00,1.00]
	Male	1.10*** [1.04,1.17]	1.10** [1.02,1.17]	1.08* [1.01,1.16]	1.08* [1.01,1.16]
	Twin/Triplet	4.13*** [3.69,4.63]	1.00 [1.00,1.00]	1.00 [1.00,1.00]	1.00 [1.00,1.00]
STI in at least one visit of 2nd trimester?	No	1.00 [1.00,1.00]			
	Yes	0.99 [0.92,1.07]			
STI in at least one visit of 3rd trimester?	No	1.00 [1.00,1.00]			
	Yes	1.01 [0.92,1.12]			
Respiratory Problem in at least one visit of 2nd trimester?	No	1.00 [1.00,1.00]	1.00 [1.00,1.00]	1.00 [1.00,1.00]	1.00 [1.00,1.00]
	Yes	1.00 [0.94,1.06]	1.08 [1.00,1.16]	1.09* [1.01,1.18]	1.09* [1.01,1.18]
Respiratory Problem in at least one visit of 3rd trimester?	No	1.00 [1.00,1.00]	1.00 [1.00,1.00]	1.00 [1.00,1.00]	1.00 [1.00,1.00]
	Yes	0.85*** [0.79,0.92]	0.86** [0.79,0.94]	0.86** [0.78,0.94]	0.86** [0.78,0.94]
GI Problem in at least one visit of 2nd trimester?	No	1.00 [1.00,1.00]			
	Yes	1.08 [0.98,1.18]			
GI Problem in at least one visit of 3rd trimester?	No	1.00 [1.00,1.00]			
	Yes	1.04 [0.94,1.16]			

For peer review only

Name of Variables	Categories	Model 1 Unadjusted Model<br><br>Risk Ratio (95%CI)	Model 2 Adjusted Model without ANC/Place of Delivery (N=21,297)<br><br>Risk Ratio (95%CI)	Model 3 Adjusted- Added ANC<br><br>(N=19,965)<br><br>Risk Ratio (95%CI)	Model 4 Adjusted- Added ANC and Place of Delivery<br><br>(N=19,964)<br><br>Risk Ratio (95%CI)
Poor appetite, nausea & vomiting in at least one visit of 2nd trimester?	No	1.00 [1.00,1.00]	1.00 [1.00,1.00]	1.00 [1.00,1.00]	1.00 [1.00,1.00]
	Yes	0.81^{***} [0.77,0.86]	0.86^{***} [0.80,0.92]	0.88^{***} [0.82,0.94]	0.88^{***} [0.81,0.94]
Poor appetite, nausea & vomiting in at least one visit of 3rd trimester?	No	1.00 [1.00,1.00]	1.00 [1.00,1.00]	1.00 [1.00,1.00]	1.00 [1.00,1.00]
	Yes	0.88^{**} [0.82,0.95]	0.86^{***} [0.79,0.94]	0.87^{**} [0.79,0.95]	0.87^{**} [0.79,0.95]
Vaginal Bleeding in at least one visit of 2nd trimester?	No	1.00 [1.00,1.00]	1.00 [1.00,1.00]	1.00 [1.00,1.00]	1.00 [1.00,1.00]
	Yes	0.91 [0.71,1.17]	0.84 [0.62,1.16]	0.83 [0.60,1.14]	0.83 [0.60,1.15]
Vaginal Bleeding in at least one visit of 3rd trimester?	No	1.00 [1.00,1.00]	1.00 [1.00,1.00]	1.00 [1.00,1.00]	1.00 [1.00,1.00]
	Yes	1.44^{*} [1.05,1.98]	1.53^{*} [1.08,2.18]	1.49^{*} [1.04,2.13]	1.50^{*} [1.04,2.15]
Swelling in at least one visit of 2nd trimester?	No	1.00 [1.00,1.00]	1.00 [1.00,1.00]	1.00 [1.00,1.00]	1.00 [1.00,1.00]
	Yes	1.32^{***} [1.12,1.55]	1.19 [0.98,1.46]	1.21 [0.98,1.48]	1.21 [0.99,1.48]
Swelling in at least one visit of 3rd trimester?	No	1.00 [1.00,1.00]	1.00 [1.00,1.00]	1.00 [1.00,1.00]	1.00 [1.00,1.00]
	Yes	1.25^{**} [1.09,1.44]	1.37^{***} [1.17,1.60]	1.36^{***} [1.15,1.60]	1.36^{***} [1.15,1.60]
High Systolic BP in one visit of 2nd trimester?	Normal Systolic BP	1.00 [1.00,1.00]	1.00 [1.00,1.00]	1.00 [1.00,1.00]	1.00 [1.00,1.00]
	High Systolic BP	0.89 [0.59,1.34]	0.67 [0.40,1.12]	0.65 [0.39,1.09]	0.65 [0.39,1.09]
High Systolic BP in one visit of 3rd trimester?	Normal Systolic BP	1.00 [1.00,1.00]	1.00 [1.00,1.00]	1.00 [1.00,1.00]	1.00 [1.00,1.00]
	High Systolic BP	1.92^{***} [1.52,2.41]	1.47^{*} [1.08,2.01]	1.49^{*} [1.08,2.07]	1.49^{*} [1.07,2.07]
High Diastolic BP in one visit of 2nd trimester?	Normal diastolic BP	1.00 [1.00,1.00]	1.00 [1.00,1.00]	1.00 [1.00,1.00]	1.00 [1.00,1.00]
	High diastolic BP	1.34^{**} [1.12,1.60]	1.09 [0.85,1.40]	1.06 [0.82,1.37]	1.06 [0.82,1.38]
High Diastolic BP in one visit of 3rd trimester?	Normal diastolic BP	1.00 [1.00,1.00]	1.00 [1.00,1.00]	1.00 [1.00,1.00]	1.00 [1.00,1.00]
	High diastolic BP	1.57^{***} [1.37,1.80]	1.41^{***} [1.17,1.70]	1.35^{**} [1.12,1.64]	1.35^{**} [1.12,1.64]
Average weight in 3rd trimester minus Average weight in 2nd trimester (kg)		0.88^{***} [0.87,0.90]	0.89^{***} [0.87,0.90]	0.89^{***} [0.87,0.90]	0.89^{***} [0.87,0.90]

* $p < 0.05$, ** $p < 0.01$, *** $p < 0.001$

For peer review only
